# Supplementary material for: Tbx1 heterozygosity in the oligodendrocyte lineage shifts myelinated axon composition in the mouse fimbria without behavioral impairments
Source: Mol Brain. 2026 May 20;19:45. doi: 10.1186/s13041-026-01311-4 (PMC13262079; doi:10.1186/s13041-026-01311-4)
Supplement: Supplementary file 1 — Supplementary Material 1 [file 13041_2026_1311_MOESM1_ESM.pdf]

Table S1. Primers used for genotyping.

Mouse line	Forward (5' → 3')	Reverse (5' → 3')	Purpose
Pdgfrα-Cre	oIMR1084 (GCGGTCTGGCAGTAAAAACTATC)	oIMR1085 (GTGAAACAGCATTGCTGTCACTT)	Generic cre - transgene
Pdgfrα-Cre	oIMR7338 (CTAGGCCACAGAATTGAAAGATCT)	oIMR7339 (GTAGGTGGAAATTCTAGCATCATCC)	Generic cre – internal positive control
ROSA-CAG-tdTomato	oIMR9020 (AAGGGAGCTGCAGTGGAGTA)	oIMR9021 (CCGAAAATCTGTGGGAAGTC)	tdTomato wild-type control
ROSA-CAG-tdTomato	oIMR9103 (GGCATTAAAGCAGCGTATCC)	oIMR9105 (CTGTTCCTGTACGGCATGG)	tdTomato mutant
Tbx1$^{+/flox}$	2g 1F (TCTTCTTGGGGCTGTAGACT)	Tbx1 1R (TGACTGTGCTGAAGTGCATC)	LoxP site
Tbx1$^{+/-}$	KO1F (TTGGTGACGATCATCTCGGT)	KO1R (ATGATCTCCGCCGTGTCTAG)<br>Mut2R (AGGTCCCTCGAAGAGGTTCA)	*Tbx1*$^{+/+}$<br>*Tbx1*$^{+/-}$

Table S2. Primers for qRT-PCR

Gene	Assay ID
Tbx1	Mm00448949_m1
Cspg4	Mm00507257_m1
Mag	Mm00487538_m1
Mbp	Mm01266402_m1
Mog	Mm01279062_m1
Plp1	Mm01297210_m1

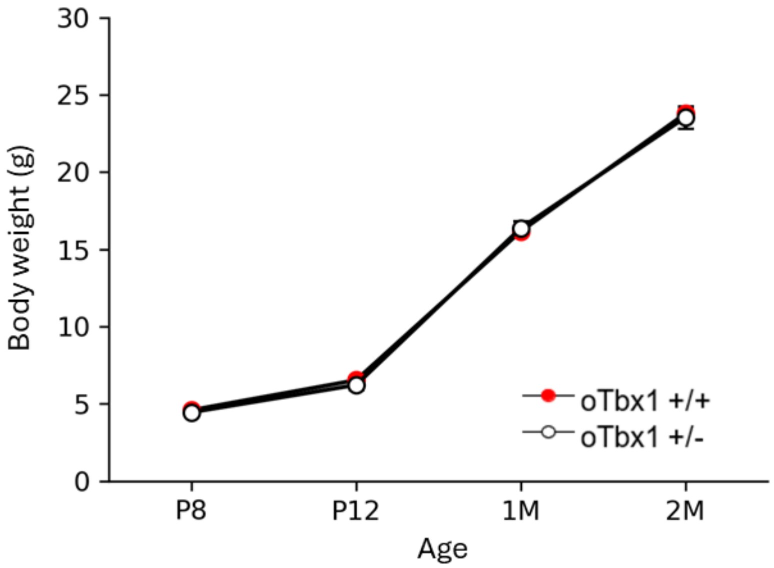

Figure S1. Body weights o*Tbx1*$^{+/+}$ and o*Tbx1*$^{+/-}$ mice. o*Tbx1*$^{+/+}$ and o*Tbx1*$^{+/-}$ mice had indistinguishable body weight increases from P8 to 2 months of age (P8, U = 140, p = 0.800; P12, U = 153, p = 0.466; 1 month, U = 135.5, p = 0.7035; 2 months, U = 102, p = 0.917). o*Tbx1*$^{+/+}$ P8, N = 27, P12; N = 30; 1M, 1 month, N = 27; 2M, 2 months, N = 21: o*Tbx1*$^{+/-}$: P8, N = 11, P12, N = 12; 1M, N = 11; 2M, N = 10.

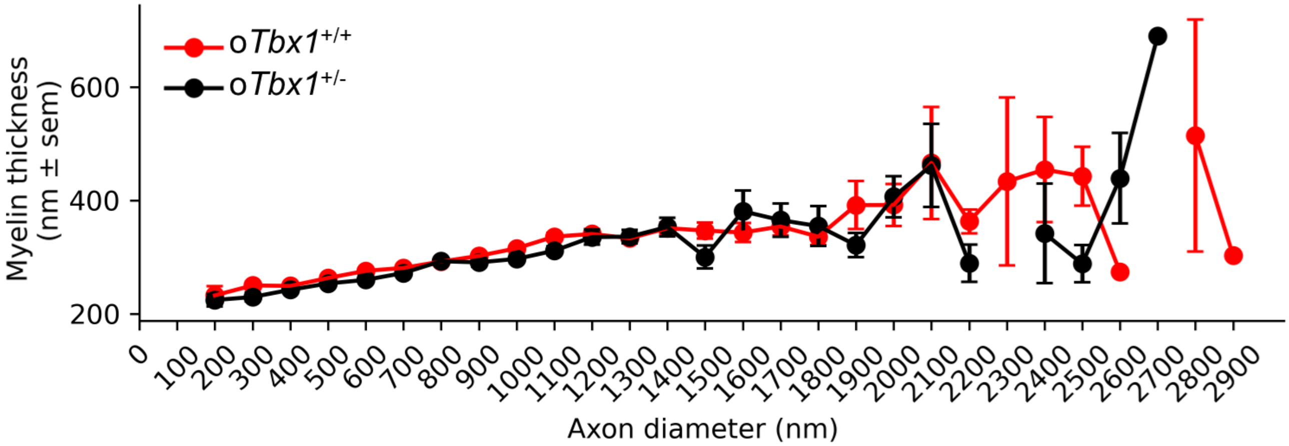

Supplementary Figure S2. The myelin thickness of axons for o*Tbx1*$^{+/-}$ versus o*Tbx1*$^{+/+}$ mice versus axon diameter was similar along the axon diameter range (genotype, $p = 0.931$; genotype × axon diameter, $p = 0.814$).
