# Supplementary material for: Tbx1 heterozygosity in the oligodendrocyte lineage shifts myelinated axon composition in the mouse fimbria without behavioral impairments
Source: Mol Brain. 2026 May 20;19:45. doi: 10.1186/s13041-026-01311-4 (PMC13262079; doi:10.1186/s13041-026-01311-4)
Supplement: Supplementary file 2 — Supplemental Table S3 [file 13041_2026_1311_MOESM2_ESM.pdf]

							Shapiro-Wilk Test				Levene's Test				Linear Mixed Model					Mann Whitney U Test						
Figure	Control Gene	Gene	Treatment	# of Samples	Average	s.e.m	Statistic	p_value	p_adj (FDR BH)	Reject (FDR5%)	Statistic	p_value	p_adj (FDR BH)	Reject (FDR5%)	Source	F	df1	df2	Sig.	Sample 1	Sample 2	Statistic	R (Z score)	p_value	p_adj (FDR BH)	Reject (FDR5%)
Figure 1	Pgk1	Tbx1	control siRNA	6	1	0.049186162	0.93572346	0.624966577	0.805579616	FALSE	1.758766194	0.21744683	0.297607562	FALSE	Corrected Model	2.006348255	11	89	0.03681448	control siRNA	Tbx1 siRNA	7	0.50847518	0.008658009	0.012987013	TRUE
			Tbx1 siRNA	5	0.464725499	0.116052639	0.932068851	0.610545167	0.805579616	FALSE					Gene	0.843979959	5	89	0.52216149							
		Cspg4	control siRNA	9	1	0.066746189	0.960660226	0.805205712	0.878406231	FALSE	1.522725608	0.235026707	0.297607562	FALSE	Treatment	14.66501979	1	89	0.00023857	control siRNA	Tbx1 siRNA	8	0.676423086	0.002756067	0.008268202	TRUE
			Tbx1 siRNA	9	0.685034037	0.037803054	0.980712867	0.967840714	0.967840714	FALSE					Gene * Treatment	0.843979957	5	89	0.522161492							
		MAG	control siRNA	9	1	0.041029197	0.908420558	0.304976014	0.731942433	FALSE	3.473378198	0.080821307	0.297607562	FALSE						control siRNA	Tbx1 siRNA	11	0.613984032	0.007774578	0.012987013	TRUE
			Tbx1 siRNA	9	0.670998004	0.157173093	0.903392871	0.272326671	0.731942433	FALSE																
		MBP	control siRNA	9	1	0.056959774	0.921773656	0.40712653	0.805579616	FALSE	0.679262555	0.421957432	0.421957432	FALSE						control siRNA	Tbx1 siRNA	6	0.718049122	0.00123406	0.00740436	TRUE
			Tbx1 siRNA	9	0.553891554	0.084308916	0.872112121	0.129519549	0.518078195	FALSE																
		MOG	control siRNA	9	1	0.064281655	0.948291674	0.671316347	0.805579616	FALSE	1.89729647	0.18734571	0.297607562	FALSE						control siRNA	Tbx1 siRNA	14	0.551544978	0.018757713	0.018757713	TRUE
			Tbx1 siRNA	9	0.60385684	0.126671201	0.85412067	0.082708168	0.496249006	FALSE																
		Plp1	control siRNA	9	1	0.06060843	0.939293908	0.574485759	0.805579616	FALSE	1.437463429	0.248006301	0.297607562	FALSE						control siRNA	Tbx1 siRNA	14	0.551544978	0.018757713	0.018757713	TRUE
			Tbx1 siRNA	9	0.700871358	0.116118855	0.84502056	0.065659769	0.496249006	FALSE																
	18S	Tbx1	control siRNA	6	1	0.078568382	0.92617953	0.550937859	0.734583812	FALSE	0.009347636	0.925096749	0.925096749	FALSE	Corrected Model	2.450555968	11	89	0.010091634	control siRNA	Tbx1 siRNA	6	0.554700196	0.004329004	0.007774578	TRUE
			Tbx1 siRNA	5	0.440976588	0.066877094	0.97650378	0.915107555	0.915107555	FALSE					Gene	1.101427491	5	89	0.365404553							
		Cspg4	control siRNA	9	1	0.044646855	0.916096471	0.360902781	0.662239178	FALSE	0.016880314	0.898245734	0.925096749	FALSE	Treatment	17.51187144	1	89	6.68502E-05	control siRNA	Tbx1 siRNA	4	0.759675158	0.000493624	0.002961744	TRUE
			Tbx1 siRNA	9	0.687687722	0.045939538	0.919279302	0.386306187	0.662239178	FALSE					Gene * Treatment	1.101427494	5	89	0.365404552							
		MAG	control siRNA	9	1	0.055152176	0.966495138	0.863205301	0.915107555	FALSE	1.450912436	0.24589768	0.643468053	FALSE						control siRNA	Tbx1 siRNA	11	0.613984032	0.007774578	0.007774578	TRUE
			Tbx1 siRNA	9	0.642342516	0.157925591	0.844966625	0.065569561	0.242978818	FALSE																
		MBP	control siRNA	9	1	0.053726606	0.931315789	0.493974756	0.734583812	FALSE	0.206705208	0.655469861	0.925096749	FALSE						control siRNA	Tbx1 siRNA	7	0.697236104	0.00185109	0.00555327	TRUE
			Tbx1 siRNA	9	0.544403139	0.082596116	0.85329064	0.080992939	0.242978818	FALSE																
		MOG	control siRNA	9	1	0.06650766	0.957538993	0.772242353	0.915107555	FALSE	1.178141059	0.293818671	0.643468053	FALSE						control siRNA	Tbx1 siRNA	11	0.613984032	0.007774578	0.007774578	TRUE
			Tbx1 siRNA	9	0.588913841	0.123648773	0.841639086	0.060228776	0.242978818	FALSE																
		Plp1	control siRNA	9	1	0.04046838	0.874188469	0.136290611	0.327097466	FALSE	1.045606567	0.321734027	0.643468053	FALSE						control siRNA	Tbx1 siRNA	11	0.613984032	0.007774578	0.007774578	TRUE
			Tbx1 siRNA	9	0.692323814	0.117369849	0.755073552	0.00620775	0.074492996	FALSE																

Figure	Measurement	Call type	PND	Genotype	# of Samples	Average	s.e.m	Shapiro-Wilk test Statistic	p_value	p_adj(FDR BH)	Rejects(FDR 5%)
Figure 4A	Number	Harmonics	P8	oTbx1+/+	27	25.44444444	6.455928465	0.734571278	1.2196E-05	2.54525E-05	TRUE
				oTbx1+/-	11	18	5.412107639	0.869194567	0.075694919	0.082576275	FALSE
			P12	oTbx1+/+	30	18.53333333	4.41620145	0.769232988	1.89257E-05	3.63374E-05	TRUE
				oTbx1+/-	12	17.41666667	6.962516888	0.741135657	0.002162845	0.002883793	TRUE
		step_down	P8	oTbx1+/+	27	1.518518519	0.424902772	0.641833842	6.62769E-07	2.65108E-06	TRUE
				oTbx1+/-	11	1	0.301511345	0.863411665	0.063748829	0.071161484	FALSE
			P12	oTbx1+/+	30	3.866666667	1.062131162	0.71998024	3.08876E-06	8.23669E-06	TRUE
				oTbx1+/-	12	3.166666667	1.953370565	0.517821491	2.3982E-05	4.42745E-05	TRUE
		short	P8	oTbx1+/+	27	19.92592593	3.665472193	0.834571779	0.000578805	0.000817137	TRUE
				oTbx1+/-	11	12.27272727	2.157822594	0.916108012	0.287544787	0.287544787	FALSE
			P12	oTbx1+/+	30	27.53333333	5.654971027	0.831702948	0.000262676	0.000394014	TRUE
				oTbx1+/-	12	25.91666667	6.770544866	0.883340597	0.096735299	0.098793496	FALSE
		down_fm	P8	oTbx1+/+	27	17.40740741	3.629280755	0.831780851	0.000512651	0.000745674	TRUE
				oTbx1+/-	11	15	4.872930796	0.815746427	0.01516132	0.017749838	TRUE
			P12	oTbx1+/+	30	14.46666667	3.822418243	0.713233709	2.44507E-06	6.90372E-06	TRUE
				oTbx1+/-	12	26.5	10.29599805	0.770105839	0.004369121	0.005242945	TRUE
		up_fm	P8	oTbx1+/+	27	17.7037037	4.965641153	0.629247069	4.62791E-07	2.46822E-06	TRUE
				oTbx1+/-	11	4.909090909	1.889947308	0.722703338	0.000918243	0.001259304	TRUE
			P12	oTbx1+/+	30	27.83333333	7.521934719	0.702088237	1.6735E-06	5.35522E-06	TRUE
				oTbx1+/-	12	23.83333333	7.906492665	0.838153899	0.026294718	0.030051106	TRUE
		two_steps	P8	oTbx1+/+	27	1	0.301940542	0.694654405	3.26996E-06	8.26094E-06	TRUE
				oTbx1+/-	11	1.181818182	0.723855609	0.571349978	1.12326E-05	2.45076E-05	TRUE
			P12	oTbx1+/+	30	0.7	0.215225089	0.653556228	3.51824E-07	2.12738E-06	TRUE
				oTbx1+/-	12	0.583333333	0.192996049	0.768340707	0.004181752	0.005146772	TRUE
		flat	P8	oTbx1+/+	27	17.7037037	4.250294045	0.742479801	1.6037E-05	3.20739E-05	TRUE
				oTbx1+/-	11	12.45454545	3.866790157	0.875244677	0.090499207	0.094433956	FALSE
			P12	oTbx1+/+	30	14.86666667	3.159635267	0.817323029	0.000137908	0.000228261	TRUE
				oTbx1+/-	12	17.41666667	6.344239016	0.755882382	0.003080417	0.003891054	TRUE
		chevron	P8	oTbx1+/+	27	18.11111111	5.570407498	0.636691451	5.71795E-07	2.65108E-06	TRUE
				oTbx1+/-	11	14.09090909	10.5833957	0.4472633	3.54563E-07	2.12738E-06	TRUE
			P12	oTbx1+/+	30	13.56666667	3.444363489	0.749773264	9.03E-06	2.064E-05	TRUE
				oTbx1+/-	12	17.33333333	9.726045424	0.573818922	6.544E-05	0.000112183	TRUE
		complex	P8	oTbx1+/+	27	2.592592593	0.879100906	0.63989383	6.26769E-07	2.65108E-06	TRUE
				oTbx1+/-	11	1.454545455	1.267521822	0.409144521	1.25664E-07	1.20637E-06	TRUE
			P12	oTbx1+/+	30	3.833333333	1.213983027	0.638445973	2.22498E-07	1.77998E-06	TRUE
				oTbx1+/-	12	4.583333333	2.496841439	0.560712337	5.1415E-05	9.14045E-05	TRUE
		step_up	P8	oTbx1+/+	27	3.703703704	0.802089179	0.813462734	0.000236307	0.000365895	TRUE
				oTbx1+/-	11	4.727272727	1.839421397	0.762873292	0.003061853	0.003891054	TRUE
			P12	oTbx1+/+	30	3.2	0.848799016	0.710336447	2.21376E-06	6.64128E-06	TRUE
				oTbx1+/-	12	3.666666667	1.0396192	0.877785325	0.082109146	0.087583089	FALSE
		mult_steps	P8	oTbx1+/+	27	0.074074074	0.051361129	0.29382658	2.32643E-10	5.58344E-09	TRUE
				oTbx1+/-	11	0.090909091	0.090909091	0.344991028	2.2434E-08	2.69208E-07	TRUE
			P12	oTbx1+/+	30	0.066666667	0.046320556	0.275375307	4.40171E-11	2.11282E-09	TRUE
				oTbx1+/-	12	0.083333333	0.083333333	0.326927483	1.20728E-06	4.13925E-06	TRUE
		rev_chevron	P8	oTbx1+/+	27	1.592592593	0.475194545	0.708100617	5.03586E-06	1.20861E-05	TRUE
				oTbx1+/-	11	0.363636364	0.278721995	0.47351861	7.28965E-07	2.69156E-06	TRUE
			P12	oTbx1+/+	30	2.133333333	0.97052342	0.474628329	2.91922E-09	4.67076E-08	TRUE
				oTbx1+/-	12	2.833333333	1.407950011	0.621079922	0.000161712	0.000258739	TRUE

Call type	PND	Levene test Statistic	df1	df2	p_value	p_adj(FDR BH)	Rejects(FDR 5%)
Harmonics	P8	0.588767426	1	36	0.447899604	0.866000524	FALSE
	P12	0.080099472	1	40	0.778622211	0.866000524	FALSE
step_down	P8	0.588478181	1	36	0.448010251	0.866000524	FALSE
	P12	0.194362482	1	40	0.661683974	0.866000524	FALSE
short	P8	3.769928996	1	36	0.060039818	0.866000524	FALSE
	P12	0.789588022	1	40	0.379537318	0.866000524	FALSE
down_fm	P8	0.289267018	1	36	0.594000243	0.866000524	FALSE
	P12	1.591281097	1	40	0.214450379	0.866000524	FALSE
up_fm	P8	2.477639992	1	36	0.124223918	0.866000524	FALSE
	P12	0.168045902	1	40	0.684041632	0.866000524	FALSE
two_steps	P8	0.0764692	1	36	0.783722016	0.866000524	FALSE
	P12	0.113222806	1	40	0.7382634	0.866000524	FALSE
flat	P8	0.680374459	1	36	0.414890086	0.866000524	FALSE
	P12	0.063139102	1	40	0.802887963	0.866000524	FALSE
chevron	P8	0.0673775	1	36	0.796672834	0.866000524	FALSE
	P12	0.345282084	1	40	0.560098838	0.866000524	FALSE
complex	P8	0.506662225	1	36	0.481174865	0.866000524	FALSE
	P12	0.031412363	1	40	0.860217983	0.866000524	FALSE
step_up	P8	0.406675225	1	36	0.527697447	0.866000524	FALSE
	P12	0.034791252	1	40	0.852976553	0.866000524	FALSE
mult_steps	P8	0.028883184	1	36	0.866000524	0.866000524	FALSE
	P12	0.03421728	1	40	0.854180142	0.866000524	FALSE
rev_chevron	P8	2.543697003	1	36	0.119478121	0.866000524	FALSE
	P12	0.044048453	1	40	0.834828853	0.866000524	FALSE

Linear Mixed Model Source	F	df1	df2	Sig.
Corrected Model	6.94053254	47	912	0
Genotype	0.196622137	1	912	0.657566457
PND	6.147480911	1	912	0.013339656
Call type	19.63969375	11	912	0
Genotype * PND	4.835206967	1	912	0.028134731
PND * Call type	1.728524601	11	912	0.062712426
Call type * Genotype	0.707710242	11	912	0.731986987
Genotype * PND * Call type	0.368631844	11	912	0.967949606

Call type	PND	Mann-Witney U test Sample1	Sample2	Statistic	R (Z score)	p_value	p_adj(FDR BH)	Rejects(FDR 5%)
Harmonics	P8	oTbx1+/+	oTbx1+/-	139	0.049603452	0.775387862	0.990742675	FALSE
	P12	oTbx1+/+	oTbx1+/-	180	0	1	1	FALSE
step_down	P8	oTbx1+/+	oTbx1+/-	140	0.044382036	0.799797921	0.990742675	FALSE
	P12	oTbx1+/+	oTbx1+/-	174	0.025776963	0.879981095	0.990742675	FALSE
short	P8	oTbx1+/+	oTbx1+/-	121	0.14358894	0.390409044	0.990742675	FALSE
	P12	oTbx1+/+	oTbx1+/-	168.5	-0.049405846	0.773086018	0.990742675	FALSE
down_fm	P8	oTbx1+/+	oTbx1+/-	145.5	0.015664248	0.949461731	0.990742675	FALSE
	P12	oTbx1+/+	oTbx1+/-	148.5	-0.135329056	0.400911456	0.990742675	FALSE
up_fm	P8	oTbx1+/+	oTbx1+/-	87	0.321117085	0.048527629	0.990742675	FALSE
	P12	oTbx1+/+	oTbx1+/-	175	-0.021480803	0.901682953	0.990742675	FALSE
two_steps	P8	oTbx1+/+	oTbx1+/-	141.5	0.036549912	0.849168654	0.990742675	FALSE
	P12	oTbx1+/+	oTbx1+/-	165	-0.064442408	0.690391077	0.990742675	FALSE
flat	P8	oTbx1+/+	oTbx1+/-	133	0.080931948	0.634139425	0.990742675	FALSE
	P12	oTbx1+/+	oTbx1+/-	170.5	-0.040813525	0.815460496	0.990742675	FALSE
chevron	P8	oTbx1+/+	oTbx1+/-	110.5	0.198413808	0.237616458	0.990742675	FALSE
	P12	oTbx1+/+	oTbx1+/-	160	0.08592321	0.591959808	0.990742675	FALSE
complex	P8	oTbx1+/+	oTbx1+/-	112	0.190581684	0.250721045	0.990742675	FALSE
	P12	oTbx1+/+	oTbx1+/-	161.5	-0.07947897	0.630588582	0.990742675	FALSE
step_up	P8	oTbx1+/+	oTbx1+/-	140	-0.044382036	0.799797921	0.990742675	FALSE
	P12	oTbx1+/+	oTbx1+/-	154.5	-0.109552093	0.50033814	0.990742675	FALSE
mult_steps	P8	oTbx1+/+	oTbx1+/-	146	-0.01305354	0.949461731	0.990742675	FALSE
	P12	oTbx1+/+	oTbx1+/-	177	-0.012888482	0.945286072	0.990742675	FALSE
rev_chevron	P8	oTbx1+/+	oTbx1+/-	104.5	0.229742304	0.168812806	0.990742675	FALSE
	P12	oTbx1+/+	oTbx1+/-	123	-0.24488115	0.116641288	0.990742675	FALSE

Figure	Measurement	Call type	PND	Genotype	# of Samples	Average	s.e.m	Shapiro-Wilk test Statistic	p_value	p_adj(FDR BH)	Rejects(FDR 5%)
Figure 4A	Number	All	P8	oTbx1+/+	27	126.7777778	26.50132144	0.78554976	7.77043E-05	0.000157208	TRUE
				oTbx1+/-	11	85.54545455	23.66407467	0.834964693	0.027126787	0.027126787	TRUE
			P12	oTbx1+/+	30	130.6	27.57400075	0.804300725	7.86042E-05	0.000157208	TRUE
				oTbx1+/-	12	143.3333333	45.18922058	0.79983592	0.009328736	0.012438315	TRUE

PND	Levene test Statistic	df1	df2	p_value	p_adj(FDR BH)	Rejects(FDR 5%)
P8	1.121799982	1	36	0.296586227	0.593172454	FALSE
P12	0.000269074	1	40	0.98699405	0.98699405	FALSE

Linear Mixed Model Source	F	df1	df2	Sig.
Corrected Model	0.636064898	3	76	0.59407046
Genotype	0.155970557	1	76	0.693999978
PND	1.419833311	1	76	0.237138636
Genotype * PND	1.103292412	1	76	0.296873255

PND	Mann-Witney U test Sample1	Sample2	Statistic	R (Z score)	p_value	p_adj(FDR BH)	Rejects(FDR 5%)
P8	oTbx1+/+	oTbx1+/-	126.5	0.114871152	0.504762746	0.73135923	FALSE
P12	oTbx1+/+	oTbx1+/-	166.5	-0.057998167	0.73135923	0.73135923	FALSE

Measurement	Call type	PND	Genotype	# of samples	Average	s.e.m	Shapiro-Wilk test Statistic	p_value	p_adj(FDR BH)	Rejects(FDR 5%)
Number	Harmonics	P8	PdgfraCre;Tbx1+/+	8	9.625	2.672194791	0.919298375	0.42419617	0.449148886	FALSE
			WT;Tbx1+/flox	8	18.125	11.96786993	0.59934529	0.000156224	0.000749877	TRUE
			WT;Tbx1+/+	11	42.27272727	11.79066727	0.863657084	0.064216294	0.087237229	FALSE
		P12	PdgfraCre;Tbx1+/+	9	11.22222222	7.025306988	0.600651882	9.73774E-05	0.000539321	TRUE
			WT;Tbx1+/flox	9	11.66666667	5.525194818	0.765487459	0.008192575	0.016093411	TRUE
			WT;Tbx1+/+	12	29.16666667	8.235946095	0.864345385	0.055433248	0.076753728	FALSE
	step_down	P8	PdgfraCre;Tbx1+/+	8	0.875	0.295048422	0.835213557	0.067235843	0.08964779	FALSE
			WT;Tbx1+/flox	8	1	0.377964473	0.859949135	0.119934837	0.146361157	FALSE
			WT;Tbx1+/+	11	2.363636364	0.956059405	0.720871942	0.000869463	0.002504053	TRUE
		P12	PdgfraCre;Tbx1+/+	9	0.888888889	0.611111111	0.570366044	4.29426E-05	0.000281079	TRUE
			WT;Tbx1+/flox	9	5.111111111	2.6793679	0.695406931	0.001254342	0.003473563	TRUE
			WT;Tbx1+/+	12	5.166666667	1.565892781	0.863856	0.054652247	0.076753728	FALSE
	short	P8	PdgfraCre;Tbx1+/+	8	15.125	4.834539053	0.867444039	0.142278889	0.165227097	FALSE
			WT;Tbx1+/flox	8	13.25	4.087218754	0.849020808	0.093116392	0.117620706	FALSE
			WT;Tbx1+/+	11	28.27272727	7.311374587	0.875466193	0.091090759	0.11711669	FALSE
		P12	PdgfraCre;Tbx1+/+	9	13	3.905124838	0.914834887	0.351194856	0.383121661	FALSE
			WT;Tbx1+/flox	9	33.33333333	13.89844116	0.807207052	0.024673587	0.039477739	TRUE
			WT;Tbx1+/+	12	34.08333333	8.745958663	0.909009298	0.207195733	0.22950912	FALSE
	down_fm	P8	PdgfraCre;Tbx1+/+	8	9.5	3.212698021	0.861074309	0.123069142	0.14768297	FALSE
			WT;Tbx1+/flox	8	11.75	5.456810947	0.738429175	0.006078749	0.012504854	TRUE
			WT;Tbx1+/+	11	27.27272727	6.85842668	0.884384045	0.118227917	0.146361157	FALSE
		P12	PdgfraCre;Tbx1+/+	9	12.88888889	9.075982415	0.490677123	4.96662E-06	5.95994E-05	TRUE
			WT;Tbx1+/flox	9	12.22222222	5.484264809	0.778607516	0.011607818	0.021429818	TRUE
			WT;Tbx1+/+	12	17.33333333	5.805344107	0.805205856	0.010743424	0.020355961	TRUE
	up_fm	P8	PdgfraCre;Tbx1+/+	8	10.375	4.788145704	0.799663368	0.028388995	0.044238038	TRUE
			WT;Tbx1+/flox	8	9.875	3.026298423	0.916331725	0.400811221	0.430722507	FALSE
			WT;Tbx1+/+	11	28.72727273	10.97773856	0.713897791	0.000706475	0.002248161	TRUE
		P12	PdgfraCre;Tbx1+/+	9	10.11111111	7.091552965	0.529781177	1.43217E-05	0.000128896	TRUE
			WT;Tbx1+/flox	9	36.44444444	15.59479179	0.765842027	0.008270225	0.016093411	TRUE
			WT;Tbx1+/+	12	34.66666667	13.49485499	0.683792344	0.000590864	0.00202582	TRUE
	two_steps	P8	PdgfraCre;Tbx1+/+	8	0.125	0.125	0.418398447	1.04723E-06	1.88502E-05	TRUE
			WT;Tbx1+/flox	8	0.75	0.411877236	0.724167693	0.004213657	0.009480728	TRUE
			WT;Tbx1+/+	11	1.818181818	0.600275419	0.82936025	0.022898111	0.037469637	TRUE
		P12	PdgfraCre;Tbx1+/+	9	0.333333333	0.333333333	0.389841298	3.21749E-07	1.1583E-05	TRUE
			WT;Tbx1+/flox	9	0.666666667	0.333333333	0.710163506	0.001864999	0.00497333	TRUE
			WT;Tbx1+/+	12	1	0.40824829	0.7517227	0.002785506	0.006907527	TRUE
	flat	P8	PdgfraCre;Tbx1+/+	8	11.875	3.656879525	0.882816897	0.200332641	0.225374222	FALSE
			WT;Tbx1+/flox	8	6.375	2.744718955	0.801139132	0.029440249	0.044238038	TRUE
			WT;Tbx1+/+	11	30.18181818	8.790302696	0.855776158	0.050750623	0.073080898	FALSE
		P12	PdgfraCre;Tbx1+/+	9	9.444444444	5.283914561	0.634351254	0.000241961	0.001024778	TRUE
			WT;Tbx1+/flox	9	12.44444444	4.972454991	0.834538536	0.05019806	0.073080898	FALSE
			WT;Tbx1+/+	12	20.75	5.615353682	0.900674282	0.161829723	0.184948255	FALSE
	chevron	P8	PdgfraCre;Tbx1+/+	8	11.375	5.990878483	0.735010786	0.005568815	0.011968073	TRUE
			WT;Tbx1+/flox	8	9	6.059231446	0.550872664	4.19244E-05	0.000281079	TRUE
			WT;Tbx1+/+	11	29.63636364	11.76610906	0.702313085	0.000500922	0.00180332	TRUE
		P12	PdgfraCre;Tbx1+/+	9	11.22222222	7.827618088	0.560909917	3.32517E-05	0.000266014	TRUE
			WT;Tbx1+/flox	9	13.22222222	5.340562231	0.814027777	0.029492025	0.044238038	TRUE
			WT;Tbx1+/+	12	15.58333333	5.334931105	0.817775681	0.015028138	0.025762523	TRUE
	complex	P8	PdgfraCre;Tbx1+/+	8	1.5	1	0.604228412	0.000178175	0.000801786	TRUE
			WT;Tbx1+/flox	8	1.625	0.80039053	0.783670395	0.019092425	0.031968712	TRUE
			WT;Tbx1+/+	11	4.090909091	1.928044442	0.714449593	0.000718163	0.002248161	TRUE
		P12	PdgfraCre;Tbx1+/+	9	3.888888889	3.405242871	0.451702288	1.72645E-06	2.48608E-05	TRUE
			WT;Tbx1+/flox	9	4	1.795054936	0.783337582	0.013157116	0.023682808	TRUE
			WT;Tbx1+/+	12	3.666666667	1.275250025	0.815437471	0.014110863	0.024780053	TRUE
	step_up	P8	PdgfraCre;Tbx1+/+	8	3.625	1.450831043	0.863678426	0.130614865	0.154168365	FALSE
			WT;Tbx1+/flox	8	2.75	1.12994943	0.840049789	0.075410948	0.098719786	FALSE
			WT;Tbx1+/+	11	4.454545455	1.503714409	0.760819461	0.002878136	0.006907527	TRUE
		P12	PdgfraCre;Tbx1+/+	9	2.555555556	1.415304356	0.615640291	0.00014599	0.000749877	TRUE
			WT;Tbx1+/flox	9	1.333333333	0.600925213	0.724908399	0.002770316	0.006907527	TRUE
			WT;Tbx1+/+	12	5.083333333	1.694278846	0.780364431	0.00565159	0.011968073	TRUE
	mult_steps	P8	PdgfraCre;Tbx1+/+	8	0	0	1	1	1	FALSE
			WT;Tbx1+/flox	8	0	0	1	1	1	FALSE
			WT;Tbx1+/+	11	0.181818182	0.121967344	0.485720309	1.02086E-06	1.88502E-05	TRUE
		P12	PdgfraCre;Tbx1+/+	9	0	0	1	1	1	FALSE
			WT;Tbx1+/flox	9	0	0	1	1	1	FALSE
			WT;Tbx1+/+	12	0.166666667	0.112366644	0.464652258	9.81123E-06	0.000100916	TRUE
	rev_chevron	P8	PdgfraCre;Tbx1+/+	8	1.125	0.548943791	0.641202	0.000479057	0.00180332	TRUE
			WT;Tbx1+/flox	8	2.25	1.264205001	0.722041682	0.003988818	0.009264352	TRUE
			WT;Tbx1+/+	11	1.454545455	0.651761757	0.720728359	0.000865751	0.002504053	TRUE
		P12	PdgfraCre;Tbx1+/+	9	2.777777778	2.777777778	0.389841298	3.21749E-07	1.1583E-05	TRUE
			WT;Tbx1+/flox	9	1	0.666666667	0.59916075	9.35316E-05	0.000539321	TRUE
			WT;Tbx1+/+	12	2.5	1.276239742	0.646609337	0.000270216	0.001080863	TRUE

Call type	PND	Levene's test Statistic	df1	df2	p_value	p_adj(FDR BH)	Rejects(FDR 5%)
Harmonics	P8	1.65961678	2	24	0.211306457	0.559313096	FALSE
	P12	1.158509452	2	27	0.329073887	0.559313096	FALSE
step_down	P8	1.361546454	2	24	0.275355808	0.559313096	FALSE
	P12	2.413977888	2	27	0.108515742	0.559313096	FALSE
short	P8	1.622768353	2	24	0.218268212	0.559313096	FALSE
	P12	2.151774119	2	27	0.135799339	0.559313096	FALSE
down_fm	P8	1.921139644	2	24	0.168297426	0.559313096	FALSE
	P12	0.180736373	2	27	0.835656843	0.911625647	FALSE
up_fm	P8	1.244964464	2	24	0.305890073	0.559313096	FALSE
	P12	1.093047307	2	27	0.349570685	0.559313096	FALSE
two_steps	P8	2.325091481	2	24	0.119400488	0.559313096	FALSE
	P12	0.817105263	2	27	0.452335248	0.603113664	FALSE
flat	P8	3.818027267	2	24	0.036336836	0.559313096	FALSE
	P12	0.955031557	2	27	0.39741914	0.59612871	FALSE
chevron	P8	0.858879466	2	24	0.436252017	0.603113664	FALSE
	P12	0.058536613	2	27	0.943263064	0.984274502	FALSE
complex	P8	1.111310057	2	24	0.345479196	0.559313096	FALSE
	P12	0.013210185	2	27	0.986883061	0.986883061	FALSE
step_up	P8	0.399734807	2	24	0.674879426	0.809855311	FALSE
	P12	1.307144496	2	27	0.287172685	0.559313096	FALSE
mult_steps	P8	1.580246914	2	24	0.226612028	0.559313096	FALSE
	P12	1.62	2	27	0.216549255	0.559313096	FALSE
rev_chevron	P8	0.474061795	2	24	0.628174436	0.793483498	FALSE
	P12	0.284466288	2	27	0.754642872	0.862448996	FALSE

Generalized Linear Mixed Model Source	F	df1	df2	Sig.
Corrected Model	4.565593657	71	612	0
Genotype	2.526849017	2	612	0.080743877
PND	0.381377322	1	612	0.537096747
Call type	18.61506233	11	612	0
Genotype * PND * Call type	1.47748124	57	612	0.015566306

Call type	PND	Mann-Whitney's U test Sample1	Sample2	Statistic	R (Z score)	p_value	p_adj(FDR BH)	Rejects(FDR 5%)
Harmonics	P8	PdgfraCre;Tbx1+/+	WT;Tbx1+/flox	28.5	0.091893381	0.798445998	0.974374778	FALSE
		PdgfraCre;Tbx1+/+	WT;Tbx1+/+	15.5	-0.539886352	0.02034876	0.488370247	FALSE
		WT;Tbx1+/flox	WT;Tbx1+/+	19	-0.473584519	0.04088275	0.644874038	FALSE
	P12	PdgfraCre;Tbx1+/+	WT;Tbx1+/flox	40	-0.010406509	1	1	FALSE
		PdgfraCre;Tbx1+/+	WT;Tbx1+/+	32.5	-0.333423509	0.147939986	0.644874038	FALSE
		WT;Tbx1+/flox	WT;Tbx1+/+	35	-0.294653333	0.193005137	0.644874038	FALSE
step_down	P8	PdgfraCre;Tbx1+/+	WT;Tbx1+/flox	31	-0.026255252	0.959129759	1	FALSE
		PdgfraCre;Tbx1+/+	WT;Tbx1+/+	34.5	-0.179962117	0.49202191	0.906204631	FALSE
		WT;Tbx1+/flox	WT;Tbx1+/+	35.5	-0.161018736	0.544759334	0.906204631	FALSE
	P12	PdgfraCre;Tbx1+/+	WT;Tbx1+/flox	26.5	-0.291382252	0.258083093	0.743019943	FALSE
		PdgfraCre;Tbx1+/+	WT;Tbx1+/+	26.5	-0.42647193	0.058483312	0.644874038	FALSE
		WT;Tbx1+/flox	WT;Tbx1+/+	47.5	-0.100802456	0.702085531	0.939137333	FALSE
short	P8	PdgfraCre;Tbx1+/+	WT;Tbx1+/flox	30.5	0.039382877	0.959129759	1	FALSE
		PdgfraCre;Tbx1+/+	WT;Tbx1+/+	27	-0.322037473	0.177370273	0.644874038	FALSE
		WT;Tbx1+/flox	WT;Tbx1+/+	27.5	-0.312565783	0.206001429	0.644874038	FALSE
	P12	PdgfraCre;Tbx1+/+	WT;Tbx1+/flox	33.5	-0.145691126	0.604812834	0.926521789	FALSE
		PdgfraCre;Tbx1+/+	WT;Tbx1+/+	34	-0.310161403	0.169421291	0.644874038	FALSE
		WT;Tbx1+/flox	WT;Tbx1+/+	46	-0.124064561	0.60159902	0.926521789	FALSE
down_fm	P8	PdgfraCre;Tbx1+/+	WT;Tbx1+/flox	32	0	1	1	FALSE
		PdgfraCre;Tbx1+/+	WT;Tbx1+/+	22	-0.416754377	0.075414781	0.644874038	FALSE
		WT;Tbx1+/flox	WT;Tbx1+/+	24.5	-0.369395925	0.1288402	0.644874038	FALSE
	P12	PdgfraCre;Tbx1+/+	WT;Tbx1+/flox	37.5	-0.062439054	0.863307281	1	FALSE
		PdgfraCre;Tbx1+/+	WT;Tbx1+/+	46.5	-0.116310526	0.651052972	0.937516279	FALSE
		WT;Tbx1+/flox	WT;Tbx1+/+	44	-0.155080702	0.507876025	0.906204631	FALSE
up_fm	P8	PdgfraCre;Tbx1+/+	WT;Tbx1+/flox	28	-0.105021006	0.720901321	0.939137333	FALSE
		PdgfraCre;Tbx1+/+	WT;Tbx1+/+	26	-0.340980854	0.151755709	0.644874038	FALSE
		WT;Tbx1+/flox	WT;Tbx1+/+	28	-0.303094092	0.206001429	0.644874038	FALSE
	P12	PdgfraCre;Tbx1+/+	WT;Tbx1+/flox	25	-0.32260178	0.190250926	0.644874038	FALSE
		PdgfraCre;Tbx1+/+	WT;Tbx1+/+	29	-0.387701754	0.081543225	0.644874038	FALSE
		WT;Tbx1+/flox	WT;Tbx1+/+	50.5	-0.054278246	0.8620692	1	FALSE
two_steps	P8	PdgfraCre;Tbx1+/+	WT;Tbx1+/flox	23	-0.236297264	0.382284382	0.834075016	FALSE
		PdgfraCre;Tbx1+/+	WT;Tbx1+/+	15.5	-0.539886352	0.02034876	0.488370247	FALSE
		WT;Tbx1+/flox	WT;Tbx1+/+	28	-0.303094092	0.206001429	0.644874038	FALSE
	P12	PdgfraCre;Tbx1+/+	WT;Tbx1+/flox	28.5	-0.249756216	0.340106952	0.78989045	FALSE
		PdgfraCre;Tbx1+/+	WT;Tbx1+/+	38.5	-0.240375088	0.310026197	0.769720213	FALSE
		WT;Tbx1+/flox	WT;Tbx1+/+	50.5	-0.054278246	0.8620692	1	FALSE
flat	P8	PdgfraCre;Tbx1+/+	WT;Tbx1+/flox	20.5	0.301935393	0.278632479	0.743019943	FALSE
		PdgfraCre;Tbx1+/+	WT;Tbx1+/+	28	-0.303094092	0.206001429	0.644874038	FALSE
		WT;Tbx1+/flox	WT;Tbx1+/+	16	-0.530414661	0.02034876	0.488370247	FALSE
	P12	PdgfraCre;Tbx1+/+	WT;Tbx1+/flox	36	-0.093658581	0.730440148	0.939137333	FALSE
		PdgfraCre;Tbx1+/+	WT;Tbx1+/+	32	-0.341177544	0.128534005	0.644874038	FALSE
		WT;Tbx1+/flox	WT;Tbx1+/+	36.5	-0.271391228	0.246909128	0.740727384	FALSE
chevron	P8	PdgfraCre;Tbx1+/+	WT;Tbx1+/flox	31	0.026255252	0.959129759	1	FALSE
		PdgfraCre;Tbx1+/+	WT;Tbx1+/+	27.5	-0.312565783	0.206001429	0.644874038	FALSE
		WT;Tbx1+/flox	WT;Tbx1+/+	22	-0.416754377	0.075414781	0.644874038	FALSE
	P12	PdgfraCre;Tbx1+/+	WT;Tbx1+/flox	33	-0.156097635	0.545701357	0.906204631	FALSE
		PdgfraCre;Tbx1+/+	WT;Tbx1+/+	38	-0.248129123	0.277317729	0.743019943	FALSE
		WT;Tbx1+/flox	WT;Tbx1+/+	51.5	-0.038770175	0.917007451	1	FALSE
complex	P8	PdgfraCre;Tbx1+/+	WT;Tbx1+/flox	27	-0.131276258	0.645376845	0.937516279	FALSE
		PdgfraCre;Tbx1+/+	WT;Tbx1+/+	31.5	-0.23679226	0.351062422	0.78989045	FALSE
		WT;Tbx1+/flox	WT;Tbx1+/+	38.5	-0.104188594	0.71678442	0.939137333	FALSE
	P12	PdgfraCre;Tbx1+/+	WT;Tbx1+/flox	31	-0.197723671	0.436281366	0.906204631	FALSE
		PdgfraCre;Tbx1+/+	WT;Tbx1+/+	39	-0.232621053	0.310026197	0.769720213	FALSE
		WT;Tbx1+/flox	WT;Tbx1+/+	54	0	1	1	FALSE
step_up	P8	PdgfraCre;Tbx1+/+	WT;Tbx1+/flox	30.5	0.039382877	0.959129759	1	FALSE
		PdgfraCre;Tbx1+/+	WT;Tbx1+/+	36.5	-0.142075356	0.599878278	0.926521789	FALSE
		WT;Tbx1+/flox	WT;Tbx1+/+	35	-0.170490427	0.49202191	0.906204631	FALSE
	P12	PdgfraCre;Tbx1+/+	WT;Tbx1+/flox	36.5	0.083252072	0.796174414	0.974374778	FALSE
		PdgfraCre;Tbx1+/+	WT;Tbx1+/+	33	-0.325669474	0.147939986	0.644874038	FALSE
		WT;Tbx1+/flox	WT;Tbx1+/+	28.5	-0.395455789	0.081543225	0.644874038	FALSE
mult_steps	P8	PdgfraCre;Tbx1+/+	WT;Tbx1+/flox	32	0	1	1	FALSE
		PdgfraCre;Tbx1+/+	WT;Tbx1+/+	36	-0.151547046	0.544759334	0.906204631	FALSE
		WT;Tbx1+/flox	WT;Tbx1+/+	36	-0.151547046	0.544759334	0.906204631	FALSE
	P12	PdgfraCre;Tbx1+/+	WT;Tbx1+/flox	40.5	0	1	1	FALSE
		PdgfraCre;Tbx1+/+	WT;Tbx1+/+	45	-0.139572632	0.553791719	0.906204631	FALSE
		WT;Tbx1+/flox	WT;Tbx1+/+	45	-0.139572632	0.553791719	0.906204631	FALSE
rev_chevron	P8	PdgfraCre;Tbx1+/+	WT;Tbx1+/flox	28	-0.105021006	0.720901321	0.939137333	FALSE
		PdgfraCre;Tbx1+/+	WT;Tbx1+/+	39	-0.094716904	0.71678442	0.939137333	FALSE
		WT;Tbx1+/flox	WT;Tbx1+/+	40	0.075773523	0.777989468	0.974374778	FALSE
	P12	PdgfraCre;Tbx1+/+	WT;Tbx1+/flox	33	-0.156097635	0.545701357	0.906204631	FALSE
		PdgfraCre;Tbx1+/+	WT;Tbx1+/+	40	-0.217112982	0.345102575	0.78989045	FALSE
		WT;Tbx1+/flox	WT;Tbx1+/+	47	-0.108556491	0.651052972	0.937516279	FALSE

Measurement	Call type	PND	Genotype	# of samples	Average	s.e.m	Shapiro-Wilk test Statistic	p_value	p_adj(FDR BH)	Rejects(FDR 5%)
Number	All	P8	PdgfraCre;Tbx1+/+	8	75.125	22.42800983	0.919578326	0.426451218	0.426451218	FALSE
			WT;Tbx1+/flox	8	76.75	33.36152082	0.74817822	0.00779798	0.02339394	TRUE
			WT;Tbx1+/+	11	200.7272727	52.31132172	0.852676341	0.046247915	0.069371873	FALSE
		P12	PdgfraCre;Tbx1+/+	9	78.33333333	46.90830298	0.573567292	4.68257E-05	0.000280954	TRUE
			WT;Tbx1+/flox	9	131.4444444	52.89667893	0.822522253	0.036792463	0.069371873	FALSE
			WT;Tbx1+/+	12	169.1666667	44.30897599	0.914009214	0.240072481	0.288086977	FALSE

PND	Levene's test Statistic	df1	df2	p_value	p_adj(FDR BH)	Rejects(FDR 5%)
P8	1.430162095	2	24	0.258940451	0.47061792	FALSE
P12	0.775145688	2	27	0.47061792	0.47061792	FALSE

Repeated Measures 2 Way ANOVA Source	Correction	Type III Sum of Squares	df	Mean Square	F	Sig.
**Within-Subjects**						
PND	Sphericity Assumed	1107.334091	1	1107.334091	0.104222605	0.74961581
PND * Genotype	Sphericity Assumed	24001.49242	2	12000.74621	1.129513707	0.339774875
Error(PND)	Sphericity Assumed	254992.8409	24	10624.7017		
**Between-Subjects**						
Intercept		787339.8818	1	787339.8818	25.39936419	3.75838E-05
Genotype		112088.5152	2	56044.25758	1.807972061	0.18561632
Error		743961.8182	24	30998.40909		

PND	Student's t-test Sample1	Sample2	Statistic	dof	Cohen's d	p_value	p_adj(FDR BH)	Rejects(FDR 5%)
P8	PdgfraCre;Tbx1+/+	WT;Tbx1+/flox	-0.040423302	14	-0.020211651	0.968326472	0.968326472	FALSE
	PdgfraCre;Tbx1+/+	WT;Tbx1+/+	-1.942536002	17	-0.902619145	0.068825485	0.25684836	FALSE
	WT;Tbx1+/flox	WT;Tbx1+/+	-1.825049546	17	-0.848027866	0.08561612	0.25684836	FALSE
P12	PdgfraCre;Tbx1+/+	WT;Tbx1+/flox	-0.75122117	16	-0.354129056	0.463426442	0.695139663	FALSE
	PdgfraCre;Tbx1+/+	WT;Tbx1+/+	-1.389484067	19	-0.612704882	0.180755026	0.361510052	FALSE
	WT;Tbx1+/flox	WT;Tbx1+/+	-0.549424201	19	-0.2422733	0.589114473	0.706937367	FALSE

Figure	Measurement	Call type	PND	Genotype	# of Samples	Average	s.e.m	Shapiro-Wilk test Statistic	p_value	p_adj(FDR BH)	Rejects(FDR 5%)
Figure 4B	Proportion	Harmonics	P8	oTbx1+/+	27	17.03450018	2.203565508	0.951793015	0.236913264	0.27736187	FALSE
				oTbx1+/-	11	19.61154304	3.599706317	0.960571766	0.778805196	0.79537552	FALSE
			P12	oTbx1+/+	30	13.16752619	3.364708074	0.59169817	5.78851E-08	3.96926E-07	TRUE
				oTbx1+/-	12	8.505399165	1.955013986	0.873135149	0.07162638	0.090475427	FALSE
		step_down	P8	oTbx1+/+	27	1.336688279	0.313639637	0.787106931	8.25136E-05	0.000220036	TRUE
				oTbx1+/-	11	1.573785836	0.594457163	0.750182569	0.002090028	0.004180056	TRUE
			P12	oTbx1+/+	30	1.789236696	0.427419086	0.770336628	1.97568E-05	5.92704E-05	TRUE
				oTbx1+/-	12	9.940976217	8.252655216	0.398949146	3.48338E-06	1.28617E-05	TRUE
		short	P8	oTbx1+/+	27	23.4473742	4.012502925	0.666321874	1.3633E-06	5.45318E-06	TRUE
				oTbx1+/-	11	21.62772654	4.72041667	0.830675006	0.023827234	0.035740851	TRUE
			P12	oTbx1+/+	30	23.23444672	2.727284442	0.958858669	0.289563179	0.317734805	FALSE
				oTbx1+/-	12	22.86873026	4.926486399	0.944190502	0.55418241	0.578277298	FALSE
		down_fm	P8	oTbx1+/+	27	12.75630401	1.76961371	0.920927048	0.041565027	0.058680038	FALSE
				oTbx1+/-	11	21.16328548	4.9646338	0.884678006	0.11924167	0.146758978	FALSE
			P12	oTbx1+/+	30	10.37298456	1.542082257	0.928056836	0.043611676	0.059699769	FALSE
				oTbx1+/-	12	13.01185528	3.11935555	0.931758583	0.399125427	0.425733789	FALSE
		up_fm	P8	oTbx1+/+	27	13.05872025	1.780737559	0.92226398	0.044774827	0.059699769	FALSE
				oTbx1+/-	11	6.202009894	1.944137346	0.855210662	0.04989852	0.064733215	FALSE
			P12	oTbx1+/+	30	17.31720233	2.688345341	0.892989337	0.005683312	0.010103665	TRUE
				oTbx1+/-	12	14.90117688	3.627231491	0.915457904	0.250485063	0.286268643	FALSE
		two_steps	P8	oTbx1+/+	27	1.22818806	0.583431602	0.439589798	4.31981E-09	6.9117E-08	TRUE
				oTbx1+/-	11	0.657609323	0.30689217	0.709964395	0.000628547	0.001436678	TRUE
			P12	oTbx1+/+	30	0.376106479	0.140892041	0.56127727	2.53783E-08	2.03026E-07	TRUE
				oTbx1+/-	12	0.420168462	0.189210931	0.706350029	0.000970717	0.002117929	TRUE
		flat	P8	oTbx1+/+	27	13.18848553	1.439854436	0.955547333	0.291256905	0.317734805	FALSE
				oTbx1+/-	11	13.58957049	2.677180408	0.971073091	0.897150159	0.897150159	FALSE
			P12	oTbx1+/+	30	10.75966512	1.248052332	0.916827679	0.022208858	0.03438791	TRUE
				oTbx1+/-	12	9.627772862	1.980798048	0.90607655	0.189971969	0.227966362	FALSE
		chevron	P8	oTbx1+/+	27	11.99601344	2.252797934	0.893206954	0.009383468	0.015531257	TRUE
				oTbx1+/-	11	8.606354313	3.605578163	0.701831937	0.000493834	0.001185202	TRUE
			P12	oTbx1+/+	30	7.944668927	1.340965584	0.883133352	0.00332987	0.006147453	TRUE
				oTbx1+/-	12	5.704118406	2.467406	0.713398993	0.001137725	0.002374382	TRUE
		complex	P8	oTbx1+/+	27	1.359067236	0.357237975	0.747017026	1.88041E-05	5.92704E-05	TRUE
				oTbx1+/-	11	0.594390633	0.456151172	0.472920179	7.17045E-07	4.30227E-06	TRUE
			P12	oTbx1+/+	30	1.444040234	0.348567383	0.776604891	2.52732E-05	7.13597E-05	TRUE
				oTbx1+/-	12	2.213403834	0.819843897	0.755306244	0.003037653	0.005832293	TRUE
		step_up	P8	oTbx1+/+	27	3.459543469	0.754843072	0.828750014	0.000449811	0.001136364	TRUE
				oTbx1+/-	11	6.030970473	2.091188206	0.792426586	0.007478565	0.012820397	TRUE
			P12	oTbx1+/+	30	2.930012431	0.802546816	0.690653443	1.14398E-06	5.26814E-06	TRUE
				oTbx1+/-	12	2.522648912	0.867273801	0.814436078	0.013736397	0.021978235	TRUE
		mult_steps	P8	oTbx1+/+	27	0.053993663	0.038446941	0.300173998	2.61841E-10	6.28418E-09	TRUE
				oTbx1+/-	11	0.031897927	0.031897927	0.344991028	2.2434E-08	2.03026E-07	TRUE
			P12	oTbx1+/+	30	0.016955782	0.011903467	0.282110274	5.00231E-11	2.40111E-09	TRUE
				oTbx1+/-	12	0.020627063	0.020627063	0.326927483	1.20728E-06	5.26814E-06	TRUE
		rev_chevron	P8	oTbx1+/+	27	1.081121699	0.315727719	0.719082952	7.22694E-06	2.47781E-05	TRUE
				oTbx1+/-	11	0.31085605	0.230553927	0.488995969	1.11768E-06	5.26814E-06	TRUE
			P12	oTbx1+/+	30	0.647154535	0.267451787	0.507237554	6.39587E-09	7.67505E-08	TRUE
				oTbx1+/-	12	1.929789318	0.609606267	0.845055282	0.031914786	0.046421506	TRUE

Call type	PND	Levene test Statistic	df1	df2	p_value	p_adj(FDR BH)	Rejects(FDR 5%)
Harmonics	P8	0.007209331	1	36	0.932805151	0.958177206	FALSE
	P12	0.873593574	1	40	0.355570832	0.948188885	FALSE
step_down	P8	0.149229077	1	36	0.701546982	0.958177206	FALSE
	P12	2.489159983	1	40	0.122509658	0.586275644	FALSE
short	P8	0.002788636	1	36	0.958177206	0.958177206	FALSE
	P12	0.18394001	1	40	0.670309944	0.958177206	FALSE
down_fm	P8	4.472020458	1	36	0.041444496	0.497333956	FALSE
	P12	0.954744089	1	40	0.334387409	0.948188885	FALSE
up_fm	P8	5.242724489	1	36	0.028006319	0.497333956	FALSE
	P12	0.013930753	1	40	0.9066357	0.958177206	FALSE
two_steps	P8	0.367415161	1	36	0.548219905	0.958177206	FALSE
	P12	0.031791176	1	40	0.859386723	0.958177206	FALSE
flat	P8	0.064044466	1	36	0.801653642	0.958177206	FALSE
	P12	0.005399042	1	40	0.941791831	0.958177206	FALSE
chevron	P8	0.562260857	1	36	0.458223333	0.958177206	FALSE
	P12	0.121536249	1	40	0.72920259	0.958177206	FALSE
complex	P8	1.462744932	1	36	0.234376731	0.803577363	FALSE
	P12	0.463312445	1	40	0.500001335	0.958177206	FALSE
step_up	P8	2.986552004	1	36	0.092528851	0.586275644	FALSE
	P12	0.337019135	1	40	0.564811412	0.958177206	FALSE
mult_steps	P8	0.119494266	1	36	0.731595596	0.958177206	FALSE
	P12	0.025753757	1	40	0.873310885	0.958177206	FALSE
rev_chevron	P8	2.201621745	1	36	0.146568911	0.586275644	FALSE
	P12	2.868746639	1	40	0.098089764	0.586275644	FALSE

Linear Mixed Model Source	F	df1	df2	Sig.
Corrected Model	12.69567482	47	912	0
Genotype	0.010709424	1	912	0.917599736
PND	1.295840311	1	912	0.255273381
Call type	39.53182246	11	912	0
Genotype * PND	0.010709424	1	912	0.917599736
PND * Call type	2.796512537	11	912	0.001369316
Call type * Genotype	1.365715759	11	912	0.183698653
Genotype * PND * Call type	0.796636894	11	912	0.643654777

Call type	PND	Mann-Witney U test Sample1	Sample2	Statistic	R (Z score)	p_value	p_adj(FDR BH)	Rejects(FDR 5%)
Harmonics	P8	oTbx1+/+	oTbx1+/-	129	-0.101817612	0.546349031	0.974718567	FALSE
	P12	oTbx1+/+	oTbx1+/-	157	0.098811692	0.536091044	0.974718567	FALSE
step_down	P8	oTbx1+/+	oTbx1+/-	143.5	-0.02610708	0.899119881	0.974718567	FALSE
	P12	oTbx1+/+	oTbx1+/-	176.5	-0.015036562	0.945286072	0.974718567	FALSE
short	P8	oTbx1+/+	oTbx1+/-	142	0.033939204	0.849168654	0.974718567	FALSE
	P12	oTbx1+/+	oTbx1+/-	174	-0.025776963	0.879981095	0.974718567	FALSE
down_fm	P8	oTbx1+/+	oTbx1+/-	105	-0.227131596	0.168812806	0.974718567	FALSE
	P12	oTbx1+/+	oTbx1+/-	156.5	-0.100959772	0.536091044	0.974718567	FALSE
up_fm	P8	oTbx1+/+	oTbx1+/-	84	0.336781333	0.038051176	0.45661411	FALSE
	P12	oTbx1+/+	oTbx1+/-	165.5	0.062294328	0.710773696	0.974718567	FALSE
two_steps	P8	oTbx1+/+	oTbx1+/-	137.5	0.057435576	0.751191124	0.974718567	FALSE
	P12	oTbx1+/+	oTbx1+/-	158	-0.094515531	0.5544243	0.974718567	FALSE
flat	P8	oTbx1+/+	oTbx1+/-	146.5	-0.010442832	0.974718567	0.974718567	FALSE
	P12	oTbx1+/+	oTbx1+/-	164	0.068738568	0.670224551	0.974718567	FALSE
chevron	P8	oTbx1+/+	oTbx1+/-	120	0.148810356	0.372960146	0.974718567	FALSE
	P12	oTbx1+/+	oTbx1+/-	149	0.133180976	0.400911456	0.974718567	FALSE
complex	P8	oTbx1+/+	oTbx1+/-	110.5	0.198413808	0.237616458	0.974718567	FALSE
	P12	oTbx1+/+	oTbx1+/-	153	-0.115996334	0.465855659	0.974718567	FALSE
step_up	P8	oTbx1+/+	oTbx1+/-	128	-0.107039028	0.525351863	0.974718567	FALSE
	P12	oTbx1+/+	oTbx1+/-	174	-0.025776963	0.879981095	0.974718567	FALSE
mult_steps	P8	oTbx1+/+	oTbx1+/-	147	-0.007832124	0.974718567	0.974718567	FALSE
	P12	oTbx1+/+	oTbx1+/-	177	-0.012888482	0.945286072	0.974718567	FALSE
rev_chevron	P8	oTbx1+/+	oTbx1+/-	107	0.216688764	0.189914135	0.974718567	FALSE
	P12	oTbx1+/+	oTbx1+/-	99	-0.347989002	0.023533765	0.45661411	FALSE

Measurement	Call type	PND	Genotype	# of samples	Average	s.e.m	Shapiro-Wilk test Statistic	p_value	p_adj(FDR BH)	Rejects(FDR 5%)
Proportion	Harmonics	P8	PdgfraCre;Tbx1+/+	8	14.33386512	3.180619627	0.920521609	0.434110293	0.529761713	FALSE
			WT;Tbx1+/flox	8	13.69827654	5.213100979	0.825142512	0.052831697	0.105663394	FALSE
			WT;Tbx1+/+	11	21.42494286	2.955550922	0.956472392	0.727051807	0.805349694	FALSE
		P12	PdgfraCre;Tbx1+/+	9	19.77849016	10.32058624	0.614034689	0.000139793	0.000915012	TRUE
			WT;Tbx1+/flox	9	5.644679288	1.918519292	0.860758774	0.097722904	0.171610953	FALSE
			WT;Tbx1+/+	12	13.85143838	2.762225592	0.888610048	0.113077876	0.1893397	FALSE
	step_down	P8	PdgfraCre;Tbx1+/+	8	1.794530722	0.846007146	0.772387788	0.014392817	0.041451314	TRUE
			WT;Tbx1+/flox	8	1.290974418	0.534434464	0.816551909	0.0429211	0.093646035	FALSE
			WT;Tbx1+/+	11	1.0369584	0.305974108	0.896482842	0.167368171	0.262860212	FALSE
		P12	PdgfraCre;Tbx1+/+	9	0.512440844	0.392071531	0.528195976	1.37202E-05	0.000142397	TRUE
			WT;Tbx1+/flox	9	2.584253777	0.986144531	0.808525695	0.025540655	0.063411281	FALSE
			WT;Tbx1+/+	12	2.150570773	0.650897914	0.881745505	0.092280528	0.167826246	FALSE
	short	P8	PdgfraCre;Tbx1+/+	8	27.79609334	7.835750345	0.802567038	0.030493177	0.073183624	FALSE
			WT;Tbx1+/flox	8	30.52798165	10.4520263	0.674664094	0.001160659	0.004915731	TRUE
			WT;Tbx1+/+	11	15.13513667	1.98602887	0.910698292	0.248577402	0.357951459	FALSE
		P12	PdgfraCre;Tbx1+/+	9	29.41714216	6.77204175	0.954397752	0.73826333	0.805378178	FALSE
			WT;Tbx1+/flox	9	21.11850489	4.315377521	0.818225622	0.032903072	0.076420038	FALSE
			WT;Tbx1+/+	12	20.18438151	3.177084685	0.968703162	0.896753634	0.949503848	FALSE
	down_fm	P8	PdgfraCre;Tbx1+/+	8	9.902998402	1.798693708	0.925830506	0.478929706	0.574715647	FALSE
			WT;Tbx1+/flox	8	12.95875965	4.301199919	0.91573224	0.396200038	0.509400048	FALSE
			WT;Tbx1+/+	11	14.68419488	2.820054979	0.931509541	0.426325541	0.529231706	FALSE
		P12	PdgfraCre;Tbx1+/+	9	15.1261745	3.538818708	0.937379276	0.554598449	0.633826799	FALSE
			WT;Tbx1+/flox	9	6.768936551	2.39904953	0.877183695	0.1466369	0.239951291	FALSE
			WT;Tbx1+/+	12	9.511128113	1.791519021	0.945887631	0.577853788	0.650085512	FALSE
	up_fm	P8	PdgfraCre;Tbx1+/+	8	10.52722533	3.589529281	0.898985786	0.282937423	0.399441068	FALSE
			WT;Tbx1+/flox	8	15.51934247	3.299813459	0.929283224	0.509574132	0.594583706	FALSE
			WT;Tbx1+/+	11	13.11026403	2.688376967	0.960336023	0.775876897	0.833778158	FALSE
		P12	PdgfraCre;Tbx1+/+	9	10.7373311	3.075778662	0.912907898	0.336760896	0.449014528	FALSE
			WT;Tbx1+/flox	9	24.26204578	6.725552181	0.910146573	0.316901753	0.430508042	FALSE
			WT;Tbx1+/+	12	17.04347316	3.399092861	0.941065847	0.512002636	0.594583706	FALSE
	two_steps	P8	PdgfraCre;Tbx1+/+	8	0.102459016	0.102459016	0.418398447	1.04723E-06	2.51336E-05	TRUE
			WT;Tbx1+/flox	8	2.204948931	1.891510087	0.486985371	7.19813E-06	0.000103653	TRUE
			WT;Tbx1+/+	11	1.33652855	0.427577641	0.831222503	0.024225071	0.063411281	FALSE
		P12	PdgfraCre;Tbx1+/+	9	0.074906367	0.074906367	0.389841298	3.21749E-07	1.1583E-05	TRUE
			WT;Tbx1+/flox	9	0.455038711	0.205119656	0.782599951	0.012902722	0.038708165	TRUE
			WT;Tbx1+/+	12	0.542807388	0.309332544	0.587077317	8.38703E-05	0.000670963	TRUE
	flat	P8	PdgfraCre;Tbx1+/+	8	17.93017709	2.717057881	0.917100523	0.406781173	0.513828851	FALSE
			WT;Tbx1+/flox	8	8.633543264	2.89229413	0.820293101	0.04699594	0.099520814	FALSE
			WT;Tbx1+/+	11	13.05266786	1.437946293	0.927701172	0.388068488	0.50801693	FALSE
		P12	PdgfraCre;Tbx1+/+	9	10.49163426	2.2312487	0.893212814	0.215226195	0.322839292	FALSE
			WT;Tbx1+/flox	9	7.38091094	2.106347882	0.89566068	0.22790882	0.33488643	FALSE
			WT;Tbx1+/+	12	13.4947539	1.915033856	0.923022175	0.311949399	0.430508042	FALSE
	chevron	P8	PdgfraCre;Tbx1+/+	8	11.11468622	3.975984082	0.839208898	0.073925068	0.143854186	FALSE
			WT;Tbx1+/flox	8	8.28992559	2.59542819	0.854717441	0.106307995	0.182242278	FALSE
			WT;Tbx1+/+	11	15.33231531	4.329267764	0.896602764	0.167938469	0.262860212	FALSE
		P12	PdgfraCre;Tbx1+/+	9	8.48801746	3.058254192	0.834102105	0.049637303	0.102111023	FALSE
			WT;Tbx1+/flox	9	6.795574321	1.76969935	0.807793859	0.025055817	0.063411281	FALSE
			WT;Tbx1+/+	12	8.398978482	2.216508759	0.903793823	0.177537843	0.271972866	FALSE
	complex	P8	PdgfraCre;Tbx1+/+	8	0.976069158	0.63900965	0.568475715	6.77437E-05	0.000609693	TRUE
			WT;Tbx1+/flox	8	1.68881032	0.777065407	0.807628446	0.034526318	0.077684215	FALSE
			WT;Tbx1+/+	11	1.397798141	0.529558045	0.775672329	0.004505088	0.017071912	TRUE
		P12	PdgfraCre;Tbx1+/+	9	1.35985243	0.834393015	0.639968592	0.000281573	0.00153755	TRUE
			WT;Tbx1+/flox	9	1.639512949	0.546565381	0.736084074	0.003737285	0.014949138	TRUE
			WT;Tbx1+/+	12	1.360576551	0.50435214	0.777657114	0.005278143	0.019001316	TRUE
	step_up	P8	PdgfraCre;Tbx1+/+	8	4.58948929	1.934601202	0.843751974	0.082294537	0.155926491	FALSE
			WT;Tbx1+/flox	8	3.489325269	1.336233123	0.849076133	0.093236803	0.167826246	FALSE
			WT;Tbx1+/+	11	2.6161052	0.811090133	0.802232956	0.01006582	0.031589754	TRUE
		P12	PdgfraCre;Tbx1+/+	9	3.389790988	1.520322048	0.773329537	0.010091172	0.031589754	TRUE
			WT;Tbx1+/flox	9	0.834752633	0.532565223	0.603414342	0.000104925	0.000755456	TRUE
			WT;Tbx1+/+	12	4.156623362	1.54243506	0.656505111	0.000331387	0.001590656	TRUE
	mult_steps	P8	PdgfraCre;Tbx1+/+	8	0	0	1	1	1	FALSE
			WT;Tbx1+/flox	8	0	0	1	1	1	FALSE
			WT;Tbx1+/+	11	0.132529899	0.091610889	0.511703575	2.09968E-06	3.77942E-05	TRUE
		P12	PdgfraCre;Tbx1+/+	9	0	0	1	1	1	FALSE
			WT;Tbx1+/flox	9	0	0	1	1	1	FALSE
			WT;Tbx1+/+	12	0.042389454	0.028911445	0.485515454	1.38441E-05	0.000142397	TRUE
	rev_chevron	P8	PdgfraCre;Tbx1+/+	8	0.932406314	0.485217797	0.736969104	0.005855571	0.020076244	TRUE
			WT;Tbx1+/flox	8	1.698111905	0.810075328	0.792567662	0.02382121	0.063411281	FALSE
			WT;Tbx1+/+	11	0.740558193	0.375948876	0.684843395	0.000298968	0.00153755	TRUE
		P12	PdgfraCre;Tbx1+/+	9	0.624219725	0.624219725	0.389841298	3.21749E-07	1.1583E-05	TRUE
			WT;Tbx1+/flox	9	0.29356793	0.173122527	0.653259574	0.000403018	0.001813582	TRUE
			WT;Tbx1+/+	12	0.929545596	0.476189549	0.644326098	0.000257894	0.00153755	TRUE

Call type	PND	Levene's test Statistic	df1	df2	p_value	p_adj(FDR BH)	Rejects(FDR 5%)
Harmonics	P8	1.84713931	2	24	0.179413127	0.51429221	FALSE
	P12	1.199438418	2	27	0.316917223	0.51429221	FALSE
step_down	P8	1.151437041	2	24	0.333040024	0.51429221	FALSE
	P12	3.768584833	2	27	0.036019673	0.51429221	FALSE
short	P8	1.027435303	2	24	0.373136602	0.51429221	FALSE
	P12	2.18520834	2	27	0.131943177	0.51429221	FALSE
down_fm	P8	1.301310466	2	24	0.290697828	0.51429221	FALSE
	P12	1.089462096	2	27	0.350732163	0.51429221	FALSE
up_fm	P8	0.222556444	2	24	0.802103358	0.875021845	FALSE
	P12	1.438522228	2	27	0.254889982	0.51429221	FALSE
two_steps	P8	1.00441335	2	24	0.381141088	0.51429221	FALSE
	P12	1.013541908	2	27	0.376326518	0.51429221	FALSE
flat	P8	0.524216516	2	24	0.598643331	0.75618105	FALSE
	P12	0.040346651	2	27	0.960514231	0.960514231	FALSE
chevron	P8	0.991480484	2	24	0.385719158	0.51429221	FALSE
	P12	1.856606996	2	27	0.175598067	0.51429221	FALSE
complex	P8	0.3998853	2	24	0.674781143	0.771178449	FALSE
	P12	0.065201783	2	27	0.937025452	0.960514231	FALSE
step_up	P8	2.38559384	2	24	0.113511907	0.51429221	FALSE
	P12	1.256794019	2	27	0.300686319	0.51429221	FALSE
mult_steps	P8	1.488233273	2	24	0.245874864	0.51429221	FALSE
	P12	1.582954373	2	27	0.223840796	0.51429221	FALSE
rev_chevron	P8	0.998042961	2	24	0.383388724	0.51429221	FALSE
	P12	0.46841942	2	27	0.630983165	0.757179799	FALSE

Linear Mixed Model Source	F	df1	df2	Sig.
Corrected Model	7.678337945	71	612	0
Genotype	0.567660277	2	612	0.567148336
PND	1.529321287	1	612	0.216688691
Call type	42.41123303	11	612	0
Genotype * PND	0.567660277	2	612	0.567148336
PND * Call type	0.791648519	11	612	0.648626623
Call type * Genotype	1.940248357	22	612	0.006372406
Genotype * PND * Call type	1.033284006	22	612	0.419829765

Call type	PND	Mann-Whitney's U test Sample1	Sample2	Statistic	R (Z score)	p_value	p_adj(FDR BH)	Rejects(FDR 5%)
Harmonics	P8	PdgfraCre;Tbx1+/+	WT;Tbx1+/flox	29.5	0.065638129	0.878477078	1	FALSE
		PdgfraCre;Tbx1+/+	WT;Tbx1+/+	25	-0.359924234	0.1288402	0.7730412	FALSE
		WT;Tbx1+/flox	WT;Tbx1+/+	32	-0.227320569	0.351062422	0.901457961	FALSE
	P12	PdgfraCre;Tbx1+/+	WT;Tbx1+/flox	25	0.32260178	0.190250926	0.901457961	FALSE
		PdgfraCre;Tbx1+/+	WT;Tbx1+/+	47	-0.108556491	0.651052972	0.901457961	FALSE
		WT;Tbx1+/flox	WT;Tbx1+/+	26.5	-0.42647193	0.058483312	0.701799748	FALSE
step_down	P8	PdgfraCre;Tbx1+/+	WT;Tbx1+/flox	29.5	0.065638129	0.878477078	1	FALSE
		PdgfraCre;Tbx1+/+	WT;Tbx1+/+	38.5	0.104188594	0.71678442	0.955712559	FALSE
		WT;Tbx1+/flox	WT;Tbx1+/+	42.5	0.028415071	0.967849488	1	FALSE
	P12	PdgfraCre;Tbx1+/+	WT;Tbx1+/flox	24	-0.343414798	0.161497326	0.89444673	FALSE
		PdgfraCre;Tbx1+/+	WT;Tbx1+/+	27	-0.418717895	0.058483312	0.701799748	FALSE
		WT;Tbx1+/flox	WT;Tbx1+/+	53	0.01550807	0.97227231	1	FALSE
short	P8	PdgfraCre;Tbx1+/+	WT;Tbx1+/flox	31	-0.026255252	0.959129759	1	FALSE
		PdgfraCre;Tbx1+/+	WT;Tbx1+/+	27.5	0.312565783	0.206001429	0.901457961	FALSE
		WT;Tbx1+/flox	WT;Tbx1+/+	24	0.378867615	0.108650208	0.74285928	FALSE
	P12	PdgfraCre;Tbx1+/+	WT;Tbx1+/flox	32	0.176910653	0.489428219	0.901457961	FALSE
		PdgfraCre;Tbx1+/+	WT;Tbx1+/+	41	0.201604912	0.3824448	0.901457961	FALSE
		WT;Tbx1+/flox	WT;Tbx1+/+	47	0.108556491	0.651052972	0.901457961	FALSE
down_fm	P8	PdgfraCre;Tbx1+/+	WT;Tbx1+/flox	29	-0.078765755	0.798445998	0.974374778	FALSE
		PdgfraCre;Tbx1+/+	WT;Tbx1+/+	30	-0.265207331	0.272287052	0.901457961	FALSE
		WT;Tbx1+/flox	WT;Tbx1+/+	37	-0.132603665	0.599878278	0.901457961	FALSE
	P12	PdgfraCre;Tbx1+/+	WT;Tbx1+/flox	21.5	0.395447343	0.11349239	0.74285928	FALSE
		PdgfraCre;Tbx1+/+	WT;Tbx1+/+	37.5	0.255883158	0.277317729	0.901457961	FALSE
		WT;Tbx1+/flox	WT;Tbx1+/+	38.5	-0.240375088	0.310026197	0.901457961	FALSE
up_fm	P8	PdgfraCre;Tbx1+/+	WT;Tbx1+/flox	22	-0.262552516	0.328205128	0.901457961	FALSE
		PdgfraCre;Tbx1+/+	WT;Tbx1+/+	36	-0.151547046	0.544759334	0.901457961	FALSE
		WT;Tbx1+/flox	WT;Tbx1+/+	38.5	0.104188594	0.71678442	0.955712559	FALSE
	P12	PdgfraCre;Tbx1+/+	WT;Tbx1+/flox	22	-0.385040834	0.11349239	0.74285928	FALSE
		PdgfraCre;Tbx1+/+	WT;Tbx1+/+	35	-0.294653333	0.193005137	0.901457961	FALSE
		WT;Tbx1+/flox	WT;Tbx1+/+	41.5	0.193850877	0.422107304	0.901457961	FALSE
two_steps	P8	PdgfraCre;Tbx1+/+	WT;Tbx1+/flox	22.5	-0.24942489	0.382284382	0.901457961	FALSE
		PdgfraCre;Tbx1+/+	WT;Tbx1+/+	16.5	-0.520942971	0.0259321	0.701799748	FALSE
		WT;Tbx1+/flox	WT;Tbx1+/+	32.5	-0.217848879	0.395041147	0.901457961	FALSE
	P12	PdgfraCre;Tbx1+/+	WT;Tbx1+/flox	26	-0.301788762	0.222418758	0.901457961	FALSE
		PdgfraCre;Tbx1+/+	WT;Tbx1+/+	38	-0.248129123	0.277317729	0.901457961	FALSE
		WT;Tbx1+/flox	WT;Tbx1+/+	50.5	0.054278246	0.8620692	1	FALSE
flat	P8	PdgfraCre;Tbx1+/+	WT;Tbx1+/flox	13	0.49884978	0.04988345	0.701799748	FALSE
		PdgfraCre;Tbx1+/+	WT;Tbx1+/+	22.5	0.407282686	0.090841735	0.74285928	FALSE
		WT;Tbx1+/flox	WT;Tbx1+/+	23	-0.397810996	0.090841735	0.74285928	FALSE
	P12	PdgfraCre;Tbx1+/+	WT;Tbx1+/flox	29	0.239349707	0.340106952	0.901457961	FALSE
		PdgfraCre;Tbx1+/+	WT;Tbx1+/+	39	-0.232621053	0.310026197	0.901457961	FALSE
		WT;Tbx1+/flox	WT;Tbx1+/+	26.5	-0.42647193	0.058483312	0.701799748	FALSE
chevron	P8	PdgfraCre;Tbx1+/+	WT;Tbx1+/flox	29	0.078765755	0.798445998	0.974374778	FALSE
		PdgfraCre;Tbx1+/+	WT;Tbx1+/+	36	-0.151547046	0.544759334	0.901457961	FALSE
		WT;Tbx1+/flox	WT;Tbx1+/+	32	-0.227320569	0.351062422	0.901457961	FALSE
	P12	PdgfraCre;Tbx1+/+	WT;Tbx1+/flox	37	0.072845563	0.796174414	0.974374778	FALSE
		PdgfraCre;Tbx1+/+	WT;Tbx1+/+	52	-0.03101614	0.917007451	1	FALSE
		WT;Tbx1+/flox	WT;Tbx1+/+	48.5	-0.085294386	0.754370088	0.974374778	FALSE
complex	P8	PdgfraCre;Tbx1+/+	WT;Tbx1+/flox	26	-0.157531509	0.573737374	0.901457961	FALSE
		PdgfraCre;Tbx1+/+	WT;Tbx1+/+	35	-0.170490427	0.49202191	0.901457961	FALSE
		WT;Tbx1+/flox	WT;Tbx1+/+	44	0	1	1	FALSE
	P12	PdgfraCre;Tbx1+/+	WT;Tbx1+/flox	34	-0.135284617	0.604812834	0.901457961	FALSE
		PdgfraCre;Tbx1+/+	WT;Tbx1+/+	44	-0.155080702	0.507876025	0.901457961	FALSE
		WT;Tbx1+/flox	WT;Tbx1+/+	46	0.124064561	0.60159902	0.901457961	FALSE
step_up	P8	PdgfraCre;Tbx1+/+	WT;Tbx1+/flox	31	0.026255252	0.959129759	1	FALSE
		PdgfraCre;Tbx1+/+	WT;Tbx1+/+	43.5	-0.00947169	1	1	FALSE
		WT;Tbx1+/flox	WT;Tbx1+/+	40	0.075773523	0.777989468	0.974374778	FALSE
	P12	PdgfraCre;Tbx1+/+	WT;Tbx1+/flox	28	0.260162725	0.297326203	0.901457961	FALSE
		PdgfraCre;Tbx1+/+	WT;Tbx1+/+	46	-0.124064561	0.60159902	0.901457961	FALSE
		WT;Tbx1+/flox	WT;Tbx1+/+	24	-0.465242105	0.033865206	0.701799748	FALSE
mult_steps	P8	PdgfraCre;Tbx1+/+	WT;Tbx1+/flox	32	0	1	1	FALSE
		PdgfraCre;Tbx1+/+	WT;Tbx1+/+	36	-0.151547046	0.544759334	0.901457961	FALSE
		WT;Tbx1+/flox	WT;Tbx1+/+	36	-0.151547046	0.544759334	0.901457961	FALSE
	P12	PdgfraCre;Tbx1+/+	WT;Tbx1+/flox	40.5	0	1	1	FALSE
		PdgfraCre;Tbx1+/+	WT;Tbx1+/+	45	-0.139572632	0.553791719	0.901457961	FALSE
		WT;Tbx1+/flox	WT;Tbx1+/+	45	-0.139572632	0.553791719	0.901457961	FALSE
rev_chevron	P8	PdgfraCre;Tbx1+/+	WT;Tbx1+/flox	27	-0.131276258	0.645376845	0.901457961	FALSE
		PdgfraCre;Tbx1+/+	WT;Tbx1+/+	44	0	1	1	FALSE
		WT;Tbx1+/flox	WT;Tbx1+/+	37	0.132603665	0.599878278	0.901457961	FALSE
	P12	PdgfraCre;Tbx1+/+	WT;Tbx1+/flox	33	-0.156097635	0.545701357	0.901457961	FALSE
		PdgfraCre;Tbx1+/+	WT;Tbx1+/+	40	-0.217112982	0.345102575	0.901457961	FALSE
		WT;Tbx1+/flox	WT;Tbx1+/+	47	-0.108556491	0.651052972	0.901457961	FALSE

Figure	Measurement	Call type	PND	Genotype	# of Samples	Average	s.e.m	Shapiro-Wilk test Statistic	p_value	p_adj(FDR BH)	Rejects(FDR 5%)	Levene test Statistic	df1	df2	p_value	p_adj(FDR BH)	Rejects(FDR 5%)
Figure 4C	Duration	Harmonics	P8	oTbx1+/+	23	47.92295829	2.699256034	0.979313314	0.8941468	0.989708006	FALSE	0.000929168	1	31	0.975877848	0.985623162	FALSE
				oTbx1+/-	10	44.69960544	4.401566176	0.974668384	0.930436969	0.989708006	FALSE						
			P12	oTbx1+/+	22	54.74005891	3.852574293	0.918354988	0.070288621	0.331429934	FALSE	1.537197377	1	29	0.224974682	0.490853852	FALSE
				oTbx1+/-	9	52.60944711	3.167958994	0.916101158	0.360939831	0.770815088	FALSE						
		step_down	P8	oTbx1+/+	18	27.09201646	3.053456892	0.865561366	0.015047655	0.158000372	FALSE	0.2059045	1	23	0.654249229	0.962256678	FALSE
				oTbx1+/-	7	20.10412698	3.302056133	0.904883921	0.361587048	0.770815088	FALSE						
			P12	oTbx1+/+	15	27.33660106	1.7963783	0.982755959	0.98487395	0.989708006	FALSE	2.068498506	1	20	0.165834425	0.429742989	FALSE
				oTbx1+/-	7	28.09701587	8.430810214	0.715816438	0.005502878	0.077620809	FALSE						
		short	P8	oTbx1+/+	27	7.74224882	0.312538479	0.945187688	0.163532123	0.528334551	FALSE	0.171090279	1	36	0.68159848	0.962256678	FALSE
				oTbx1+/-	11	7.774337147	0.612486858	0.846332788	0.038221501	0.229329005	FALSE						
			P12	oTbx1+/+	26	7.072236643	0.334744164	0.87922591	0.005544343	0.077620809	FALSE	0.00038636	1	34	0.984432657	0.985623162	FALSE
				oTbx1+/-	10	7.017617959	0.485436046	0.96597141	0.851189494	0.989708006	FALSE						
		down_fm	P8	oTbx1+/+	24	30.66279906	1.957850728	0.966806293	0.589142263	0.907498758	FALSE	3.431740525	1	33	0.072916956	0.35000139	FALSE
				oTbx1+/-	11	30.69520938	1.801882739	0.955081701	0.709225118	0.930857968	FALSE						
			P12	oTbx1+/+	25	35.67096042	2.959919864	0.969138086	0.62329185	0.907498758	FALSE	2.160735587	1	32	0.151341379	0.429742989	FALSE
				oTbx1+/-	9	37.35198732	3.365400362	0.94418776	0.626606286	0.907498758	FALSE						
		up_fm	P8	oTbx1+/+	23	19.62332101	0.925405003	0.95598197	0.387208849	0.770815088	FALSE	5.35177982	1	30	0.027737942	0.221903537	FALSE
				oTbx1+/-	9	23.60520165	3.653717179	0.921376705	0.403760284	0.770815088	FALSE						
			P12	oTbx1+/+	24	19.72581213	1.368821557	0.947432995	0.238222212	0.667022192	FALSE	0.058377287	1	32	0.810620958	0.985623162	FALSE
				oTbx1+/-	10	15.11864466	1.853801309	0.986202121	0.989708006	0.989708006	FALSE						
		two_steps	P8	oTbx1+/+	12	56.71407407	8.656626098	0.946003079	0.579481781	0.907498758	FALSE	2.000905298	1	14	0.179059579	0.429742989	FALSE
				oTbx1+/-	4	50.34666667	5.410990188	0.792466283	0.089381523	0.341274908	FALSE						
			P12	oTbx1+/+	10	58.62755556	4.194056899	0.951295912	0.683848679	0.926504662	FALSE	0.000336509	1	14	0.985623162	0.985623162	FALSE
				oTbx1+/-	6	34.70222222	5.58446381	0.918257356	0.492899448	0.840512896	FALSE						
		flat	P8	oTbx1+/+	26	19.78944829	0.81484035	0.984566271	0.953157961	0.989708006	FALSE	1.174282732	1	34	0.286148033	0.572296066	FALSE
				oTbx1+/-	10	21.47446293	2.726545128	0.82753104	0.031245172	0.218716204	FALSE						
			P12	oTbx1+/+	24	22.07510842	0.90491284	0.950715065	0.280761451	0.736998808	FALSE	2.759012943	1	31	0.106790325	0.4271613	FALSE
				oTbx1+/-	9	21.11847067	0.935953023	0.891232073	0.205424145	0.616272435	FALSE						
		chevron	P8	oTbx1+/+	21	36.64371988	2.271811058	0.919187725	0.0835924	0.341274908	FALSE	0.003024766	1	27	0.956545111	0.985623162	FALSE
				oTbx1+/-	8	37.73476406	3.772893681	0.954048574	0.751886845	0.956946893	FALSE						
			P12	oTbx1+/+	20	41.88753169	2.667991417	0.975014746	0.855094075	0.989708006	FALSE	0.204884413	1	25	0.654710508	0.962256678	FALSE
				oTbx1+/-	7	37.00915927	3.607040424	0.965664983	0.865602672	0.989708006	FALSE						
		complex	P8	oTbx1+/+	12	53.79841981	3.593523806	0.928657532	0.366100401	0.770815088	FALSE	0.004785745	1	12	0.945986497	0.985623162	FALSE
				oTbx1+/-	2	47.81714286	8.92952381										
			P12	oTbx1+/+	15	62.12476912	6.394717172	0.939049602	0.370569885	0.770815088	FALSE	0.257959301	1	21	0.616818976	0.962256678	FALSE
				oTbx1+/-	8	53.32536201	7.106570063	0.86379683	0.130967751	0.458387129	FALSE						
		step_up	P8	oTbx1+/+	21	39.52578272	2.436556474	0.955946565	0.43852523	0.800785202	FALSE	2.443787387	1	28	0.129224079	0.429742989	FALSE
				oTbx1+/-	9	50.31356252	6.763535213	0.971306562	0.905666947	0.989708006	FALSE						
			P12	oTbx1+/+	19	36.25782364	3.631192924	0.956213474	0.500305295	0.840512896	FALSE	0.000525987	1	25	0.981884673	0.985623162	FALSE
				oTbx1+/-	8	54.54926984	8.836116188	0.641596675	0.000484111	0.020332659	TRUE						
		mult_steps	P8	oTbx1+/+	2	45.01333333	37.33333333					1.84044E+31	1	1	1.48395E-16	1.78074E-15	TRUE
				oTbx1+/-	1	48.21333333											
			P12	oTbx1+/+	2	13.01333333	5.76					2.80384E+31	1	1	1.20227E-16	1.78074E-15	TRUE
				oTbx1+/-	1	52.90666667											
		rev_chevron	P8	oTbx1+/+	12	42.56651852	2.153044286	0.841997564	0.029282464	0.218716204	FALSE	4.749155614	1	12	0.049960362	0.299762171	FALSE
				oTbx1+/-	2	38.4	14.50666667										
			P12	oTbx1+/+	9	47.50971016	5.892589066	0.94786489	0.666641116	0.926504662	FALSE	0.537574475	1	15	0.474743246	0.87644907	FALSE
				oTbx1+/-	8	37.21680672	5.066803973	0.837518036	0.0710207	0.331429934	FALSE						

Linear Mixed Model

Source	F	df1	df2	Sig.
Corrected Model	20.26123774	47	560	0
Genotype	0.043473793	1	560	0.834911507
PND	0.165222066	1	560	0.684548787
Call type	60.43094494	11	560	0
Genotype * PND	0.105188211	1	560	0.745811343
PND * Call type	1.398862882	11	560	0.169112377
Call type * Genotype	3.360831125	11	560	0.000163775
Genotype * PND * Call type	1.038937083	11	560	0.410076829

Mann-Witney U test

Sample1	Sample2	Statistic	R (Z score)	p_value	p_adj(FDR BH)	Rejects(FDR 5%)
oTbx1+/+	oTbx1+/-	129	-0.101817612	0.602683656	0.911355311	FALSE
oTbx1+/+	oTbx1+/-	97	-0.356581323	0.948931589	0.990189484	FALSE
oTbx1+/+	oTbx1+/-	86	-0.326338501	0.177507801	0.710031204	FALSE
oTbx1+/+	oTbx1+/-	68	-0.481169978	0.298245614	0.911355311	FALSE
oTbx1+/+	oTbx1+/-	132	0.086153364	0.611651952	0.911355311	FALSE
oTbx1+/+	oTbx1+/-	133	-0.201919544	0.930816808	0.990189484	FALSE
oTbx1+/+	oTbx1+/-	137	-0.060046284	0.874932414	0.990189484	FALSE
oTbx1+/+	oTbx1+/-	101	-0.339396681	0.673085541	0.911355311	FALSE
oTbx1+/+	oTbx1+/-	91	-0.300231421	0.62111142	0.911355311	FALSE
oTbx1+/+	oTbx1+/-	165	-0.064442408	0.092918255	0.557509532	FALSE
oTbx1+/+	oTbx1+/-	28	-0.629180629	0.683516484	0.911355311	FALSE
oTbx1+/+	oTbx1+/-	53	-0.545612386	0.010989011	0.263736264	FALSE
oTbx1+/+	oTbx1+/-	113	-0.185360268	0.565958548	0.911355311	FALSE
oTbx1+/+	oTbx1+/-	129	-0.219104186	0.414164975	0.911355311	FALSE
oTbx1+/+	oTbx1+/-	81	-0.352445581	0.904925626	0.990189484	FALSE
oTbx1+/+	oTbx1+/-	87	-0.399542928	0.369771292	0.911355311	FALSE
oTbx1+/+	oTbx1+/-	16	-0.691837621	0.549450549	0.911355311	FALSE
oTbx1+/+	oTbx1+/-	68	-0.481169978	0.635523359	0.911355311	FALSE
oTbx1+/+	oTbx1+/-	52	-0.503866645	0.056277595	0.557509532	FALSE
oTbx1+/+	oTbx1+/-	42	-0.592870152	0.074893866	0.557509532	FALSE
oTbx1+/+	oTbx1+/-	1	-0.770158862	1	1	FALSE
oTbx1+/+	oTbx1+/-	0	-0.773308893	0.666666667	0.911355311	FALSE
oTbx1+/+	oTbx1+/-	13	-0.707501869	0.923076923	0.990189484	FALSE
oTbx1+/+	oTbx1+/-	52	-0.549908546	0.138790621	0.666194981	FALSE

Figure	Measurement	Call type	PND	Genotype	# of Samples	Average	s.e.m	Shapiro-Wilk test Statistic	p_value	p_adj(FDR BH)	Rejects(FDR 5%)	Levene test Statistic	df1	df2	p_value	p_adj(FDR BH)	Rejects(FDR 5%)
Figure 4C	Duration	All	P8	oTbx1+/+	27	260.2677275	23.10002451	0.966685951	0.517069697	0.689426263	FALSE	0.024977278	1	36	0.875306878	0.875306878	FALSE
				oTbx1+/-	11	237.7105327	35.22954873	0.98531574	0.988519132	0.988519132	FALSE						
			P12	oTbx1+/+	27	266.3561112	29.14567445	0.965607464	0.490966678	0.689426263	FALSE	0.933710604	1	36	0.34034749	0.68069498	FALSE
				oTbx1+/-	11	281.6978642	39.45653524	0.85749799	0.053434357	0.213737428	FALSE						

Repeated 2-way ANOVA

Source		Type III Sum of Squares	df	Mean Square	F	Sig.
**Within subjects**						
PND	Sphericity Assumed	11487.37597	1	11487.37597	0.749626853	0.393039589
PND * Genotype	Sphericity Assumed	5543.314971	1	5543.314971	0.361737769	0.551780088
Error(PND)	Sphericity Assumed	490372.0159	32	15324.1255		
**Between Subjects**						
Intercept		3819990.943	1	3819990.943	166.4686802	3.16998E-14
Genotype		5.440297392	1	5.440297392	0.000237079	0.987810766
Error		734310.5625	32	22947.20508		

Student t test

Sample1	Sample2	Statistic	dof	cohens_d	p_value	p_adj(FDR BH)	Rejects(FDR 5%)
oTbx1+/+	oTbx1+/-	0.529251423	36	0.189310895	0.599879665	0.770649486	FALSE
oTbx1+/+	oTbx1+/-	-0.293732063	36	-0.105066661	0.770649486	0.770649486	FALSE

Measurement	Call type	PND	Genotype	# of samples	Average	s.e.m	Shapiro-Wilk test Statistic	p_value	p_adj(FDR BH)	Rejects(FDR 5%)	Levene's test Statistic	df1	df2	p_value	p_adj(FDR BH)	Rejects(FDR 5%)
Duration	Harmonics	P8	PdgfraCre;Tbx1+/+	7	40.90886772	4.558427742	0.938468978	0.624970931	0.989653678	FALSE	0.002206996	2	20	0.997795681	0.997795681	FALSE
			WT;Tbx1+/flox	5	42.93581128	5.625100406	0.880300822	0.310676402	0.861420933	FALSE						
			WT;Tbx1+/+	11	54.65335546	3.322269435	0.906698369	0.2227774	0.747542576	FALSE						
		P12	PdgfraCre;Tbx1+/+	7	47.1113757	5.674386478	0.953771049	0.763849974	0.989653678	FALSE	2.463344678	2	19	0.111885654	0.830248368	FALSE
			WT;Tbx1+/flox	6	64.71990508	11.31009545	0.964217505	0.851580079	0.989653678	FALSE						
			WT;Tbx1+/+	9	54.02024841	3.022231296	0.965456458	0.853325402	0.989653678	FALSE						
	step_down	P8	PdgfraCre;Tbx1+/+	5	26.92266667	3.372016607	0.877170705	0.296686073	0.861420933	FALSE	0.430873811	2	15	0.657736097	0.996273433	FALSE
			WT;Tbx1+/flox	5	23.83644444	4.227757401	0.930576706	0.60030857	0.989653678	FALSE						
			WT;Tbx1+/+	8	29.23259259	6.236899193	0.834931578	0.066786083	0.493875425	FALSE						
		P12	PdgfraCre;Tbx1+/+	2	27.97511111	1.379555556					1.403826013	2	12	0.283250233	0.890215017	FALSE
			WT;Tbx1+/flox	5	27.04863492	4.662940802	0.93123362	0.604806917	0.989653678	FALSE						
			WT;Tbx1+/+	8	27.35695238	2.060533643	0.942444012	0.635276389	0.989653678	FALSE						
	short	P8	PdgfraCre;Tbx1+/+	8	7.917815937	0.530604626	0.831355391	0.061324236	0.493875425	FALSE	0.02965329	2	24	0.970817565	0.997795681	FALSE
			WT;Tbx1+/flox	8	7.81333203	0.768281296	0.655720075	0.00070415	0.042953152	TRUE						
			WT;Tbx1+/+	11	7.562866763	0.416805441	0.985154377	0.988045907	0.989653678	FALSE						
		P12	PdgfraCre;Tbx1+/+	8	7.224727273	0.302944896	0.892752792	0.248184669	0.756963239	FALSE	0.62103357	2	23	0.54615906	0.996273433	FALSE
			WT;Tbx1+/flox	7	6.733424679	0.691876485	0.83817731	0.095540452	0.58279676	FALSE						
			WT;Tbx1+/+	11	7.176941981	0.650036053	0.89527921	0.161741928	0.686425775	FALSE						
	down_fm	P8	PdgfraCre;Tbx1+/+	7	31.22903484	2.618699941	0.966327518	0.870816941	0.989653678	FALSE	2.420919834	2	21	0.113215687	0.830248368	FALSE
			WT;Tbx1+/flox	6	24.00876305	5.033714095	0.858242658	0.183218167	0.698519264	FALSE						
			WT;Tbx1+/+	11	33.93194139	2.490502409	0.977074081	0.947711079	0.989653678	FALSE						
		P12	PdgfraCre;Tbx1+/+	8	35.27615686	5.281399559	0.962996667	0.838114522	0.989653678	FALSE	0.086686621	2	22	0.917276034	0.997795681	FALSE
			WT;Tbx1+/flox	6	40.01672804	7.675950561	0.963961751	0.849691462	0.989653678	FALSE						
			WT;Tbx1+/+	11	33.58767159	4.005392441	0.958638001	0.754588316	0.989653678	FALSE						
	up_fm	P8	PdgfraCre;Tbx1+/+	6	22.08635809	1.767895755	0.979031784	0.94664488	0.989653678	FALSE	0.803986224	2	20	0.46148733	0.996273433	FALSE
			WT;Tbx1+/flox	7	17.68497536	1.231492248	0.778875725	0.025161975	0.383720116	FALSE						
			WT;Tbx1+/+	10	19.50234072	1.538468802	0.88695579	0.156663332	0.686425775	FALSE						
		P12	PdgfraCre;Tbx1+/+	7	18.25207174	1.882487619	0.881423279	0.23284113	0.747542576	FALSE	0.393935778	2	21	0.679277341	0.996273433	FALSE
			WT;Tbx1+/flox	7	19.37050535	2.449587654	0.952865475	0.755690423	0.989653678	FALSE						
			WT;Tbx1+/+	10	21.00614516	2.58398818	0.900295495	0.220738544	0.747542576	FALSE						
	two_steps	P8	PdgfraCre;Tbx1+/+	1	56.74666667						0.756333907	2	9	0.497030518	0.996273433	FALSE
			WT;Tbx1+/flox	3	39.37185185	14.56837035	0.765693459	0.034986854	0.426839615	FALSE						
			WT;Tbx1+/+	8	63.21333333	11.47831855	0.944422712	0.655048847	0.989653678	FALSE						
		P12	PdgfraCre;Tbx1+/+	1	59.59111111						1.718803649	2	7	0.247024372	0.890215017	FALSE
			WT;Tbx1+/flox	4	63.89333333	5.261381314	0.948534927	0.706977093	0.989653678	FALSE						
			WT;Tbx1+/+	5	54.22222222	7.218198127	0.955559829	0.776776713	0.989653678	FALSE						
	flat	P8	PdgfraCre;Tbx1+/+	8	19.38285418	2.161523708	0.938127232	0.592752901	0.989653678	FALSE	1.88909965	2	23	0.173933952	0.890215017	FALSE
			WT;Tbx1+/flox	7	20.03418318	1.026599508	0.918007443	0.45407321	0.989653678	FALSE						
			WT;Tbx1+/+	11	19.92941271	1.060503268	0.930035527	0.411213037	0.989653678	FALSE						
		P12	PdgfraCre;Tbx1+/+	7	22.06660313	2.099649318	0.855242162	0.137169031	0.686425775	FALSE	0.661867473	2	21	0.526320871	0.996273433	FALSE
			WT;Tbx1+/flox	6	23.32211358	1.547504437	0.833187707	0.114362185	0.6341903	FALSE						
			WT;Tbx1+/+	11	21.40033624	1.281598243	0.985002754	0.987590136	0.989653678	FALSE						
	chevron	P8	PdgfraCre;Tbx1+/+	6	36.00005604	4.652989959	0.795458709	0.053475915	0.493875425	FALSE	0.059069292	2	18	0.942823397	0.997795681	FALSE
			WT;Tbx1+/flox	6	32.45211329	4.474684324	0.990198852	0.989653678	0.989653678	FALSE						
			WT;Tbx1+/+	9	39.86723351	3.153606108	0.913333661	0.33990915	0.901498181	FALSE						
		P12	PdgfraCre;Tbx1+/+	5	35.15140741	5.45098299	0.794380663	0.072866866	0.493875425	FALSE	0.724169595	2	17	0.499090145	0.996273433	FALSE
			WT;Tbx1+/flox	6	43.83518788	3.031502235	0.981537083	0.958938137	0.989653678	FALSE						
			WT;Tbx1+/+	9	44.33138549	4.612113275	0.930351025	0.484695498	0.989653678	FALSE						
	complex	P8	PdgfraCre;Tbx1+/+	2	52.06552381	5.47352381					4.243778405	2	9	0.050328058	0.830248368	FALSE
			WT;Tbx1+/flox	4	50.53333333	2.727266981	0.983042133	0.919621758	0.989653678	FALSE						
			WT;Tbx1+/+	6	56.55277613	6.979082855	0.944235993	0.693480606	0.989653678	FALSE						
		P12	PdgfraCre;Tbx1+/+	3	56.86289128	15.15889488	0.98798584	0.790240544	0.989653678	FALSE	0.007045782	2	12	0.992983086	0.997795681	FALSE
			WT;Tbx1+/flox	5	68.77497258	12.75070208	0.964095962	0.836176977	0.989653678	FALSE						
			WT;Tbx1+/+	7	59.62971429	9.27012584	0.944156119	0.676391711	0.989653678	FALSE						
	step_up	P8	PdgfraCre;Tbx1+/+	5	40.9686811	3.783911882	0.84138729	0.168793223	0.686425775	FALSE	1.646688998	2	18	0.220420241	0.890215017	FALSE
			WT;Tbx1+/flox	6	43.3817284	3.877075903	0.690721619	0.00492818	0.150309498	FALSE						
			WT;Tbx1+/+	10	36.49076612	4.16921906	0.943592325	0.593632891	0.989653678	FALSE						
		P12	PdgfraCre;Tbx1+/+	5	32.81066667	8.360663706	0.944022431	0.694498426	0.989653678	FALSE	0.168774818	2	16	0.8461834	0.997795681	FALSE
			WT;Tbx1+/flox	4	28.91555556	8.444243272	0.92068423	0.540789471	0.989653678	FALSE						
			WT;Tbx1+/+	10	40.91830937	4.426603923	0.969189671	0.883231963	0.989653678	FALSE						
	mult_steps	P8	PdgfraCre;Tbx1+/+	0												
			WT;Tbx1+/flox	0												
			WT;Tbx1+/+	2	45.01333333	37.33333333										
		P12	PdgfraCre;Tbx1+/+	0												
			WT;Tbx1+/flox	0												
			WT;Tbx1+/+	2	13.01333333	5.76										
	rev_chevron	P8	PdgfraCre;Tbx1+/+	3	43.94666667	3.205526093	0.998031496	0.91523587	0.989653678	FALSE	0.132365162	2	9	0.877695297	0.997795681	FALSE
			WT;Tbx1+/flox	4	41.984	2.412836781	0.991194337	0.963506274	0.989653678	FALSE						
			WT;Tbx1+/+	5	42.20444444	4.861369009	0.690556819	0.007608686	0.154709958	FALSE						
		P12	PdgfraCre;Tbx1+/+	1	43.6224						0.468689127	2	6	0.646945397	0.996273433	FALSE
			WT;Tbx1+/flox	3	58.64296296	12.13379604	0.982868766	0.749306217	0.989653678	FALSE						
			WT;Tbx1+/+	5	41.60722051	7.264631898	0.944978233	0.701312904	0.989653678	FALSE						

Generalized Linear Mixed Model

Source	F	df1	df2	Sig.
Corrected Model	11.02882805	67	364	0
Genotype	0.347190073	2	364	0.706904756
PND	1.596530724	1	364	0.207204201
Call type	52.1277172	11	364	0
Genotype * PND	5.022025307	2	364	0.007055228
PND * Call type	1.821393368	11	364	0.048996774
Call type * Genotype	0.536675492	20	364	0.950317556
Genotype * PND * Call type	0.998274051	20	364	0.463368195

Student's t-test

Sample1	Sample2	Statistic	dof	Cohen's d	p_value	p_adj(FDR BH)	Rejects(FDR 5%)
PdgfraCre;Tbx1+/+	WT;Tbx1+/flox	-0.282121004	10	-0.165193145	0.783603353	0.968298081	FALSE
PdgfraCre;Tbx1+/+	WT;Tbx1+/+	-2.489154851	16	-1.203490884	0.024196155	0.968298081	FALSE
WT;Tbx1+/flox	WT;Tbx1+/+	-1.891444267	14	-1.020169172	0.079436704	0.968298081	FALSE
PdgfraCre;Tbx1+/+	WT;Tbx1+/flox	-1.457113075	11	-0.810662878	0.173032593	0.968298081	FALSE
PdgfraCre;Tbx1+/+	WT;Tbx1+/+	-1.144156066	14	-0.576600459	0.271744981	0.968298081	FALSE
WT;Tbx1+/flox	WT;Tbx1+/+	1.091739122	13	0.57539704	0.294768343	0.968298081	FALSE
PdgfraCre;Tbx1+/+	WT;Tbx1+/flox	0.570697045	8	0.360940503	0.583874953	0.968298081	FALSE
PdgfraCre;Tbx1+/+	WT;Tbx1+/+	-0.273985489	11	-0.15619576	0.789172445	0.968298081	FALSE
WT;Tbx1+/flox	WT;Tbx1+/+	-0.623418251	11	-0.355403084	0.545719109	0.968298081	FALSE
PdgfraCre;Tbx1+/+	WT;Tbx1+/flox	0.118223257	5	0.098912673	0.910493384	0.968298081	FALSE
PdgfraCre;Tbx1+/+	WT;Tbx1+/+	0.142292666	8	0.11249223	0.890367177	0.968298081	FALSE
WT;Tbx1+/flox	WT;Tbx1+/+	-0.069161795	11	-0.039428289	0.94610209	0.968298081	FALSE
PdgfraCre;Tbx1+/+	WT;Tbx1+/flox	0.11190297	14	0.055951485	0.912488936	0.968298081	FALSE
PdgfraCre;Tbx1+/+	WT;Tbx1+/+	0.533322905	17	0.247813921	0.600713923	0.968298081	FALSE
WT;Tbx1+/flox	WT;Tbx1+/+	0.307715843	17	0.142983302	0.762036619	0.968298081	FALSE
PdgfraCre;Tbx1+/+	WT;Tbx1+/flox	0.681216815	13	0.352563197	0.507688111	0.968298081	FALSE
PdgfraCre;Tbx1+/+	WT;Tbx1+/+	0.059016841	17	0.027422777	0.953626898	0.968298081	FALSE
WT;Tbx1+/flox	WT;Tbx1+/+	-0.449666572	16	-0.21741099	0.658982876	0.968298081	FALSE
PdgfraCre;Tbx1+/+	WT;Tbx1+/flox	1.329497727	11	0.739664253	0.210596869	0.968298081	FALSE
PdgfraCre;Tbx1+/+	WT;Tbx1+/+	-0.717870319	16	-0.347085833	0.483188151	0.968298081	FALSE
WT;Tbx1+/flox	WT;Tbx1+/+	-1.993865557	15	-1.01192509	0.064675183	0.968298081	FALSE
PdgfraCre;Tbx1+/+	WT;Tbx1+/flox	-0.526963161	12	-0.284592634	0.607823392	0.968298081	FALSE
PdgfraCre;Tbx1+/+	WT;Tbx1+/+	0.25976169	17	0.120700916	0.79816736	0.968298081	FALSE
WT;Tbx1+/flox	WT;Tbx1+/+	0.825483127	15	0.418948552	0.422032461	0.968298081	FALSE
PdgfraCre;Tbx1+/+	WT;Tbx1+/flox	2.090998344	11	1.163324085	0.060536036	0.968298081	FALSE
PdgfraCre;Tbx1+/+	WT;Tbx1+/+	1.06895429	14	0.552005622	0.303175501	0.968298081	FALSE
WT;Tbx1+/flox	WT;Tbx1+/+	-0.858608983	15	-0.423127126	0.404068462	0.968298081	FALSE
PdgfraCre;Tbx1+/+	WT;Tbx1+/flox	-0.362025824	12	-0.193510942	0.723627738	0.968298081	FALSE
PdgfraCre;Tbx1+/+	WT;Tbx1+/+	-0.790464576	15	-0.389545196	0.441575344	0.968298081	FALSE
WT;Tbx1+/flox	WT;Tbx1+/+	-0.440145385	15	-0.216906014	0.666107981	0.968298081	FALSE
PdgfraCre;Tbx1+/+	WT;Tbx1+/flox	0.596319781	2		0.6114663	0.968298081	FALSE
PdgfraCre;Tbx1+/+	WT;Tbx1+/+	-0.187793669	7		0.856366123	0.968298081	FALSE
WT;Tbx1+/flox	WT;Tbx1+/+	-1.135840723	9	-0.768967804	0.285363816	0.968298081	FALSE
PdgfraCre;Tbx1+/+	WT;Tbx1+/flox	-0.365685768	3		0.738863479	0.968298081	FALSE
PdgfraCre;Tbx1+/+	WT;Tbx1+/+	0.303654689	4		0.776530465	0.968298081	FALSE
WT;Tbx1+/flox	WT;Tbx1+/+	1.028937284	7	0.690232114	0.337744317	0.968298081	FALSE
PdgfraCre;Tbx1+/+	WT;Tbx1+/flox	-0.259433573	13	-0.13426963	0.799363748	0.968298081	FALSE
PdgfraCre;Tbx1+/+	WT;Tbx1+/+	-0.247055629	17	-0.114796915	0.807824052	0.968298081	FALSE
WT;Tbx1+/flox	WT;Tbx1+/+	0.066877881	16	0.032335039	0.947507498	0.968298081	FALSE
PdgfraCre;Tbx1+/+	WT;Tbx1+/flox	-0.466876518	11	-0.259746116	0.649699228	0.968298081	FALSE
PdgfraCre;Tbx1+/+	WT;Tbx1+/+	0.288188199	16	0.139337201	0.776902466	0.968298081	FALSE
WT;Tbx1+/flox	WT;Tbx1+/+	0.922891212	15	0.468385027	0.370671424	0.968298081	FALSE
PdgfraCre;Tbx1+/+	WT;Tbx1+/flox	0.549602398	10	0.317313092	0.594652508	0.968298081	FALSE
PdgfraCre;Tbx1+/+	WT;Tbx1+/+	-0.715913679	13	-0.377319639	0.486706	0.968298081	FALSE
WT;Tbx1+/flox	WT;Tbx1+/+	-1.397947944	13	-0.736783259	0.185525854	0.968298081	FALSE
PdgfraCre;Tbx1+/+	WT;Tbx1+/flox	-1.458624868	9	-0.88324122	0.178666411	0.968298081	FALSE
PdgfraCre;Tbx1+/+	WT;Tbx1+/+	-1.236549181	12	-0.68971418	0.239909947	0.968298081	FALSE
WT;Tbx1+/flox	WT;Tbx1+/+	-0.079848715	13	-0.042083968	0.937573691	0.968298081	FALSE
PdgfraCre;Tbx1+/+	WT;Tbx1+/flox	0.289710654	4	0.250896786	0.786434528	0.968298081	FALSE
PdgfraCre;Tbx1+/+	WT;Tbx1+/+	-0.345156593	6	-0.281819178	0.741754886	0.968298081	FALSE
WT;Tbx1+/flox	WT;Tbx1+/+	-0.669843422	8	-0.43238207	0.52181832	0.968298081	FALSE
PdgfraCre;Tbx1+/+	WT;Tbx1+/flox	-0.587160054	6	-0.428801075	0.578505405	0.968298081	FALSE
PdgfraCre;Tbx1+/+	WT;Tbx1+/+	-0.160572581	8	-0.110805608	0.876410673	0.968298081	FALSE
WT;Tbx1+/flox	WT;Tbx1+/+	0.596278589	10	0.349144991	0.564242133	0.968298081	FALSE
PdgfraCre;Tbx1+/+	WT;Tbx1+/flox	-0.440276927	9	-0.266600919	0.670120851	0.968298081	FALSE
PdgfraCre;Tbx1+/+	WT;Tbx1+/+	0.685188585	13	0.375293244	0.505259597	0.968298081	FALSE
WT;Tbx1+/flox	WT;Tbx1+/+	1.112199041	14	0.574337115	0.28478682	0.968298081	FALSE
PdgfraCre;Tbx1+/+	WT;Tbx1+/flox	0.323606166	7	0.217081616	0.755689769	0.968298081	FALSE
PdgfraCre;Tbx1+/+	WT;Tbx1+/+	-0.949195702	13	-0.519895898	0.35983407	0.968298081	FALSE
WT;Tbx1+/flox	WT;Tbx1+/+	-1.373262061	12	-0.812432792	0.194785619	0.968298081	FALSE
PdgfraCre;Tbx1+/+	WT;Tbx1+/flox						
PdgfraCre;Tbx1+/+	WT;Tbx1+/+						
WT;Tbx1+/flox	WT;Tbx1+/+						
PdgfraCre;Tbx1+/+	WT;Tbx1+/flox						
PdgfraCre;Tbx1+/+	WT;Tbx1+/+						
WT;Tbx1+/flox	WT;Tbx1+/+						
PdgfraCre;Tbx1+/+	WT;Tbx1+/flox	0.501057483	5	0.382688974	0.637605545	0.968298081	FALSE
PdgfraCre;Tbx1+/+	WT;Tbx1+/+	0.252803413	6	0.184621509	0.808858345	0.968298081	FALSE
WT;Tbx1+/flox	WT;Tbx1+/+	-0.037327957	7	-0.025040355	0.971265737	0.971265737	FALSE
PdgfraCre;Tbx1+/+	WT;Tbx1+/flox	-0.618955639	2		0.599052199	0.968298081	FALSE
PdgfraCre;Tbx1+/+	WT;Tbx1+/+	0.113246424	4		0.915291352	0.968298081	FALSE
WT;Tbx1+/flox	WT;Tbx1+/+	1.297665641	6	0.947680991	0.242055899	0.968298081	FALSE

Measurement	Call type	PND	Genotype	# of samples	Average	s.e.m	Shapiro-Wilk test Statistic	p_value	p_adj(FDR BH)	Rejects(FDR 5%)	Levene's test Statistic	df1	df2	p_value	p_adj(FDR BH)	Rejects(FDR 5%)
Duration	All	P8	PdgfraCre;Tbx1+/+	8	213.0079521	37.53124163	0.926702066	0.486557768	0.862529525	FALSE	0.211873328	2	24	0.810564284	0.810564284	FALSE
			WT;Tbx1+/flox	8	218.4553202	44.93729257	0.967337109	0.876331943	0.961358291	FALSE						
			WT;Tbx1+/+	11	325.0474966	30.83900383	0.924511428	0.358022468	0.862529525	FALSE						
		P12	PdgfraCre;Tbx1+/+	9	180.1755783	41.40608696	0.939344896	0.575019684	0.862529525	FALSE	0.390880507	2	24	0.680689304	0.810564284	FALSE
			WT;Tbx1+/flox	7	320.0533714	68.70128939	0.980379231	0.961358291	0.961358291	FALSE						
			WT;Tbx1+/+	11	302.6964725	39.66885402	0.929910487	0.409948701	0.862529525	FALSE						

Repeated Measures 2 Way ANOVA

Source	Correction	Type III Sum of Squares	df	Mean Square	F	Sig.
**Within Subjects**						
PND	Sphericity Assumed	3902.027524	1	3902.027524	0.312510909	0.582056653
PND * Genotype	Sphericity Assumed	40899.39978	2	20449.69989	1.637803489	0.218281319
Error(PND)	Sphericity Assumed	262207.0967	21	12486.05222		
**Between Subjects**						
Intercept		3049614.801	1	3049614.801	129.5812445	1.91413E-10
Genotype		120624.2088	2	60312.1044	2.562722855	0.100956354
Error		494222.0692	21	23534.38425		

Student's t-test

Sample1	Sample2	Statistic	dof	Cohen's d	p_value	p_adj(FDR BH)	Rejects(FDR 5%)
PdgfraCre;Tbx1+/+	WT;Tbx1+/flox	-0.093039895	14	-0.046519947	0.92719021	0.92719021	FALSE
PdgfraCre;Tbx1+/+	WT;Tbx1+/+	-2.320848973	17	-1.078406121	0.032983261	0.117249306	FALSE
WT;Tbx1+/flox	WT;Tbx1+/+	-2.027150892	17	-0.941936316	0.058624653	0.117249306	FALSE
PdgfraCre;Tbx1+/+	WT;Tbx1+/flox	-1.831107564	14	-0.922791474	0.088446699	0.132670049	FALSE
PdgfraCre;Tbx1+/+	WT;Tbx1+/+	-2.123771635	18	-0.954564363	0.047806477	0.117249306	FALSE
WT;Tbx1+/flox	WT;Tbx1+/+	0.235645859	16	0.113933307	0.816697222	0.92719021	FALSE

								Shapiro-Wilk test				Levene test						Linear Mixed Model					Mann-Whitney U test						
Figure	Measurement	Month	Session	Genotype	# of samples	Average	s.e.m	Statistic	p_value	p_adj(FDR BH)	Rejects(FDR 5%)	Statistic	df1	df2	p_value	p_adj(FDR BH)	Rejects(FDR 5%)	Source	F	df1	df2	Sig.	Sample1	Sample2	Statistic	R (Z score)	p_value	p_adj(FDR BH)	Rejects(FDR 5%)
Figure 5A	Social Interaction (s)	1	1	oTbx1+/+	27	16.40740741	3.092707745	0.822716916	0.000347817	0.000695635	TRUE	0.0722396	1	36	0.789637762	0.789637762	FALSE	Corrected Model	0.497244222	3	72	0.685351082	oTbx1+/-	oTbx1+/+	138.5	-0.05221416	0.775387862	0.849168654	FALSE
				oTbx1+/-	11	16.09090909	6.185693972	0.732690896	0.001237266	0.001649687	TRUE							Genotype	0.111765542	1	72	0.739114848							
			2	oTbx1+/+	27	14.40740741	3.858708012	0.644196044	7.09586E-07	2.83834E-06	TRUE	0.344949992	1	36	0.56065175	0.789637762	FALSE	Session	1.379597591	1	72	0.244039836	oTbx1+/-	oTbx1+/+	142	-0.033939204	0.849168654	0.849168654	FALSE
				oTbx1+/-	11	11	3.311138228	0.855904878	0.050946602	0.050946602	FALSE							Genotype * Session	0.262132613	1	72	0.610225677							
		2	1	oTbx1+/+	21	24.23809524	7.075411737	0.65323431	7.59541E-06	1.51908E-05	TRUE	0.093406833	1	29	0.762073072	0.762073072	FALSE	Corrected Model	1.489687533	3	58	0.226836385	oTbx1+/-	oTbx1+/+	93.5	0.087281656	0.662541772	0.723978187	FALSE
				oTbx1+/-	10	30.3	16.01808006	0.521146224	6.44244E-06	1.51908E-05	TRUE							Genotype	0.005469838	1	58	0.941298006							
			2	oTbx1+/+	21	19.61904762	5.080061956	0.764962029	0.000200312	0.000267082	TRUE	0.24965746	1	29	0.621086413	0.762073072	FALSE	Session	4.309691377	1	58	0.042338451	oTbx1+/-	oTbx1+/+	95.5	-0.072102238	0.723978187	0.723978187	FALSE
				oTbx1+/-	10	15.2	5.133333333	0.841080498	0.045458931	0.045458931	TRUE							Genotype * Session	1.217519133	1	58	0.274405205							

							Shapiro-Wilk test				Levene test						Linear Mixed Model					Mann-Whitney U test						
Measurement	Month	Session	Genotype	# of samples	Average	s.e.m	Statistic	p_value	p_adj(FDR BH)	Rejects(FDR 5%)	Statistic	df1	df2	p_value	p_adj(FDR BH)	Rejects(FDR 5%)	Source	F	df1	df2	Sig.	Sample1	Sample2	Statistic	R (Z score)	p_value	p_adj(FDR BH)	Rejects(FDR 5%)
Social Interaction (s)	1	1	PdgfraCre;Tbx1+/+	8	19	6.450359901	0.852384539	0.100707919	0.151061878	FALSE	1.333074967	2	24	0.282495218	0.282495218	FALSE	Corrected Model	1.081487416	5	48	0.382584793	PdgfraCre;Tbx1+/+	WT;Tbx1+/flox	25.5	0.245049015	0.370382559	0.745047522	FALSE
			WT;Tbx1+/+	10	20	6.151783842	0.853382418	0.063721582	0.151061878	FALSE							Genotype	1.686430667	2	48	0.195965476	PdgfraCre;Tbx1+/+	WT;Tbx1+/+	37	0.062828086	0.828557064	0.828557064	FALSE
			WT;Tbx1+/flox	9	10.11111111	2.468942894	0.870970279	0.125930494	0.151116593	FALSE							Session	0.495285736	1	48	0.484979032	WT;Tbx1+/flox	WT;Tbx1+/+	35.5	-0.177951304	0.496698348	0.745047522	FALSE
		2	PdgfraCre;Tbx1+/+	8	11.625	4.37907973	0.852137342	0.100130923	0.151061878	FALSE	1.941945267	2	24	0.165308228	0.282495218	FALSE	Genotype*Session	0.843209591	2	48	0.436601196	PdgfraCre;Tbx1+/+	WT;Tbx1+/flox	31	0.116690007	0.672974085	0.807568902	FALSE
			WT;Tbx1+/+	10	23.3	9.316711389	0.746606164	0.003244757	0.01946854	TRUE												PdgfraCre;Tbx1+/+	WT;Tbx1+/+	29.5	-0.219898302	0.408245349	0.745047522	FALSE
			WT;Tbx1+/flox	9	7	1.748014747	0.925306341	0.437971545	0.437971545	FALSE												WT;Tbx1+/flox	WT;Tbx1+/+	29	-0.29970746	0.21102427	0.745047522	FALSE
	2	1	PdgfraCre;Tbx1+/+	8	25.375	10.82480204	0.646705991	0.000554496	0.001663489	TRUE	0.105027705	2	18	0.900847215	0.972131	FALSE	Corrected Model	0.499425251	5	36	0.774566604	PdgfraCre;Tbx1+/+	WT;Tbx1+/flox	27	0.029880715	0.955089355	0.955089355	FALSE
			WT;Tbx1+/+	6	26.16666667	19.80642432	0.554416353	0.000124267	0.000745601	TRUE							Genotype	0.03924997	2	36	0.961551418	PdgfraCre;Tbx1+/+	WT;Tbx1+/+	13.5	0.362284419	0.228438228	0.89044289	FALSE
			WT;Tbx1+/flox	7	21.28571429	7.870119155	0.815067345	0.057543741	0.067157791	FALSE							Session	1.75456665	1	36	0.193651322	WT;Tbx1+/flox	WT;Tbx1+/+	14.5	0.257539377	0.445221445	0.89044289	FALSE
		2	PdgfraCre;Tbx1+/+	8	17.875	6.418270517	0.835164751	0.067157791	0.067157791	FALSE	0.028309139	2	18	0.972131	0.972131	FALSE	Genotype*Session	0.238208993	2	36	0.789270203	PdgfraCre;Tbx1+/+	WT;Tbx1+/flox	26	0.05976143	0.866511267	0.955089355	FALSE
			WT;Tbx1+/+	6	22.83333333	12.78910126	0.642868731	0.00146069	0.002921381	TRUE												PdgfraCre;Tbx1+/+	WT;Tbx1+/+	20.5	-0.120761473	0.754578755	0.955089355	FALSE
			WT;Tbx1+/flox	7	18.85714286	9.174053919	0.752239945	0.013389431	0.020084147	TRUE												WT;Tbx1+/flox	WT;Tbx1+/+	15	-0.237728656	0.445221445	0.89044289	FALSE

								Shapiro-Wilk test				Levene test						Linear Mixed Model					Mann-Whitney U test						
Figure	Measurement	Month	Session	Genotype	# of samples	Average	s.e.m	Statistic	p_value	p_adj(FDR BH)	Rejects(FDR 5%)	Statistic	df1	df2	p_value	p_adj(FDR BH)	Rejects(FDR 5%)	Source	F	df1	df2	Sig.	Sample1	Sample2	Statistic	R (Z score)	p_value	p_adj(FDR BH)	Rejects(FDR 5%)
Figure 5B	Sniffing novel object (s)	1	1	oTbx1+/+	26	14.57692308	2.634781017	0.846840904	0.001236135	0.00247227	TRUE	0.08242971	1	35	0.775724316	0.775724316	FALSE	Corrected Model	2.521664315	3	70	0.064800787	oTbx1+/-	oTbx1+/+	129	-0.076479138	0.65844134	0.934817788	FALSE
				oTbx1+/-	11	12.36363636	3.739889953	0.818474025	0.016467715	0.016467715	TRUE							Genotype	2.25065E-05	1	70	0.996228266							
			2	oTbx1+/+	26	8.115384615	1.778786901	0.802681024	0.000195641	0.000782565	TRUE	1.057934255	1	35	0.310742865	0.621485731	FALSE	Session	3.706544227	1	70	0.05826308	oTbx1+/-	oTbx1+/+	140	-0.016388387	0.934817788	0.934817788	FALSE
				oTbx1+/-	11	10.36363636	4.101602186	0.789282155	0.006799356	0.009065808	TRUE							Genotype * Session	1.03048056	1	70	0.31354175							
		2	1	oTbx1+/+	21	8	1.657594356	0.868273317	0.008915895	0.01783179	TRUE	2.542177104	1	29	0.12168399	0.243367981	FALSE	Corrected Model	0.495546864	3	58	0.686790912	oTbx1+/-	oTbx1+/+	104	0.007589709	0.983438982	0.983438982	FALSE
				oTbx1+/-	10	6.8	1.41264134	0.911900064	0.294299164	0.294299164	FALSE							Genotype	0.116338641	1	58	0.734272506							
			2	oTbx1+/+	21	6.333333333	1.731134136	0.786296484	0.000408019	0.001632078	TRUE	0.028910762	1	29	0.86616589	0.86616589	FALSE	Session	0.920506319	1	58	0.341324669	oTbx1+/-	oTbx1+/+	99	0.045538256	0.81932528	0.983438982	FALSE
				oTbx1+/-	10	5.9	2.008039398	0.823579501	0.027996164	0.037328218	TRUE							Genotype * Session	0.082129844	1	58	0.775452844							

							Shapiro-Wilk test				Levene test						Generalized Linear Mixed Model					Mann-Whitney U test						
Measurement	Month	Session	Genotype	# of samples	Average	s.e.m	Statistic	p_value	p_adj(FDR BH)	Rejects(FDR 5%)	Statistic	df1	df2	p_value	p_adj(FDR BH)	Rejects(FDR 5%)	Source	F	df1	df2	Sig.	Sample1	Sample2	Statistic	R (Z score)	p_value	p_adj(FDR BH)	Rejects(FDR 5%)
Sniffing novel object (s)	1	1	PdgfraCre;Tbx1+/+	8	12	3.9551052	0.861423719	0.124057733	0.148869279	FALSE	0.476633313	2	23	0.626867139	0.952913718	FALSE	Corrected Model	3.093385391	5	46	0.017260974	PdgfraCre;Tbx1+/+	WT;Tbx1+/flox	30.5	-0.128359008	0.672974085	0.931427396	FALSE
			WT;Tbx1+/+	9	14	3.989569735	0.818691778	0.033304831	0.066609661	FALSE							Genotype	0.177160277	2	46	0.838214318	PdgfraCre;Tbx1+/+	WT;Tbx1+/+	30	-0.140028008	0.605841218	0.931427396	FALSE
			WT;Tbx1+/flox	9	17.44444444	5.725457554	0.864838833	0.10819449	0.148869279	FALSE							Session	12.98794535	1	46	0.000767292	WT;Tbx1+/flox	WT;Tbx1+/+	37	0.072845563	0.796174414	0.931427396	FALSE
		2	PdgfraCre;Tbx1+/+	8	7	3.5152728	0.759035249	0.010275376	0.030826129	TRUE	0.048332199	2	23	0.952913718	0.952913718	FALSE	Genotype*Session	0.931759073	2	46	0.401165818	PdgfraCre;Tbx1+/+	WT;Tbx1+/flox	28	-0.186704011	0.480707528	0.931427396	FALSE
			WT;Tbx1+/+	9	9.555555556	3.667087518	0.721553597	0.00253196	0.01519176	TRUE												PdgfraCre;Tbx1+/+	WT;Tbx1+/+	26	-0.233380014	0.370382559	0.931427396	FALSE
			WT;Tbx1+/flox	9	7.666666667	2.254624876	0.919315229	0.386600395	0.386600395	FALSE												WT;Tbx1+/flox	WT;Tbx1+/+	39	-0.031219527	0.931427396	0.931427396	FALSE
	2	1	PdgfraCre;Tbx1+/+	8	7.5	2.692582404	0.907626652	0.337653424	0.37778911	FALSE	0.627763682	2	18	0.545071894	0.545071894	FALSE	Corrected Model	0.477274668	5	36	0.790748408	PdgfraCre;Tbx1+/+	WT;Tbx1+/flox	27	-0.029880715	0.955089355	1	FALSE
			WT;Tbx1+/+	6	8	2.695675549	0.87228892	0.23548219	0.353223285	FALSE							Genotype	0.057987693	2	36	0.943749519	PdgfraCre;Tbx1+/+	WT;Tbx1+/+	23.5	-0.017251639	1	1	FALSE
			WT;Tbx1+/flox	7	8.571428571	3.537938214	0.813275039	0.055283937	0.110567873	FALSE							Session	1.061158579	1	36	0.309822637	WT;Tbx1+/flox	WT;Tbx1+/+	19	-0.079242885	0.835664336	1	FALSE
		2	PdgfraCre;Tbx1+/+	8	8	4.110092109	0.764055086	0.011666808	0.070000849	FALSE	1.298312466	2	18	0.297363734	0.545071894	FALSE	Genotype*Session	0.658142803	2	36	0.523930478	PdgfraCre;Tbx1+/+	WT;Tbx1+/flox	27	0.029880715	0.955089355	1	FALSE
			WT;Tbx1+/+	6	6.333333333	1.452966315	0.90065047	0.37778911	0.37778911	FALSE												PdgfraCre;Tbx1+/+	WT;Tbx1+/+	19.5	-0.155264751	0.662004662	1	FALSE
			WT;Tbx1+/flox	7	4.428571429	2.191511143	0.796592563	0.037898712	0.110567873	FALSE												WT;Tbx1+/flox	WT;Tbx1+/+	14	-0.277350098	0.365967366	1	FALSE

Figure	Measurement	Month	Delay (s)	Genotype	# of samples	Average	s.e.m	Shapiro-Wilk test<br>Statistic	p_value	p_adj(FDR BH)	Rejects(FDR 5%)	Levene test<br>Statistic	df1	df2	p_value	p_adj(FDR BH)	Rejects(FDR 5%)	Linear Mixed Model<br>Source	F	df1	df2	Sig.	Mann-Whitney U test<br>Sample1	Sample2	Statistic	R (Z score)	p_value	p_adj(FDR BH)	Rejects(FDR 5%)
Figure 6A	% alternation	1	0	oTbx1+/+	24	87.96296296	2.209774585	0.817882433	0.000586058	0.001758173	TRUE	1.26349718	1	32	0.269351926	0.808055777	FALSE	Corrected Model	66.06011981	5	96	0	oTbx1+/-	oTbx1+/+	113	0.045374261	0.809114032	0.809114032	FALSE
				oTbx1+/-	10	88.88888889	3.3126933	0.84073699	0.04503041	0.054036492	FALSE							Genotype	4.660889678	1	96	0.033349839							
			15	oTbx1+/+	24	51.85185185	2.184325276	0.813035203	0.000484908	0.001758173	TRUE	0.003101076	1	32	0.955937164	0.955937164	FALSE	Delay	122.9828701	2	96	0	oTbx1+/-	oTbx1+/+	113	0.045374261	0.809114032	0.809114032	FALSE
				oTbx1+/-	10	53.33333333	3.228814032	0.884986306	0.14879822	0.14879822	FALSE							Genotype * Delay	3.150937861	2	96	0.04727228							
			30	oTbx1+/+	24	44.90740741	2.059150213	0.905249105	0.027853786	0.041780679	TRUE	0.011740247	1	32	0.914392707	0.955937164	FALSE						oTbx1+/-	oTbx1+/+	43	0.499116867	0.002700336	0.008101007	TRUE
				oTbx1+/-	10	57.77777778	2.771598064	0.819704545	0.025134415	0.041780679	TRUE																		
		2	0	oTbx1+/+	20	88.88888889	2.418254167	0.832136365	0.002714676	0.008144029	TRUE	0.005608641	1	27	0.940853676	0.940853676	FALSE	Corrected Model	38.5469499	5	81	0	oTbx1+/-	oTbx1+/+	80	-0.087537622	0.660118692	0.660118692	FALSE
				oTbx1+/-	9	85.18518519	4.899539465	0.790885567	0.016062009	0.032124018	TRUE							Genotype	2.046863951	1	81	0.156366686							
			15	oTbx1+/+	20	54.44444444	2.265657383	0.898171934	0.038114561	0.057171842	FALSE	2.474961012	1	27	0.127318695	0.381956086	FALSE	Delay	81.56087251	2	81	0	oTbx1+/-	oTbx1+/+	72.5	-0.153190838	0.443859589	0.660118692	FALSE
				oTbx1+/-	9	48.14814815	5.237828009	0.911520399	0.326661423	0.337937343	FALSE							Genotype * Delay	0.243946056	2	81	0.784103458							
			30	oTbx1+/+	20	51.11111111	2.47140896	0.800994201	0.000893986	0.005363913	TRUE	0.039618489	1	27	0.843719703	0.940853676	FALSE						oTbx1+/-	oTbx1+/+	75	-0.131306433	0.501389665	0.660118692	FALSE
				oTbx1+/-	9	49.38271605	3.266359643	0.913067364	0.337937343	0.337937343	FALSE																		

Measurement	Month	Delay (s)	Genotype	# of samples	Average	s.e.m	Shapiro-Wilk test<br>Statistic	p_value	p_adj(FDR BH)	Rejects(FDR 5%)	Levene test<br>Statistic	df1	df2	p_value	p_adj(FDR BH)	Rejects(FDR 5%)	Generalized Linear Mixed Model<br>Source	F	df1	df2	Sig.	Mann-Whitney U test<br>Sample1	Sample2	Statistic	R (Z score)	p_value	p_adj(FDR BH)	Rejects(FDR 5%)
% alternation	1	0	PdgfraCre;Tbx1+/+	8	86.11111111	2.777777778	0.827205306	0.05551859	0.124916827	FALSE	0.400924437	2	21	0.674718601	0.961718489	FALSE	Corrected Model	31.01103484	8	63	0	PdgfraCre;Tbx1+/+	WT;Tbx1+/flox	26.5	-0.221711013	0.423447141	1	FALSE
			WT;Tbx1+/+	7	87.3015873	4.489566865	0.664435629	0.001497441	0.006738486	TRUE							Genotype	0.104620037	2	63	0.900822824	PdgfraCre;Tbx1+/+	WT;Tbx1+/+	27.5	-0.014940358	1	1	FALSE
			WT;Tbx1+/flox	9	90.12345679	4.320987654	0.775541227	0.01070126	0.032103779	TRUE							Delay	121.2150635	2	63	0	WT;Tbx1+/flox	WT;Tbx1+/+	27.5	0.105851225	0.757692308	1	FALSE
		15	PdgfraCre;Tbx1+/+	8	50	4.199605256	0.567903737	6.66981E-05	0.000600283	TRUE	0.122464503	2	21	0.885364514	0.961718489	FALSE	Genotype*Delay	0.26479949	4	63	0.899485819	PdgfraCre;Tbx1+/+	WT;Tbx1+/flox	35	-0.023338001	0.962566845	1	FALSE
			WT;Tbx1+/+	7	53.96825397	4.489566865	0.893581377	0.293870591	0.293870591	FALSE												PdgfraCre;Tbx1+/+	WT;Tbx1+/+	22.5	-0.164343934	0.612587413	1	FALSE
			WT;Tbx1+/flox	9	51.85185185	3.207501495	0.872821722	0.131797654	0.175446148	FALSE												WT;Tbx1+/flox	WT;Tbx1+/+	27	-0.119082628	0.680594406	1	FALSE
		30	PdgfraCre;Tbx1+/+	8	45.83333333	3.893138268	0.871515193	0.155952131	0.175446148	FALSE	0.039106145	2	21	0.961718489	0.961718489	FALSE						PdgfraCre;Tbx1+/+	WT;Tbx1+/flox	35.5	-0.011669001	1	1	FALSE
			WT;Tbx1+/+	7	44.44444444	3.428963332	0.8577129	0.144397137	0.175446148	FALSE												PdgfraCre;Tbx1+/+	WT;Tbx1+/+	27	0.029880715	0.955089355	1	FALSE
			WT;Tbx1+/flox	9	44.44444444	3.703703704	0.853289618	0.080990849	0.145783528	FALSE												WT;Tbx1+/flox	WT;Tbx1+/+	30	0.039694209	0.918181818	1	FALSE
	2	0	PdgfraCre;Tbx1+/+	8	87.5	3.893138268	0.735991362	0.005710635	0.019119856	TRUE	0.205732484	2	17	0.816047931	0.925960593	FALSE	Corrected Model	22.23991673	8	51	3.94129E-14	PdgfraCre;Tbx1+/+	WT;Tbx1+/flox	22	-0.069006556	0.851814852	0.94971695	FALSE
			WT;Tbx1+/+	6	90.74074074	5.30289669	0.772718964	0.032937266	0.074108848	FALSE							Genotype	0.119696984	2	51	0.887437723	PdgfraCre;Tbx1+/+	WT;Tbx1+/+	19.5	-0.155264751	0.662004662	0.94971695	FALSE
			WT;Tbx1+/flox	6	88.88888889	4.05720413	0.853191607	0.167002833	0.214717928	FALSE							Delay	87.4546486	2	51	0	WT;Tbx1+/flox	WT;Tbx1+/+	15	-0.138675049	0.699134199	0.94971695	FALSE
		15	PdgfraCre;Tbx1+/+	8	55.55555556	2.969569355	0.848910992	0.092877818	0.139316727	FALSE	0.476	2	17	0.629298498	0.925960593	FALSE	Genotype*Delay	0.640087093	4	51	0.636335206	PdgfraCre;Tbx1+/+	WT;Tbx1+/flox	17	0.241522946	0.413586414	0.94971695	FALSE
			WT;Tbx1+/+	6	57.40740741	5.30289669	0.907578534	0.420658529	0.473240845	FALSE												PdgfraCre;Tbx1+/+	WT;Tbx1+/+	23	-0.034503278	0.94971695	0.94971695	FALSE
			WT;Tbx1+/flox	6	50	3.79516695	0.701259348	0.006373285	0.019119856	TRUE												WT;Tbx1+/flox	WT;Tbx1+/+	13	-0.231125082	0.484848485	0.94971695	FALSE
		30	PdgfraCre;Tbx1+/+	8	52.77777778	3.482128725	0.826041799	0.053987435	0.097177383	FALSE	0.077272727	2	17	0.925960593	0.925960593	FALSE						PdgfraCre;Tbx1+/+	WT;Tbx1+/flox	23	0.034503278	0.94971695	0.94971695	FALSE
			WT;Tbx1+/+	6	48.14814815	5.493480361	0.665788341	0.002641354	0.019119856	TRUE												PdgfraCre;Tbx1+/+	WT;Tbx1+/+	19.5	0.155264751	0.662004662	0.94971695	FALSE
			WT;Tbx1+/flox	6	51.85185185	4.684855793	0.915458969	0.473270921	0.473270921	FALSE												WT;Tbx1+/flox	WT;Tbx1+/+	15.5	0.115562541	0.818181818	0.94971695	FALSE

Figure	Measurement	Month	Delay (s)	Genotype	# of samples	Average	s.e.m	Shapiro-Wilk test				Levene test						Linear Mixed Model					Mann-Whitney U test						
								Statistic	p_value	p_adj(FDR BH)	Rejects(FDR 5%)	Statistic	df1	df2	p_value	p_adj(FDR BH)	Rejects(FDR 5%)	Source	F	df1	df2	Sig.	Sample1	Sample2	Statistic	R (Z score)	p_value	p_adj(FDR BH)	Rejects(FDR 5%)
Figure 6B	Latency to correct arm (s)	1	0	oTbx1+/+	24	7.060929233	0.514093404	0.800175638	0.00029653	0.00177918	TRUE	0.277615077	1	32	0.601902435	0.601902435	FALSE	Corrected Model	16.83605715	5	96	6.44695E-12	oTbx1+/-	oTbx1+/+	110	0.064820372	0.723944044	0.896531195	FALSE
				oTbx1+/-	10	7.045238095	0.530928951	0.926337453	0.412848339	0.476660672	FALSE							Genotype	2.181306251	1	96	0.142968861							
			15	oTbx1+/+	24	23.71111111	3.024286701	0.822986774	0.000717253	0.00215176	TRUE	0.326055759	1	32	0.571979595	0.601902435	FALSE	Delay	27.42527721	2	96	3.79112E-10	oTbx1+/-	oTbx1+/+	116	-0.025928149	0.896531195	0.896531195	FALSE
				oTbx1+/-	10	21.89666667	3.777162368	0.850065677	0.058193748	0.116387496	FALSE							Genotype * Delay	2.058495873	2	96	0.13324324							
			30	oTbx1+/+	24	30.76111111	2.763625603	0.951467639	0.29143235	0.437148525	FALSE	1.123976658	1	32	0.297000607	0.601902435	FALSE						oTbx1+/-	oTbx1+/+	67	-0.343547973	0.045949085	0.137847254	FALSE
				oTbx1+/-	10	20.47833333	3.235213323	0.932865391	0.476660672	0.476660672	FALSE																		
		2	0	oTbx1+/+	20	8.249603175	1.084890689	0.650063636	1.01826E-05	6.10959E-05	TRUE	1.416496538	1	27	0.244340874	0.366511312	FALSE	Corrected Model	9.617022113	5	81	3.11418E-07	oTbx1+/-	oTbx1+/+	79	0.096291384	0.626843222	0.626843222	FALSE
				oTbx1+/-	9	10.25432099	2.448449218	0.825388972	0.039632942	0.118898826	FALSE							Genotype	1.113528338	1	81	0.294453611							
			15	oTbx1+/+	20	21.45690476	2.262232887	0.949405569	0.358157001	0.358157001	FALSE	1.772805112	1	27	0.194168243	0.366511312	FALSE	Delay	18.44615477	2	81	2.50312E-07	oTbx1+/-	oTbx1+/+	58	0.28012039	0.13969359	0.419080769	FALSE
				oTbx1+/-	9	32.12037037	6.192265298	0.86589138	0.11106244	0.145532979	FALSE							Genotype * Delay	2.582518592	2	81	0.081796465							
			30	oTbx1+/+	20	27.41833333	3.194822863	0.924555501	0.121277482	0.145532979	FALSE	0.387060947	1	27	0.539067153	0.539067153	FALSE						oTbx1+/-	oTbx1+/+	71	-0.166321482	0.390153575	0.585230362	FALSE
				oTbx1+/-	9	23.54814815	4.343311557	0.852295522	0.078981247	0.145532979	FALSE																		

Measurement	Month	Delay (s)	Genotype	# of samples	Average	s.e.m	Shapiro-Wilk test				Levene test						Generalized Linear Mixed Model					Mann-Whitney U test						
							Statistic	p_value	p_adj(FDR BH)	Rejects(FDR 5%)	Statistic	df1	df2	p_value	p_adj(FDR BH)	Rejects(FDR 5%)	Source	F	df1	df2	Sig.	Sample1	Sample2	Statistic	R (Z score)	p_value	p_adj(FDR BH)	Rejects(FDR 5%)
Latency to correct arm (s)	1	0	PdgfraCre;Tbx1+/+	8	6.936507937	1.245302549	0.49801402	9.77951E-06	8.80156E-05	TRUE	0.118489741	2	21	0.888850552	0.888850552	FALSE	Corrected Model	12.80465967	8	63	9.75433E-11	PdgfraCre;Tbx1+/+	WT;Tbx1+/flox	26	-0.233380014	0.370382559	0.666688606	FALSE
			WT;Tbx1+/+	7	7.238095238	0.859979629	0.889504742	0.272090107	0.61220274	FALSE							Genotype	1.578749645	2	63	0.214282282	PdgfraCre;Tbx1+/+	WT;Tbx1+/+	27	-0.029880715	0.955089355	1	FALSE
			WT;Tbx1+/flox	9	7.033730159	0.619516622	0.953498416	0.728441817	0.964816546	FALSE							Delay	41.38453208	2	63	3.34022E-12	WT;Tbx1+/flox	WT;Tbx1+/+	31.5	0	1	1	FALSE
		15	PdgfraCre;Tbx1+/+	8	32.51875	7.148675811	0.867888501	0.143716676	0.61220274	FALSE	1.806979696	2	21	0.188754388	0.283131582	FALSE	Genotype*Delay	4.562533663	4	63	0.0026726	PdgfraCre;Tbx1+/+	WT;Tbx1+/flox	30	0.140028008	0.605841218	0.778938708	FALSE
			WT;Tbx1+/+	7	13.89285714	1.511419745	0.881281719	0.232198499	0.61220274	FALSE												PdgfraCre;Tbx1+/+	WT;Tbx1+/+	8	0.597614305	0.020512821	0.140034965	FALSE
			WT;Tbx1+/flox	9	23.51851852	3.323532402	0.936177907	0.542290058	0.964816546	FALSE												WT;Tbx1+/flox	WT;Tbx1+/+	14	0.463099109	0.071153846	0.213461538	FALSE
		30	PdgfraCre;Tbx1+/+	8	33.4	5.751512051	0.954254508	0.753939808	0.964816546	FALSE	2.91193178	2	21	0.076529454	0.229588362	FALSE						PdgfraCre;Tbx1+/+	WT;Tbx1+/flox	20	0.373408022	0.138790621	0.312278898	FALSE
			WT;Tbx1+/+	7	37.25952381	4.89605426	0.981124342	0.964816546	0.964816546	FALSE												PdgfraCre;Tbx1+/+	WT;Tbx1+/+	22	-0.179284291	0.535819736	0.778938708	FALSE
			WT;Tbx1+/flox	9	23.36111111	2.668082668	0.967019227	0.868103653	0.964816546	FALSE												WT;Tbx1+/flox	WT;Tbx1+/+	11	-0.542487527	0.031118881	0.140034965	FALSE
	2	0	PdgfraCre;Tbx1+/+	8	7.634672619	0.856014268	0.893506903	0.252187548	0.567421983	FALSE	2.318063852	2	17	0.128762611	0.193143916	FALSE	Corrected Model	5.716167127	8	51	3.39007E-05	PdgfraCre;Tbx1+/+	WT;Tbx1+/flox	20	-0.138013112	0.662004662	0.754578755	FALSE
			WT;Tbx1+/+	6	6.136574074	0.419288306	0.935332466	0.621863247	0.799538461	FALSE							Genotype	0.163060535	2	51	0.849980901	PdgfraCre;Tbx1+/+	WT;Tbx1+/+	14.5	0.327781141	0.282384282	0.754578755	FALSE
			WT;Tbx1+/flox	6	11.18253968	3.263533536	0.781374891	0.039698931	0.357290375	FALSE							Delay	20.11273606	2	51	3.63144E-07	WT;Tbx1+/flox	WT;Tbx1+/+	12.5	0.25423759	0.484848485	0.754578755	FALSE
		15	PdgfraCre;Tbx1+/+	8	18.46458333	3.639106975	0.925920356	0.479712667	0.799538461	FALSE	3.251300612	2	17	0.063728018	0.191184054	FALSE	Genotype*Delay	1.061101788	4	51	0.385349838	PdgfraCre;Tbx1+/+	WT;Tbx1+/flox	16	-0.276026224	0.344988345	0.754578755	FALSE
			WT;Tbx1+/+	6	20.77301587	2.072925902	0.928919732	0.571785689	0.799538461	FALSE												PdgfraCre;Tbx1+/+	WT;Tbx1+/+	18	-0.207019668	0.490842491	0.754578755	FALSE
			WT;Tbx1+/flox	6	26.13055556	5.381154386	0.960050918	0.820132956	0.820132956	FALSE												WT;Tbx1+/flox	WT;Tbx1+/+	15	0.138675049	0.699134199	0.754578755	FALSE
		30	PdgfraCre;Tbx1+/+	8	28.00625	6.083776841	0.851422944	0.0984807	0.44316315	FALSE	0.906736253	2	17	0.422502853	0.422502853	FALSE						PdgfraCre;Tbx1+/+	WT;Tbx1+/flox	21	0.103509834	0.754578755	0.754578755	FALSE
			WT;Tbx1+/+	6	30.63333333	6.500957194	0.873464834	0.240368777	0.567421983	FALSE												PdgfraCre;Tbx1+/+	WT;Tbx1+/+	21	-0.103509834	0.754578755	0.754578755	FALSE
			WT;Tbx1+/flox	6	23.41944444	3.546480824	0.954883247	0.779566398	0.820132956	FALSE												WT;Tbx1+/flox	WT;Tbx1+/+	13	-0.231125082	0.484848485	0.754578755	FALSE

							Shapiro-Wilk test				Levene test						Mann-Whitney U test						
Figure	Measurement	Month	Genotype	# of samples	Average	s.e.m	Statistic	p_value	p_adj(FDR BH)	Rejects(FDR 5%)	Statistic	df1	df2	p_value	p_adj(FDR BH)	Rejects(FDR 5%)	Sample1	Sample2	Statistic	R (Z score)	p_value	p_adj(FDR BH)	Rejects(FDR 5%)
Figure 7A	% open arms time	1	oTbx1+/+	23	13.92453346	2.578767506	0.871431687	0.00682799	0.01365598	TRUE	0.03061532	1	32	0.862203787	0.862203787	FALSE	oTbx1+/-	oTbx1+/+	100	0.167302625	0.343976779	0.343976779	FALSE
			oTbx1+/-	11	16.98607089	3.279767612	0.906204785	0.219762803	0.219762803	FALSE													
		2	oTbx1+/+	20	5.617823791	1.293061822	0.865730567	0.009893527	0.019787054	TRUE	0.129376135	1	28	0.721778502	0.721778502	FALSE	oTbx1+/-	oTbx1+/+	98	0.016064387	0.948316451	0.948316451	FALSE
			oTbx1+/-	10	6.108311403	1.697112495	0.861318188	0.079087694	0.079087694	FALSE													

						Shapiro-Wilk test				Levene test						One way ANOVA						Student t test							
Measurement	Month	Genotype	# of samples	Average	s.e.m	Statistic	p_value	p_adj(FDR BH)	Rejects(FDR 5%)	Statistic	df1	df2	p_value	p_adj(FDR BH)	Rejects(FDR 5%)	Source	Type III Sum of Squares	df	Mean Square	F	Sig.	Sample1	Sample2	Statistic	dof	cohens_d	p_value	p_adj(FDR BH)	Rejects(FDR 5%)
% open arms time	1	PdgfraCre;Tbx1+/+	7	12.90725421	4.103497891	0.886610333	0.257444394	0.386166591	FALSE	0.107045624	2	20	0.898995957	0.898995957	FALSE	Intercept	4421.166501	1	4421.166501	26.47361987	4.92643E-05	PdgfraCre;Tbx1+/+	WT;Tbx1+/flox	-0.090770003	14	-0.045743782	0.928961264	0.928961264	FALSE
		WT;Tbx1+/+	7	15.44729701	4.180055074	0.973508997	0.922513345	0.922513345	FALSE							Genotype	24.86679522	2	12.43339761	0.074450271	0.928509658	PdgfraCre;Tbx1+/+	WT;Tbx1+/+	-0.43363151	12	-0.231785792	0.672249124	0.928961264	FALSE
		WT;Tbx1+/flox	9	13.53137901	5.135617804	0.770175921	0.009280317	0.02784095	TRUE							Error	3340.054381	20	167.0027191			WT;Tbx1+/flox	WT;Tbx1+/+	-0.277229778	14	-0.139710676	0.785655127	0.928961264	FALSE
	2	PdgfraCre;Tbx1+/+	8	7.962568057	2.541302393	0.9223745	0.449425688	0.674138532	FALSE	1.779086372	2	17	0.19881424	0.19881424	FALSE	Intercept	563.5759562	1	563.5759562	18.85031974	0.000443371	PdgfraCre;Tbx1+/+	WT;Tbx1+/flox	1.934686976	12	1.044850385	0.076951997	0.126596757	FALSE
		WT;Tbx1+/+	6	6.17210563	1.907847722	0.972621635	0.90955982	0.90955982	FALSE							Genotype	127.1072061	2	63.55360307	2.125721876	0.14997335	PdgfraCre;Tbx1+/+	WT;Tbx1+/+	0.529258771	12	0.285832405	0.606278344	0.606278344	FALSE
		WT;Tbx1+/flox	6	1.937216264	1.116534507	0.751259955	0.02049345	0.061480349	FALSE							Error	508.2561668	17	29.89742157			WT;Tbx1+/flox	WT;Tbx1+/+	-1.915762576	10	-1.106066039	0.084397838	0.126596757	FALSE

							Shapiro-Wilk test				Levene test						Student t test							
Figure	Measurement	Month	Genotype	# of samples	Average	s.e.m	Statistic	p_value	p_adj(FDR BH)	Rejects(FDR 5%)	Statistic	df1	df2	p_value	p_adj(FDR BH)	Rejects(FDR 5%)	Sample1	Sample2	Statistic	dof	cohens_d	p_value	p_adj(FDR BH)	Rejects(FDR 5%)
Figure 7B	% open arm visits	1	oTbx1+/+	23	38.76689665	3.457186322	0.959194931	0.447303171	0.472928936	FALSE	0.034575631	1	32	0.853661423	0.853661423	FALSE	oTbx1+/-	oTbx1+/+	1.741236711	32	0.638317885	0.091249849	0.091249849	FALSE
			oTbx1+/-	11	48.92821047	4.328945934	0.935843176	0.472928936	0.472928936	FALSE														
		2	oTbx1+/+	20	16.20798761	2.580004845	0.933143261	0.177461607	0.339087396	FALSE	0.734877731	1	28	0.398584802	0.398584802	FALSE	oTbx1+/-	oTbx1+/+	0.59246246	28	0.229459724	0.558295668	0.558295668	FALSE
			oTbx1+/-	10	19.18868564	4.938885044	0.91781454	0.339087396	0.339087396	FALSE														

						Shapiro-Wilk test				Levene test						One way ANOVA						Student t test							
Measurement	Month	Genotype	# of samples	Average	s.e.m	Statistic	p_value	p_adj(FDR BH)	Rejects(FDR 5%)	Statistic	df1	df2	p_value	p_adj(FDR BH)	Rejects(FDR 5%)	Source	Type III Sum of Squares	df	Mean Square	F	Sig.	Sample1	Sample2	Statistic	dof	cohens_d	p_value	p_adj(FDR BH)	Rejects(FDR 5%)
% open arm visits	1	PdgfraCre;Tbx1+/+	7	35.6769162	4.664325787	0.928344033	0.536905413	0.536905413	FALSE	0.54245905	2	20	0.589631629	0.589631629	FALSE	Intercept	33992.03738	1	33992.03738	114.4766364	1.00324E-09	PdgfraCre;Tbx1+/+	WT;Tbx1+/flox	-0.38767913	14	-0.195371917	0.704083822	0.851686321	FALSE
		WT;Tbx1+/+	7	41.14160143	5.028883164	0.831111044	0.081981393	0.219437109	FALSE							Genotype	109.0958965	2	54.54794824	0.183703776	0.833570659	PdgfraCre;Tbx1+/+	WT;Tbx1+/+	-0.796719857	12	-0.425864677	0.441092294	0.851686321	FALSE
		WT;Tbx1+/flox	9	39.32322217	7.418792132	0.877086869	0.146291406	0.219437109	FALSE							Error	5938.685561	20	296.934278			WT;Tbx1+/flox	WT;Tbx1+/+	-0.190455488	14	-0.095980544	0.851686321	0.851686321	FALSE
	2	PdgfraCre;Tbx1+/+	8	20.47904805	3.951856074	0.921801353	0.444650175	0.666975262	FALSE	0.062288917	2	17	0.939824807	0.939824807	FALSE	Intercept	4860.798667	1	4860.798667	38.36512241	9.80937E-06	PdgfraCre;Tbx1+/+	WT;Tbx1+/flox	1.794257938	12	0.969010037	0.097985827	0.293957482	FALSE
		WT;Tbx1+/+	6	16.68154542	5.036940952	0.970955927	0.898793324	0.898793324	FALSE							Genotype	375.5691249	2	187.7845625	1.482138681	0.255070525	PdgfraCre;Tbx1+/+	WT;Tbx1+/+	0.602275965	12	0.325266196	0.558199622	0.558199622	FALSE
		WT;Tbx1+/flox	6	10.03968254	4.156071184	0.906492643	0.413726943	0.666975262	FALSE							Error	2153.872376	17	126.698375			WT;Tbx1+/flox	WT;Tbx1+/+	-1.017097195	10	-0.587221339	0.33308331	0.499624965	FALSE

Figure	Measurement	Month	Time (min)	Genotype	# of samples	Average	s.e.m	Shapiro-Wilk test Statistic	p_value	p_adj(FDR BH)	Rejects(FDR 5%)
Figure 8A	Total Distance (cm)	1	5	oTbx1+/+	24	523.3911589	30.20140117	0.975883028	0.809668571	0.809668571	FALSE
				oTbx1+/-	11	575.0133513	81.09884483	0.924914551	0.361718736	0.569470319	FALSE
			10	oTbx1+/+	24	428.858175	25.3219597	0.947354113	0.237276704	0.569470319	FALSE
				oTbx1+/-	11	463.1100966	54.68330524	0.916262967	0.288732783	0.569470319	FALSE
			15	oTbx1+/+	24	353.2098723	29.23769273	0.966833314	0.589787776	0.707745332	FALSE
				oTbx1+/-	11	411.012008	55.7960521	0.755590857	0.002459007	0.029508078	TRUE
			20	oTbx1+/+	24	323.8313083	27.24334579	0.975754462	0.806709631	0.809668571	FALSE
				oTbx1+/-	11	349.0746296	38.67701388	0.902850991	0.200221378	0.569470319	FALSE
			25	oTbx1+/+	24	264.9099236	32.94277932	0.903535486	0.02559625	0.153577498	FALSE
				oTbx1+/-	11	339.7488583	47.44333904	0.940451418	0.525819716	0.701092954	FALSE
			30	oTbx1+/+	24	246.8655593	33.32994361	0.947492768	0.238941106	0.569470319	FALSE
				oTbx1+/-	11	201.5505166	37.02036109	0.926826438	0.37964688	0.569470319	FALSE

Time (min)	Levene test Statistic	df1	df2	p_value	p_adj(FDR BH)	Rejects(FDR 5%)	Mann-Whitney U test Sample1	Sample2	Statistic	R (Z score)	p_value	p_adj(FDR BH)	Rejects(FDR 5%)
5	4.130581659	1	33	0.050226926	0.301361554	FALSE	oTbx1+/-	oTbx1+/+	125	0.042043748	0.82012655	0.902572012	FALSE
10	1.663209327	1	33	0.206136149	0.412272297	FALSE	oTbx1+/-	oTbx1+/+	126	0.036037499	0.84744042	0.902572012	FALSE
15	0.140317736	1	33	0.710361842	0.896645927	FALSE	oTbx1+/-	oTbx1+/+	128	0.024024999	0.902572012	0.902572012	FALSE
20	0.017135892	1	33	0.896645927	0.896645927	FALSE	oTbx1+/-	oTbx1+/+	120	0.072074997	0.687299671	0.902572012	FALSE
25	0.040522424	1	33	0.841698265	0.896645927	FALSE	oTbx1+/-	oTbx1+/+	93	0.23424374	0.173954599	0.902572012	FALSE
30	2.249666174	1	33	0.143150583	0.412272297	FALSE	oTbx1+/-	oTbx1+/+	111	-0.126131245	0.472220258	0.902572012	FALSE

Linear Mixed Model Source	F	df1	df2	Sig.
Corrected Model	14.18810262	11	198	0
Genotype	0.631374662	1	198	0.4278029
Time	27.84739692	5	198	0
Genotype * Time	0.955127959	5	198	0.446565974

Measurement	Month	Time (min)	Genotype	# of samples	Average	s.e.m	Shapiro-Wilk test Statistic	p_value	p_adj(FDR BH)	Rejects(FDR 5%)
Total Distance (cm)	1	5	PdgfraCre;Tbx1+/+	8	620.0439824	61.18380332	0.896772958	0.270160506	0.686126804	FALSE
			WT;Tbx1+/+	7	441.6039626	47.42794277	0.93556056	0.599115546	0.898673318	FALSE
			WT;Tbx1+/flox	9	501.0898018	31.91778323	0.918522745	0.380149455	0.760298911	FALSE
		10	PdgfraCre;Tbx1+/+	8	528.1841483	47.57087669	0.949171774	0.702877111	0.973214461	FALSE
			WT;Tbx1+/+	7	384.9748501	26.01161759	0.970333	0.900838417	0.980892361	FALSE
			WT;Tbx1+/flox	9	374.699896	33.33231151	0.977856565	0.952350694	0.980892361	FALSE
		15	PdgfraCre;Tbx1+/+	8	435.548186	56.01819145	0.984272937	0.980892361	0.980892361	FALSE
			WT;Tbx1+/+	7	331.29903	29.99137947	0.789411464	0.03213434	0.431236082	FALSE
			WT;Tbx1+/flox	9	297.0620262	48.93223185	0.905661169	0.286676525	0.686126804	FALSE
		20	PdgfraCre;Tbx1+/+	8	411.8585995	57.63072458	0.973883362	0.926616722	0.980892361	FALSE
			WT;Tbx1+/+	7	260.7565684	42.47375038	0.889114132	0.270074414	0.686126804	FALSE
			WT;Tbx1+/flox	9	294.6429583	26.46424959	0.856295547	0.087368002	0.524208011	FALSE
		25	PdgfraCre;Tbx1+/+	8	337.0979663	67.94142154	0.902611055	0.304945246	0.686126804	FALSE
			WT;Tbx1+/+	7	280.8983661	57.58848895	0.918866143	0.460635174	0.763439422	FALSE
			WT;Tbx1+/flox	9	188.3073192	38.18211176	0.832731267	0.04791512	0.431236082	FALSE
		30	PdgfraCre;Tbx1+/+	8	285.4238129	54.60920401	0.924397037	0.466546314	0.763439422	FALSE
			WT;Tbx1+/+	7	310.8749546	62.46942788	0.966178335	0.869648503	0.980892361	FALSE
			WT;Tbx1+/flox	9	162.8064708	49.74066212	0.878647793	0.15195339	0.683790257	FALSE

Time (min)	Levene test Statistic	df1	df2	p_value	p_adj(FDR BH)	Rejects(FDR 5%)
5	0.78676576	2	21	0.468282323	0.702423485	FALSE
10	1.114125751	2	21	0.346839968	0.693679937	FALSE
15	2.264993593	2	21	0.12860899	0.678212575	FALSE
20	1.597337999	2	21	0.226070858	0.678212575	FALSE
25	0.479360415	2	21	0.62579012	0.750948144	FALSE
30	0.105755822	2	21	0.900120365	0.900120365	FALSE

Repeated Measures ANOVA		Type III Sum of Squares	df	Mean Square	F	Sig.
**Within subjects**						
Time	Greenhouse-Geisser	1232066.871	3.551512016	346913.3331	18.43930669	6.42253E-10
Time * Genotype	Greenhouse-Geisser	149400.5872	7.103024031	21033.37769	1.11797635	0.361061454
Error(Time)	Greenhouse-Geisser	1403165.787	74.58175233	18813.79484		
**Between subjects**						
Intercept		18280351.96	1	18280351.96	417.5782475	2.43882E-15
Genotype		479372.5079	2	239686.254	5.475155296	0.012198718
Error		919318.4597	21	43777.06951		

Time (min)	Student t test Sample1	Sample2	Statistic	dof	cohens_d	p_value	p_adj(FDR BH)	Rejects(FDR 5%)
5	PdgfraCre;Tbx1+/+	WT;Tbx1+/flox	1.782324381	15	0.866053977	0.094944335	0.18988867	FALSE
	PdgfraCre;Tbx1+/+	WT;Tbx1+/+	2.254227774	13	1.166673712	0.042077619	0.182512156	FALSE
	WT;Tbx1+/flox	WT;Tbx1+/+	1.0781018	14	0.543312238	0.299214264	0.414296673	FALSE
10	PdgfraCre;Tbx1+/+	WT;Tbx1+/flox	2.69065136	15	1.307421554	0.016770132	0.182512156	FALSE
	PdgfraCre;Tbx1+/+	WT;Tbx1+/+	2.532927569	13	1.31091456	0.024986314	0.182512156	FALSE
	WT;Tbx1+/flox	WT;Tbx1+/+	-0.231693882	14	-0.116762741	0.820128066	0.820128066	FALSE
15	PdgfraCre;Tbx1+/+	WT;Tbx1+/flox	1.870796877	15	0.909043883	0.081011856	0.182512156	FALSE
	PdgfraCre;Tbx1+/+	WT;Tbx1+/+	1.571756012	13	0.813461019	0.140019642	0.22912305	FALSE
	WT;Tbx1+/flox	WT;Tbx1+/+	-0.554475257	14	-0.279429264	0.588002519	0.661502834	FALSE
20	PdgfraCre;Tbx1+/+	WT;Tbx1+/flox	1.92146405	15	0.933663704	0.073882743	0.182512156	FALSE
	PdgfraCre;Tbx1+/+	WT;Tbx1+/+	2.057488232	13	1.064851326	0.060276832	0.182512156	FALSE
	WT;Tbx1+/flox	WT;Tbx1+/+	0.70823831	14	0.35691856	0.490422016	0.630542591	FALSE
25	PdgfraCre;Tbx1+/+	WT;Tbx1+/flox	1.967124166	15	0.955850532	0.06794745	0.182512156	FALSE
	PdgfraCre;Tbx1+/+	WT;Tbx1+/+	0.620765095	13	0.321276459	0.545484347	0.654581216	FALSE
	WT;Tbx1+/flox	WT;Tbx1+/+	-1.390976099	14	-0.700986064	0.185945611	0.278918417	FALSE
30	PdgfraCre;Tbx1+/+	WT;Tbx1+/flox	1.663575002	15	0.808352151	0.116943757	0.210498763	FALSE
	PdgfraCre;Tbx1+/+	WT;Tbx1+/+	-0.308231337	13	-0.159524872	0.762789645	0.807659625	FALSE
	WT;Tbx1+/flox	WT;Tbx1+/+	-1.879743998	14	-0.947301933	0.081116514	0.182512156	FALSE

Figure	Measurement	Month	Time (min)	Genotype	# of samples	Average	s.e.m	Shapiro-Wilk test Statistic	p_value	p_adj(FDR BH)	Rejects(FDR 5%)
Figure 8B	Total Distance (cm)	2	5	oTbx1+/+	20	582.5324674	39.81366145	0.961805113	0.580510261	0.964595553	FALSE
				oTbx1+/-	10	565.9321735	87.02480436	0.952194518	0.694501499	0.964595553	FALSE
			10	oTbx1+/+	20	465.0152343	28.92161572	0.976249885	0.877119898	0.964595553	FALSE
				oTbx1+/-	10	478.4165818	52.4441209	0.848827274	0.056251555	0.675018657	FALSE
			15	oTbx1+/+	20	361.7175911	28.74548525	0.979861932	0.932306056	0.964595553	FALSE
				oTbx1+/-	10	409.8879946	50.49069356	0.979893494	0.964595553	0.964595553	FALSE
			20	oTbx1+/+	20	348.215865	27.97211407	0.978671917	0.915818525	0.964595553	FALSE
				oTbx1+/-	10	300.6010342	50.30866599	0.93172014	0.465023493	0.964595553	FALSE
			25	oTbx1+/+	20	299.1055367	24.94704284	0.974019572	0.836412834	0.964595553	FALSE
				oTbx1+/-	10	264.0208722	50.34200893	0.938023496	0.531237334	0.964595553	FALSE
			30	oTbx1+/+	20	255.362162	39.42744784	0.949277247	0.356252313	0.964595553	FALSE
				oTbx1+/-	10	252.3293176	51.6114029	0.940717398	0.56102342	0.964595553	FALSE

Time (min)	Levene test Statistic	df1	df2	p_value	p_adj(FDR BH)	Rejects(FDR 5%)
5	2.027577552	1	28	0.165516111	0.496548332	FALSE
10	3.110488944	1	28	0.088700281	0.496548332	FALSE
15	0.864912718	1	28	0.360316817	0.530231581	FALSE
20	0.608602693	1	28	0.441859651	0.530231581	FALSE
25	0.876820379	1	28	0.357080786	0.530231581	FALSE
30	0.036907756	1	28	0.849039501	0.849039501	FALSE

Repeated Measures ANOVA		Type III Sum of Squares	df	Mean Square	F	Sig.
**Within Subjects**						
Time	Greenhouse-Geisser	1995853.076	3.660164031	545290.6098	26.86051187	1.71481E-14
Time * Genotype	Greenhouse-Geisser	40039.66219	3.660164031	10939.30814	0.538860217	0.691877512
Error(Time)	Greenhouse-Geisser	2080522.01	102.4845929	20300.82719		
**Between Subjects**						
Intercept		23339048.01	1	23339048.01	306.4591211	1.2929E-16
Genotype		1846.055051	1	1846.055051	0.024240081	0.87739263
Error		2132399.721	28	76157.13288		

Time (min)	Student t test Sample1	Sample2	Statistic	dof	cohens_d	p_value	p_adj(FDR BH)	Rejects(FDR 5%)
5	oTbx1+/-	oTbx1+/+	-0.200159227	28	-0.077521335	0.842802741	0.9640554	FALSE
10	oTbx1+/-	oTbx1+/+	0.243505064	28	0.094309106	0.809389409	0.9640554	FALSE
15	oTbx1+/-	oTbx1+/+	0.892772198	28	0.345769186	0.379588252	0.9640554	FALSE
20	oTbx1+/-	oTbx1+/+	-0.897736865	28	-0.347691993	0.376980718	0.9640554	FALSE
25	oTbx1+/-	oTbx1+/+	-0.703264717	28	-0.272373254	0.487697769	0.9640554	FALSE
30	oTbx1+/-	oTbx1+/+	-0.045470044	28	-0.017610472	0.9640554	0.9640554	FALSE

Measurement	Month	Time (min)	Genotype	# of samples	Average	s.e.m	Shapiro-Wilk test Statistic	p_value	p_adj(FDR BH)	Rejects(FDR 5%)
Total Distance (cm)	2	5	PdgfraCre;Tbx1+/+	8	675.3377481	67.21069573	0.960970017	0.819288618	0.90097253	FALSE
			WT;Tbx1+/+	6	576.9360505	60.27675931	0.947046048	0.716310322	0.90097253	FALSE
			WT;Tbx1+/flox	6	464.38851	54.01971119	0.932193779	0.597146871	0.90097253	FALSE
		10	PdgfraCre;Tbx1+/+	8	563.3077705	39.36525666	0.91827703	0.416039464	0.90097253	FALSE
			WT;Tbx1+/+	6	423.8291928	36.86712279	0.939986406	0.659089302	0.90097253	FALSE
			WT;Tbx1+/flox	6	375.1445608	42.57179879	0.896122527	0.351518024	0.90097253	FALSE
		15	PdgfraCre;Tbx1+/+	8	412.9964819	53.1536322	0.970729632	0.903671601	0.903671601	FALSE
			WT;Tbx1+/+	6	384.9906002	24.8709437	0.960076551	0.820330406	0.90097253	FALSE
			WT;Tbx1+/flox	6	270.0727277	45.65896481	0.885730035	0.296415795	0.90097253	FALSE
		20	PdgfraCre;Tbx1+/+	8	383.3626534	47.27633929	0.962488762	0.833447547	0.90097253	FALSE
			WT;Tbx1+/+	6	392.8098267	35.00674422	0.93743225	0.638585233	0.90097253	FALSE
			WT;Tbx1+/flox	6	256.7595188	45.97104537	0.889647485	0.316344886	0.90097253	FALSE
		25	PdgfraCre;Tbx1+/+	8	364.6078814	41.60013821	0.951382549	0.72516182	0.90097253	FALSE
			WT;Tbx1+/+	6	296.9929018	29.92382991	0.964127822	0.8509185	0.90097253	FALSE
			WT;Tbx1+/flox	6	213.881712	34.05386391	0.910326291	0.438546447	0.90097253	FALSE
		30	PdgfraCre;Tbx1+/+	8	325.6745739	75.49961398	0.953377125	0.745179396	0.90097253	FALSE
			WT;Tbx1+/+	6	277.1861365	55.16889694	0.929847805	0.578927051	0.90097253	FALSE
			WT;Tbx1+/flox	6	139.7883048	43.83595818	0.905323563	0.40635214	0.90097253	FALSE

Time (min)	Levene test Statistic	df1	df2	p_value	p_adj(FDR BH)	Rejects(FDR 5%)
5	0.513944781	2	17	0.60713361	0.845882843	FALSE
10	0.142545825	2	17	0.86817344	0.86817344	FALSE
15	1.538099909	2	17	0.243233282	0.518147214	FALSE
20	0.356989008	2	17	0.704902369	0.845882843	FALSE
25	1.850493244	2	17	0.187452819	0.518147214	FALSE
30	1.463867976	2	17	0.259073607	0.518147214	FALSE

Repeated Measures ANOVA		Type III Sum of Squares	df	Mean Square	F	Sig.
**Within subjects**						
Time	Sphericity Assumed	1366149.157	5	273229.8314	19.2608065	9.05321E-13
Time * Genotype	Sphericity Assumed	56104.56039	10	5610.456039	0.395498206	0.94529822
Error(Time)	Sphericity Assumed	1205792.482	85	14185.7939		
**Between subjects**						
Intercept		16804988	1	16804988	552.5624347	2.10342E-14
Genotype		579823.9342	2	289911.9671	9.532554405	0.001673302
Error		517018.1288	17	30412.83111		

Time (min)	Student t test Sample1	Sample2	Statistic	dof	cohens_d	p_value	p_adj(FDR BH)	Rejects(FDR 5%)
5	PdgfraCre;Tbx1+/+	WT;Tbx1+/flox	2.318778914	12	1.25228374	0.038853127	0.145180864	FALSE
	PdgfraCre;Tbx1+/+	WT;Tbx1+/+	1.049097419	12	0.566577362	0.314803416	0.404747249	FALSE
	WT;Tbx1+/flox	WT;Tbx1+/+	-1.390491666	10	-0.802800737	0.194547247	0.291820871	FALSE
10	PdgfraCre;Tbx1+/+	WT;Tbx1+/flox	3.212491801	12	1.734943863	0.007457649	0.134237677	FALSE
	PdgfraCre;Tbx1+/+	WT;Tbx1+/+	2.504995321	12	1.352852094	0.027660714	0.145180864	FALSE
	WT;Tbx1+/flox	WT;Tbx1+/+	-0.864483291	10	-0.499109661	0.407581081	0.489097297	FALSE
15	PdgfraCre;Tbx1+/+	WT;Tbx1+/flox	1.951155687	12	1.053744506	0.074776993	0.154898089	FALSE
	PdgfraCre;Tbx1+/+	WT;Tbx1+/+	0.427254885	12	0.23074401	0.676759782	0.716569181	FALSE
	WT;Tbx1+/flox	WT;Tbx1+/+	-2.210243188	10	-1.2760845	0.051539445	0.154618335	FALSE
20	PdgfraCre;Tbx1+/+	WT;Tbx1+/flox	1.870093843	12	1.009966107	0.086054494	0.154898089	FALSE
	PdgfraCre;Tbx1+/+	WT;Tbx1+/+	-0.150587648	12	-0.081326625	0.882802665	0.882802665	FALSE
	WT;Tbx1+/flox	WT;Tbx1+/+	-2.354527304	10	-1.359386973	0.040328018	0.145180864	FALSE
25	PdgfraCre;Tbx1+/+	WT;Tbx1+/flox	2.664033886	12	1.438742736	0.02063703	0.145180864	FALSE
	PdgfraCre;Tbx1+/+	WT;Tbx1+/+	1.232745856	12	0.665758853	0.241276061	0.334074546	FALSE
	WT;Tbx1+/flox	WT;Tbx1+/+	-1.833339471	10	-1.058479037	0.096646438	0.158148716	FALSE
30	PdgfraCre;Tbx1+/+	WT;Tbx1+/flox	1.942251174	12	1.048935519	0.075945879	0.154898089	FALSE
	PdgfraCre;Tbx1+/+	WT;Tbx1+/+	0.485421275	12	0.262157451	0.636119338	0.715634256	FALSE
	WT;Tbx1+/flox	WT;Tbx1+/+	-1.949896567	10	-1.125773308	0.079761998	0.154898089	FALSE

Figure	Measurement	Month	Time (min)	Genotype	# of samples	Average	s.e.m	Shapiro-Wilk test Statistic	p_value	p_adj(FDR BH)	Rejects(FDR 5%)
Figure 8C	Total Margin Time (s)	1	5	oTbx1+/+	24	187.9541667	9.818642574	0.989732318	0.995574805	0.995574805	FALSE
				oTbx1+/-	11	224.8954545	14.69387698	0.929218262	0.402999507	0.592165394	FALSE
			10	oTbx1+/+	24	177.46875	8.621859233	0.960282408	0.444124046	0.592165394	FALSE
				oTbx1+/-	11	202.8863636	9.029917675	0.968324319	0.869129208	0.948140954	FALSE
			15	oTbx1+/+	24	199.6520833	11.65783522	0.959487016	0.428203171	0.592165394	FALSE
				oTbx1+/-	11	211.4636364	13.35909988	0.948946345	0.630518333	0.756622	FALSE
			20	oTbx1+/+	24	224.575	13.89428188	0.864461767	0.004105455	0.012316364	TRUE
				oTbx1+/-	11	212.5727273	18.03323745	0.905116019	0.213240511	0.426481022	FALSE
			25	oTbx1+/+	24	238.9916667	11.76924598	0.78965836	0.000200552	0.001244162	TRUE
				oTbx1+/-	11	221.3590909	16.82044727	0.889299635	0.136293398	0.327104154	FALSE
			30	oTbx1+/+	24	255.1479167	10.74757408	0.790566413	0.00020736	0.001244162	TRUE
				oTbx1+/-	11	247.8272727	16.96605483	0.755966079	0.002486921	0.009947684	TRUE

Time (min)	Levene test Statistic	df1	df2	p_value	p_adj(FDR BH)	Rejects(FDR 5%)
5	0.044942279	1	33	0.833414161	0.88610226	FALSE
10	1.749233955	1	33	0.195066221	0.88610226	FALSE
15	0.97523234	1	33	0.330564543	0.88610226	FALSE
20	0.073888102	1	33	0.787451039	0.88610226	FALSE
25	0.459512459	1	33	0.502577518	0.88610226	FALSE
30	0.020836853	1	33	0.88610226	0.88610226	FALSE

Linear Mixed Model Source	F	df1	df2	Sig.
Corrected Model	6.693346578	11	198	1.67687E-09
Genotype	0.200852805	1	198	0.654523373
Time	8.251156703	5	198	4.22922E-07
Genotype * Time	2.151693449	5	198	0.06094955

Time (min)	Mann-Whitney U test Sample1	Sample2	Statistic	R (Z score)	p_value	p_adj(FDR BH)	Rejects(FDR 5%)
5	oTbx1+/-	oTbx1+/+	79	0.318331237	0.061404045	0.200151386	FALSE
10	oTbx1+/-	oTbx1+/+	80	0.312324987	0.066717129	0.200151386	FALSE
15	oTbx1+/-	oTbx1+/+	118	0.084087497	0.63644326	0.63644326	FALSE
20	oTbx1+/-	oTbx1+/+	105	-0.162168743	0.351863535	0.540916088	FALSE
25	oTbx1+/-	oTbx1+/+	109.5	-0.135140619	0.450763407	0.540916088	FALSE
30	oTbx1+/-	oTbx1+/+	109	-0.138143744	0.429851852	0.540916088	FALSE

Measurement	Month	Time (min)	Genotype	# of samples	Average	s.e.m	Shapiro-Wilk test Statistic	p_value	p_adj(FDR BH)	Rejects(FDR 5%)
Total Margin Time (s)	1	5	PdgfraCre;Tbx1+/+	8	192.4	19.42002142	0.940119442	0.612255906	0.795951402	FALSE
			WT;Tbx1+/+	7	183.6142857	18.45076045	0.951934955	0.747275189	0.836773791	FALSE
			WT;Tbx1+/flox	9	187.3777778	15.59257549	0.878358778	0.150890078	0.388003057	FALSE
		10	PdgfraCre;Tbx1+/+	8	176.525	17.77269173	0.852568912	0.10114029	0.303420871	FALSE
			WT;Tbx1+/+	7	178.4642857	12.14288305	0.937809188	0.619073312	0.795951402	FALSE
			WT;Tbx1+/flox	9	177.5333333	15.42819389	0.958946118	0.787226042	0.836773791	FALSE
		15	PdgfraCre;Tbx1+/+	8	190.74375	21.81628945	0.976457289	0.943303265	0.943303265	FALSE
			WT;Tbx1+/+	7	207.3857143	23.9748646	0.793291374	0.035136728	0.128541595	FALSE
			WT;Tbx1+/flox	9	201.5555556	17.9268991	0.939932071	0.581183632	0.795951402	FALSE
		20	PdgfraCre;Tbx1+/+	8	217.70625	22.27686468	0.930651593	0.522025005	0.795951402	FALSE
			WT;Tbx1+/+	7	262.8928571	13.23386002	0.882178317	0.236294206	0.531661963	FALSE
			WT;Tbx1+/flox	9	200.8777778	27.81605266	0.821368373	0.035705999	0.128541595	FALSE
		25	PdgfraCre;Tbx1+/+	8	241.09375	16.03556994	0.932714563	0.541105179	0.795951402	FALSE
			WT;Tbx1+/+	7	260.0642857	6.701019061	0.956735323	0.790286358	0.836773791	FALSE
			WT;Tbx1+/flox	9	220.7333333	27.39387604	0.785658445	0.013990261	0.106253968	FALSE
		30	PdgfraCre;Tbx1+/+	8	266.31875	13.4645697	0.780658686	0.017708995	0.106253968	FALSE
			WT;Tbx1+/+	7	249.7428571	17.73063138	0.907420109	0.378299157	0.756598315	FALSE
			WT;Tbx1+/flox	9	249.4222222	23.24614531	0.741618208	0.004333717	0.078006906	FALSE

Time (min)	Levene test Statistic	df1	df2	p_value	p_adj(FDR BH)	Rejects(FDR 5%)
5	0.155922633	2	21	0.856606813	0.96116231	FALSE
10	0.275926248	2	21	0.761577722	0.96116231	FALSE
15	0.039686801	2	21	0.96116231	0.96116231	FALSE
20	1.011932031	2	21	0.380567208	0.96116231	FALSE
25	1.824164579	2	21	0.186009019	0.96116231	FALSE
30	0.45938567	2	21	0.637870373	0.96116231	FALSE

Repeated Measures ANOVA		Type III Sum of Squares	df	Mean Square	F	Sig.
**Within subjects**						
Time	Greenhouse-Geisser	113778.7452	2.742258347	41490.89212	12.81272325	3.10589E-06
Time * Genotype	Greenhouse-Geisser	17553.4018	5.484516693	3200.537582	0.988351906	0.437454084
Error(Time)	Greenhouse-Geisser	186482.8892	57.58742528	3238.257107		
**Between subjects**						
Intercept		6567827.009	1	6567827.009	673.365646	1.94254E-17
Genotype		7190.956262	2	3595.478131	0.368625948	0.696071568
Error		204828.3396	21	9753.730455		

Time (min)	Student t test Sample1	Sample2	Statistic	dof	cohens_d	p_value	p_adj(FDR BH)	Rejects(FDR 5%)
5	PdgfraCre;Tbx1+/+	WT;Tbx1+/flox	0.203680516	15	0.098970941	0.841340647	0.991826617	FALSE
	PdgfraCre;Tbx1+/+	WT;Tbx1+/+	0.325226959	13	0.168320942	0.750184167	0.991826617	FALSE
	WT;Tbx1+/flox	WT;Tbx1+/+	0.156685574	14	0.078962107	0.877729762	0.991826617	FALSE
10	PdgfraCre;Tbx1+/+	WT;Tbx1+/flox	-0.043066059	15	-0.020926343	0.96621693	0.991826617	FALSE
	PdgfraCre;Tbx1+/+	WT;Tbx1+/+	-0.087424034	13	-0.045246236	0.931666773	0.991826617	FALSE
	WT;Tbx1+/flox	WT;Tbx1+/+	-0.045251736	14	-0.022804731	0.964545789	0.991826617	FALSE
15	PdgfraCre;Tbx1+/+	WT;Tbx1+/flox	-0.386193777	15	-0.187656445	0.704775853	0.991826617	FALSE
	PdgfraCre;Tbx1+/+	WT;Tbx1+/+	-0.514417762	13	-0.266236486	0.615593079	0.991826617	FALSE
	WT;Tbx1+/flox	WT;Tbx1+/+	-0.199074094	14	-0.100323913	0.845066999	0.991826617	FALSE
20	PdgfraCre;Tbx1+/+	WT;Tbx1+/flox	0.464184705	15	0.225553224	0.649180802	0.991826617	FALSE
	PdgfraCre;Tbx1+/+	WT;Tbx1+/+	-1.67915527	13	-0.869045415	0.116981237	0.991826617	FALSE
	WT;Tbx1+/flox	WT;Tbx1+/+	-1.833493431	14	-0.923993838	0.088073727	0.991826617	FALSE
25	PdgfraCre;Tbx1+/+	WT;Tbx1+/flox	0.620368342	15	0.30144483	0.544326164	0.991826617	FALSE
	PdgfraCre;Tbx1+/+	WT;Tbx1+/+	-1.035608398	13	-0.535978266	0.319265725	0.991826617	FALSE
	WT;Tbx1+/flox	WT;Tbx1+/+	-1.234919708	14	-0.622341035	0.23718542	0.991826617	FALSE
30	PdgfraCre;Tbx1+/+	WT;Tbx1+/flox	0.608023955	15	0.295446536	0.552267228	0.991826617	FALSE
	PdgfraCre;Tbx1+/+	WT;Tbx1+/+	0.755605871	13	0.391063191	0.463357322	0.991826617	FALSE
	WT;Tbx1+/flox	WT;Tbx1+/+	-0.01042843	14	-0.005255435	0.991826617	0.991826617	FALSE

Figure	Measurement	Month	Time (min)	Genotype	# of samples	Average	s.e.m	Shapiro-Wilk test Statistic	p_value	p_adj(FDR BH)	Rejects(FDR 5%)	Levene test Statistic	df1	df2	p_value	p_adj(FDR BH)	Rejects(FDR 5%)
Figure 8D	Total Margin Time (s)	2	5	oTbx1+/+	20	206.8325	12.85423966	0.930050585	0.154763372	0.371432094	FALSE	0.018345627	1	28	0.893228286	0.893228286	FALSE
				oTbx1+/-	10	214.465	18.6721823	0.924244799	0.393721373	0.472465648	FALSE						
			10	oTbx1+/+	20	189.3575	12.22481583	0.94401407	0.285201157	0.427801736	FALSE	0.528505896	1	28	0.473270499	0.893228286	FALSE
				oTbx1+/-	10	198.145	15.69639635	0.863931639	0.08488746	0.25466238	FALSE						
			15	oTbx1+/+	20	219.36	12.95180672	0.943204905	0.275461326	0.427801736	FALSE	0.293845397	1	28	0.59205509	0.893228286	FALSE
				oTbx1+/-	10	192.6	16.68604041	0.922021218	0.374121624	0.472465648	FALSE						
			20	oTbx1+/+	20	225.285	15.17396884	0.870559134	0.012018056	0.048072225	TRUE	0.875553509	1	28	0.357423105	0.893228286	FALSE
				oTbx1+/-	10	219.055	16.35034395	0.973597897	0.922052465	0.922052465	FALSE						
			25	oTbx1+/+	20	243.54	12.86533725	0.847368866	0.004817268	0.028903611	TRUE	0.13461714	1	28	0.716450392	0.893228286	FALSE
				oTbx1+/-	10	240.37	14.0183693	0.908087267	0.268109319	0.427801736	FALSE						
			30	oTbx1+/+	20	258.69	10.77157297	0.763194126	0.000256532	0.003078381	TRUE	0.021812291	1	28	0.883645943	0.893228286	FALSE
				oTbx1+/-	10	246.795	13.5509685	0.941920819	0.574578388	0.626812787	FALSE						

Linear Mixed Model

Source	F	df1	df2	Sig.
Corrected Model	6.617692766	11	168	3.86918E-09
Genotype	0.089121278	1	168	0.765666179
Time	11.39367746	5	168	1.76945E-09
Genotype * Time	0.987985386	5	168	0.426725453

Mann-Whitney U test

Time (min)	Sample1	Sample2	Statistic	R (Z score)	p_value	p_adj(FDR BH)	Rejects(FDR 5%)
5	oTbx1+/-	oTbx1+/+	95	0.040160966	0.845778642	0.845778642	FALSE
10	oTbx1+/-	oTbx1+/+	91	0.07228974	0.713163831	0.845778642	FALSE
15	oTbx1+/-	oTbx1+/+	64	-0.289158958	0.11962883	0.717772982	FALSE
20	oTbx1+/-	oTbx1+/+	87	-0.104418513	0.588437383	0.845778642	FALSE
25	oTbx1+/-	oTbx1+/+	85	-0.120482899	0.529969714	0.845778642	FALSE
30	oTbx1+/-	oTbx1+/+	77	-0.184740446	0.328175706	0.845778642	FALSE

Measurement	Month	Time (min)	Genotype	# of samples	Average	s.e.m	Shapiro-Wilk test Statistic	p_value	p_adj(FDR BH)	Rejects(FDR 5%)	Levene test Statistic	df1	df2	p_value	p_adj(FDR BH)	Rejects(FDR 5%)
Total Margin Time (s)	2	5	PdgfraCre;Tbx1+/+	8	215.05	20.7141213	0.914114559	0.383949912	0.589193044	FALSE	2.353529985	2	17	0.125229683	0.324892303	FALSE
			WT;Tbx1+/+	6	155.8916667	13.65221238	0.920982548	0.512465635	0.658884388	FALSE						
			WT;Tbx1+/flox	6	246.8166667	15.77535349	0.902472395	0.388750333	0.589193044	FALSE						
		10	PdgfraCre;Tbx1+/+	8	192.06875	20.35318275	0.869602244	0.149385243	0.537786875	FALSE	0.703121363	2	17	0.508876155	0.610651385	FALSE
			WT;Tbx1+/+	6	149.775	19.35028747	0.957394761	0.799454028	0.899385782	FALSE						
			WT;Tbx1+/flox	6	225.325	13.08559482	0.903135512	0.392795363	0.589193044	FALSE						
		15	PdgfraCre;Tbx1+/+	8	210.59375	24.92307171	0.904635296	0.317826456	0.589193044	FALSE	2.176534062	2	17	0.144013566	0.324892303	FALSE
			WT;Tbx1+/+	6	186.4166667	12.48941997	0.975341879	0.926200355	0.96371726	FALSE						
			WT;Tbx1+/flox	6	263.9916667	13.16809539	0.926026806	0.549786816	0.659744179	FALSE						
		20	PdgfraCre;Tbx1+/+	8	214.0375	28.49870415	0.834033939	0.065373269	0.392239615	FALSE	1.521800567	2	17	0.246616393	0.36992459	FALSE
			WT;Tbx1+/+	6	189.425	21.96758123	0.982597585	0.96371726	0.96371726	FALSE						
			WT;Tbx1+/flox	6	276.1416667	10.47147249	0.752640586	0.021139106	0.190251956	FALSE						
		25	PdgfraCre;Tbx1+/+	8	231.325	19.64722127	0.925406735	0.47524764	0.658035195	FALSE	2.026321741	2	17	0.162446151	0.324892303	FALSE
			WT;Tbx1+/+	6	218.1083333	28.53045863	0.887468781	0.305136927	0.589193044	FALSE						
			WT;Tbx1+/flox	6	285.2583333	6.691492318	0.829589393	0.106631799	0.479843093	FALSE						
		30	PdgfraCre;Tbx1+/+	8	258.3625	18.83312467	0.716307697	0.00343956	0.061912072	FALSE	0.476100124	2	17	0.629238835	0.629238835	FALSE
			WT;Tbx1+/+	6	232.4333333	21.96380229	0.884449517	0.290118872	0.589193044	FALSE						
			WT;Tbx1+/flox	6	285.3833333	6.690623621	0.871648175	0.232854122	0.589193044	FALSE						

Repeated Measures ANOVA

		Type III Sum of Squares	df	Mean Square	F	Sig.
**Within subjects**						
Time	Greenhouse-Geisser	63034.55554	2.524553611	24968.59455	12.07218449	2.00728E-05
Time * Genotype	Greenhouse-Geisser	7497.715639	5.049107223	1484.958688	0.717969736	0.61474568
Error(Time)	Greenhouse-Geisser	88764.99903	42.9174114	2068.274766		
**Between subjects**						
Intercept		5924566.762	1	5924566.762	563.1055124	1.79933E-14
Genotype		102679.8172	2	51339.90862	4.879645501	0.021148604
Error		178861.0353	17	10521.23737		

Student t test

Time (min)	Sample1	Sample2	Statistic	dof	cohens_d	p_value	p_adj(FDR BH)	Rejects(FDR 5%)
5	PdgfraCre;Tbx1+/+	WT;Tbx1+/flox	-1.14816594	12	-0.620080478	0.273274002	0.351352288	FALSE
	PdgfraCre;Tbx1+/+	WT;Tbx1+/+	2.204819261	12	1.190738493	0.047722279	0.107375127	FALSE
	WT;Tbx1+/flox	WT;Tbx1+/+	4.358294506	10	2.516262506	0.00142495	0.014632215	TRUE
10	PdgfraCre;Tbx1+/+	WT;Tbx1+/flox	-1.267237854	12	-0.684386661	0.229111121	0.343666681	FALSE
	PdgfraCre;Tbx1+/+	WT;Tbx1+/+	1.46200151	12	0.789571057	0.169430463	0.277249849	FALSE
	WT;Tbx1+/flox	WT;Tbx1+/+	3.234229662	10	1.867283366	0.008956536	0.040304412	TRUE
15	PdgfraCre;Tbx1+/+	WT;Tbx1+/flox	-1.712823843	12	-0.925030599	0.1124384	0.20238912	FALSE
	PdgfraCre;Tbx1+/+	WT;Tbx1+/+	0.780634653	12	0.421590897	0.450142746	0.506410589	FALSE
	WT;Tbx1+/flox	WT;Tbx1+/+	4.274352212	10	2.4677984	0.001625802	0.014632215	TRUE
20	PdgfraCre;Tbx1+/+	WT;Tbx1+/flox	-1.803787117	12	-0.974156382	0.09640862	0.192817241	FALSE
	PdgfraCre;Tbx1+/+	WT;Tbx1+/+	0.644726307	12	0.348192001	0.531236864	0.562486091	FALSE
	WT;Tbx1+/flox	WT;Tbx1+/+	3.563351857	10	2.057302154	0.005152424	0.030914546	TRUE
25	PdgfraCre;Tbx1+/+	WT;Tbx1+/flox	-2.283065064	12	-1.232996056	0.041448022	0.107375127	FALSE
	PdgfraCre;Tbx1+/+	WT;Tbx1+/+	0.395110604	12	0.213384114	0.699694601	0.699694601	FALSE
	WT;Tbx1+/flox	WT;Tbx1+/+	2.291444377	10	1.322966028	0.044903228	0.107375127	FALSE
30	PdgfraCre;Tbx1+/+	WT;Tbx1+/flox	-1.190208127	12	-0.642785854	0.256978702	0.351352288	FALSE
	PdgfraCre;Tbx1+/+	WT;Tbx1+/+	0.897571615	12	0.484744074	0.387067972	0.464481566	FALSE
	WT;Tbx1+/flox	WT;Tbx1+/+	2.306159612	10	1.331461873	0.043792782	0.107375127	FALSE

Measurement	Month	Time (min)	Genotype	# of samples	Average	s.e.m	Shapiro-Wilk test Statistic	p_value	p_adj(FDR BH)	Rejects(FDR 5%)	Levene test Statistic	df1	df2	p_value	p_adj(FDR BH)	Rejects(FDR 5%)
Total Margin Time (s)	2	5	PdgfraCre;Tbx1+/+	8	215.05	20.7141213	0.914114559	0.383949912	0.59058206	FALSE	2.021947392	3	26	0.13547732	0.485523172	FALSE
			PdgfraCre;Tbx1+/flox	10	214.465	18.6721823	0.924244799	0.393721373	0.59058206	FALSE						
			WT;Tbx1+/+	6	155.8916667	13.65221238	0.920982548	0.512465635	0.683287514	FALSE						
			WT;Tbx1+/flox	6	246.8166667	15.77535349	0.902472395	0.388750333	0.59058206	FALSE						
		10	PdgfraCre;Tbx1+/+	8	192.06875	20.35318275	0.869602244	0.149385243	0.59058206	FALSE	0.46733694	3	26	0.707587378	0.707587378	FALSE
			PdgfraCre;Tbx1+/flox	10	198.145	15.69639635	0.863931639	0.08488746	0.50932476	FALSE						
			WT;Tbx1+/+	6	149.775	19.35028747	0.957394761	0.799454028	0.913661747	FALSE						
			WT;Tbx1+/flox	6	225.325	13.08559482	0.903135512	0.392795363	0.59058206	FALSE						
		15	PdgfraCre;Tbx1+/+	8	210.59375	24.92307171	0.904635296	0.317826456	0.59058206	FALSE	1.366979197	3	26	0.274814022	0.500050269	FALSE
			PdgfraCre;Tbx1+/flox	10	192.6	16.68604041	0.922021218	0.374121624	0.59058206	FALSE						
			WT;Tbx1+/+	6	186.4166667	12.48941997	0.975341879	0.926200355	0.96371726	FALSE						
			WT;Tbx1+/flox	6	263.9916667	13.16809539	0.926026806	0.549786816	0.689494065	FALSE						
		20	PdgfraCre;Tbx1+/+	8	214.0375	28.49870415	0.834033939	0.065373269	0.50932476	FALSE	1.188722003	3	26	0.333366846	0.500050269	FALSE
			PdgfraCre;Tbx1+/flox	10	219.055	16.35034395	0.973597897	0.922052465	0.96371726	FALSE						
			WT;Tbx1+/+	6	189.425	21.96758123	0.982597585	0.96371726	0.96371726	FALSE						
			WT;Tbx1+/flox	6	276.1416667	10.47147249	0.752640586	0.021139106	0.253669274	FALSE						
		25	PdgfraCre;Tbx1+/+	8	231.325	19.64722127	0.925406735	0.47524764	0.670937845	FALSE	1.856257447	3	26	0.161841057	0.485523172	FALSE
			PdgfraCre;Tbx1+/flox	10	240.37	14.0183693	0.908087267	0.268109319	0.59058206	FALSE						
			WT;Tbx1+/+	6	218.1083333	28.53045863	0.887468781	0.305136927	0.59058206	FALSE						
			WT;Tbx1+/flox	6	285.2583333	6.691492318	0.829589393	0.106631799	0.511832633	FALSE						
		30	PdgfraCre;Tbx1+/+	8	258.3625	18.83312467	0.716307697	0.00343956	0.082549429	FALSE	0.505235134	3	26	0.682057595	0.707587378	FALSE
			PdgfraCre;Tbx1+/flox	10	246.795	13.5509685	0.941920819	0.574578388	0.689494065	FALSE						
			WT;Tbx1+/+	6	232.4333333	21.96380229	0.884449517	0.290118872	0.59058206	FALSE						
			WT;Tbx1+/flox	6	285.3833333	6.690623621	0.871648175	0.232854122	0.59058206	FALSE						

Repeated Measures ANOVA

		Type III Sum of Squares	df	Mean Square	F	Sig.
**Within subject**						
Time	Greenhouse-Geisser	80940.86107	2.72028392	29754.56366	13.33360251	1.23598E-06
Time * Genotype	Greenhouse-Geisser	13331.91631	8.160851761	1633.642749	0.7320673	0.665388825
Error(Time)	Greenhouse-Geisser	157831.4927	70.72738193	2231.547222		
**Between Subject**						
Intercept		8537113.515	1	8537113.515	899.8162227	1.06132E-21
Genotype		103791.7875	3	34597.2625	3.646569534	0.025572586
Error		246678.0947	26	9487.619026		

Student t test

Time (min)	Sample1	Sample2	Statistic	dof	cohens_d	p_value	p_adj(FDR BH)	Rejects(FDR 5%)
5	PdgfraCre;Tbx1+/flc	PdgfraCre;Tbx1	-0.020957687	16	-0.009941104	0.983538546	0.983538546	FALSE
	PdgfraCre;Tbx1+/flc	WT;Tbx1+/flox	-1.189356816	14	-0.614181219	0.254080479	0.440534918	FALSE
	PdgfraCre;Tbx1+/flc	WT;Tbx1+/+	2.207266313	14	1.139827423	0.044489921	0.143166836	FALSE
	PdgfraCre;Tbx1+/+	WT;Tbx1+/flox	-1.14816594	12	-0.620080478	0.273274002	0.447175639	FALSE
	PdgfraCre;Tbx1+/+	WT;Tbx1+/+	2.204819261	12	1.190738493	0.047722279	0.143166836	FALSE
	WT;Tbx1+/flox	WT;Tbx1+/+	4.358294506	10	2.516262506	0.00142495	0.02926443	TRUE
10	PdgfraCre;Tbx1+/flc	PdgfraCre;Tbx1	0.240552709	16	0.114104169	0.81295694	0.860777937	FALSE
	PdgfraCre;Tbx1+/flc	WT;Tbx1+/flox	-1.19168395	14	-0.615382946	0.253195562	0.440534918	FALSE
	PdgfraCre;Tbx1+/flc	WT;Tbx1+/+	1.917509311	14	0.990197551	0.075806824	0.194931833	FALSE
	PdgfraCre;Tbx1+/+	WT;Tbx1+/flox	-1.267237854	12	-0.684386661	0.229111121	0.440534918	FALSE
	PdgfraCre;Tbx1+/+	WT;Tbx1+/+	1.46200151	12	0.789571057	0.169430463	0.358793922	FALSE
	WT;Tbx1+/flox	WT;Tbx1+/+	3.234229662	10	1.867283366	0.008956536	0.072454791	FALSE
15	PdgfraCre;Tbx1+/flc	PdgfraCre;Tbx1	-0.620273775	16	-0.294221685	0.543811849	0.699186664	FALSE
	PdgfraCre;Tbx1+/flc	WT;Tbx1+/flox	-2.973663831	14	-1.535593399	0.010063165	0.072454791	FALSE
	PdgfraCre;Tbx1+/flc	WT;Tbx1+/+	0.259805567	14	0.134163018	0.798795484	0.860777937	FALSE
	PdgfraCre;Tbx1+/+	WT;Tbx1+/flox	-1.712823843	12	-0.925030599	0.1124384	0.252986399	FALSE
	PdgfraCre;Tbx1+/+	WT;Tbx1+/+	0.780634653	12	0.421590897	0.450142746	0.623274571	FALSE
	WT;Tbx1+/flox	WT;Tbx1+/+	4.274352212	10	2.4677984	0.001625802	0.02926443	TRUE
20	PdgfraCre;Tbx1+/flc	PdgfraCre;Tbx1	0.160447456	16	0.076106911	0.874536855	0.899523622	FALSE
	PdgfraCre;Tbx1+/flc	WT;Tbx1+/flox	-2.501145356	14	-1.291585908	0.025410373	0.143166836	FALSE
	PdgfraCre;Tbx1+/flc	WT;Tbx1+/+	1.09363096	14	0.5647486	0.292577527	0.457947434	FALSE
	PdgfraCre;Tbx1+/+	WT;Tbx1+/flox	-1.803787117	12	-0.974156382	0.09640862	0.231380689	FALSE
	PdgfraCre;Tbx1+/+	WT;Tbx1+/+	0.644726307	12	0.348192001	0.531236864	0.699186664	FALSE
	WT;Tbx1+/flox	WT;Tbx1+/+	3.563351857	10	2.057302154	0.005152424	0.061829092	FALSE
25	PdgfraCre;Tbx1+/flc	PdgfraCre;Tbx1	0.384737119	16	0.182496839	0.705498097	0.79368536	FALSE
	PdgfraCre;Tbx1+/flc	WT;Tbx1+/flox	-2.35775279	14	-1.217538305	0.033465057	0.143166836	FALSE
	PdgfraCre;Tbx1+/flc	WT;Tbx1+/+	0.786078068	14	0.405928969	0.444923628	0.623274571	FALSE
	PdgfraCre;Tbx1+/+	WT;Tbx1+/flox	-2.283065064	12	-1.232996056	0.041448022	0.143166836	FALSE
	PdgfraCre;Tbx1+/+	WT;Tbx1+/+	0.395110604	12	0.213384114	0.699694601	0.79368536	FALSE
	WT;Tbx1+/flox	WT;Tbx1+/+	2.291444377	10	1.322966028	0.044903228	0.143166836	FALSE
30	PdgfraCre;Tbx1+/flc	PdgfraCre;Tbx1	-0.51135554	16	-0.24255723	0.616086234	0.739303481	FALSE
	PdgfraCre;Tbx1+/flc	WT;Tbx1+/flox	-2.091602839	14	-1.080099061	0.055183309	0.152815317	FALSE
	PdgfraCre;Tbx1+/flc	WT;Tbx1+/+	0.591033005	14	0.305208131	0.563919383	0.700037854	FALSE
	PdgfraCre;Tbx1+/+	WT;Tbx1+/flox	-1.190208127	12	-0.642785854	0.256978702	0.440534918	FALSE
	PdgfraCre;Tbx1+/+	WT;Tbx1+/+	0.897571615	12	0.484744074	0.387067972	0.580601957	FALSE
	WT;Tbx1+/flox	WT;Tbx1+/+	2.306159612	10	1.331461873	0.043792782	0.143166836	FALSE

						Shapiro-Wilk test		Levene test				Linear Mixed Model						Fisher's r-to-Z transformation					
Figure	Measurement	Genotype	# of Samples	Average	s.e.m	Statistic	p_value	Statistic	df1	df2	p_value	factor	F	df1	df2	p_value	Measurement	N	Pearson's r	p_value (correlation)	Z transformed correlation	Z statistic	p_value (Z statistic)
Figure 9B	g-ratio	oTbx1+/+	4423	0.742132168	0.001178885	0.983206272	1.62E-22	1.670711318	1	6738	0.196207608	Genotype	0.055858571	1	6736	0.813172794	g-ratio vs Axon Diameter (oTbx1+/+)	4423	0.626809	0.00E+00	0.736142587	-1.466330096	0.14255838
		oTbx1+/-	2317	0.736721572	0.001594201	0.98563242	1.56E-14					Axon Diameter	4117.938128	1	6736	0	g-ratio vs Axon Diameter (oTbx1+/-)	2317	0.649115	2.53E-277	0.773767499		
												Genotype:Axon Diameter	1.497334759	1	6736	0.22112471							

Figure	Measurement	Axon Diameter	Genotype	# of samples	Average	s.e.m	Shapiro-Wilk test statistic	p_value	p_adj(FDR BH)	Rejects (FDR 5%)
Figure 9C	Axon Number	200	oTbx1+/+	145	0.131034483	0.035632184	0.341078043	8.80283E-23	5.47732E-22	TRUE
			oTbx1+/-	67	0.119402985	0.045226142	0.358457863	1.42881E-15	3.63697E-15	TRUE
		300	oTbx1+/+	145	0.662068966	0.111254037	0.56666255	6.46568E-19	2.78522E-18	TRUE
			oTbx1+/-	67	1.313432836	0.207512627	0.771918416	7.85361E-09	1.25658E-08	TRUE
		400	oTbx1+/+	145	1.8	0.180622593	0.794955611	5.68057E-13	1.13611E-12	TRUE
			oTbx1+/-	67	3.268656716	0.268656716	0.922051668	0.000442592	0.000495703	TRUE
		500	oTbx1+/+	145	3.04137931	0.201497236	0.927050591	9.02246E-07	1.29553E-06	TRUE
			oTbx1+/-	67	4.253731343	0.309171785	0.915763259	0.000238829	0.000278634	TRUE
		600	oTbx1+/+	145	4.020689655	0.224134984	0.938970745	6.20047E-06	8.26729E-06	TRUE
			oTbx1+/-	67	5.059701493	0.405123562	0.891388595	2.6243E-05	3.26579E-05	TRUE
		700	oTbx1+/+	145	3.806896552	0.19605705	0.943566203	1.3749E-05	1.79057E-05	TRUE
			oTbx1+/-	67	4.865671642	0.359591407	0.867092669	3.69424E-06	5.04579E-06	TRUE
		800	oTbx1+/+	145	3.593103448	0.177654081	0.944682121	1.67654E-05	2.13378E-05	TRUE
			oTbx1+/-	67	3.671641791	0.28484093	0.922504842	0.000463096	0.000508498	TRUE
		900	oTbx1+/+	145	3.220689655	0.160658656	0.954623818	0.000107914	0.000128578	TRUE
			oTbx1+/-	67	3.014925373	0.261758774	0.920220673	0.000369011	0.000421727	TRUE
		1000	oTbx1+/+	145	2.668965517	0.155380873	0.935009897	3.20007E-06	4.4801E-06	TRUE
			oTbx1+/-	67	2.432835821	0.207528892	0.930607498	0.001061447	0.001143097	TRUE
		1100	oTbx1+/+	145	1.972413793	0.103086853	0.925580502	7.20232E-07	1.09008E-06	TRUE
			oTbx1+/-	67	1.880597015	0.19667336	0.894105256	3.31308E-05	4.03331E-05	TRUE
		1200	oTbx1+/+	145	1.448275862	0.097345533	0.886540353	3.87729E-09	6.38613E-09	TRUE
			oTbx1+/-	67	0.970149254	0.114467183	0.829086244	2.4814E-07	3.85995E-07	TRUE
		1300	oTbx1+/+	145	1.144827586	0.093351451	0.832913399	1.46897E-11	2.65362E-11	TRUE
			oTbx1+/-	67	1.029850746	0.112474228	0.847671866	8.85128E-07	1.29553E-06	TRUE
		1400	oTbx1+/+	145	0.910344828	0.080656574	0.778914571	1.6278E-13	3.37617E-13	TRUE
			oTbx1+/-	67	0.656716418	0.113638558	0.720096111	5.37756E-10	9.12555E-10	TRUE
		1500	oTbx1+/+	145	0.537931034	0.067820962	0.664089799	9.36116E-17	2.66433E-16	TRUE
			oTbx1+/-	67	0.47761194	0.104961083	0.599906087	3.1492E-12	6.08121E-12	TRUE
		1600	oTbx1+/+	145	0.372413793	0.050802777	0.625598192	1.17036E-17	3.8677E-17	TRUE
			oTbx1+/-	67	0.373134328	0.070006205	0.64398396	1.80164E-11	3.15287E-11	TRUE
		1700	oTbx1+/+	145	0.317241379	0.042326328	0.607726872	4.70168E-18	1.7553E-17	TRUE
			oTbx1+/-	67	0.343283582	0.072283423	0.60768044	4.24081E-12	7.91618E-12	TRUE
		1800	oTbx1+/+	145	0.262068966	0.045926697	0.525665522	1.0219E-19	4.76887E-19	TRUE
			oTbx1+/-	67	0.164179104	0.050313423	0.439795375	1.44797E-14	3.24346E-14	TRUE
		1900	oTbx1+/+	145	0.124137931	0.029169144	0.382323027	3.6823E-22	2.06209E-21	TRUE
			oTbx1+/-	67	0.179104478	0.05176825	0.462751687	2.90876E-14	6.26502E-14	TRUE
		2000	oTbx1+/+	145	0.151724138	0.032944622	0.41921103	1.4113E-21	7.1848E-21	TRUE
			oTbx1+/-	67	0.134328358	0.041974785	0.403025091	4.94005E-15	1.15268E-14	TRUE
		2100	oTbx1+/+	145	0.096551724	0.026486709	0.326505065	5.39777E-23	3.77844E-22	TRUE
			oTbx1+/-	67	0.134328358	0.047054932	0.38790673	3.21939E-15	7.83851E-15	TRUE
		2200	oTbx1+/+	145	0.082758621	0.024958679	0.294410527	1.89092E-23	1.51274E-22	TRUE
			oTbx1+/-	67	0.074626866	0.032347005	0.289320052	2.35055E-16	6.26812E-16	TRUE
		2300	oTbx1+/+	145	0.027586207	0.013648669	0.151343524	2.64901E-25	2.96689E-24	TRUE
			oTbx1+/-	67	0	0	1	1	1	FALSE
		2400	oTbx1+/+	145	0.055172414	0.021395989	0.218779445	1.83508E-24	1.71274E-23	TRUE
			oTbx1+/-	67	0.059701493	0.036094986	0.207609415	3.26996E-17	1.01732E-16	TRUE
		2500	oTbx1+/+	145	0.027586207	0.013648669	0.151343524	2.64901E-25	2.96689E-24	TRUE
			oTbx1+/-	67	0.029850746	0.020947148	0.162513554	1.17412E-17	3.8677E-17	TRUE
		2600	oTbx1+/+	145	0.006896552	0.006896552	0.056594372	2.12105E-26	5.93893E-25	TRUE
			oTbx1+/-	67	0.059701493	0.02916443	0.252678573	9.51548E-17	2.66433E-16	TRUE
		2700	oTbx1+/+	145	0	0	1	1	1	FALSE
			oTbx1+/-	67	0.014925373	0.014925373	0.10137707	3.12269E-18	1.24907E-17	TRUE
		2800	oTbx1+/+	145	0.013793103	0.009719273	0.093628466	5.5507E-26	1.03613E-24	TRUE
			oTbx1+/-	67	0	0	1	1	1	FALSE
		2900	oTbx1+/+	145	0.006896552	0.006896552	0.056594372	2.12105E-26	5.93893E-25	TRUE
			oTbx1+/-	67	0	0	1	1	1	FALSE

Axon Diameter	Levene test Statistic	df1	df2	p_value	p_adj(FDR BH)	Rejects (FDR 5%)	Mann-Whitney U test Statistic	R (Z score)	p_value	p_adj(FDR BH)	Rejects (FDR 5%)	Mann-Whitney U test (300-1400nm) Statistic	R (Z score)	p_value	p_adj(FDR BH)	Rejects (FDR 5%)
200	0.036618062	1	210	0.848429647	0.961107197	FALSE	4856	-0.000248086	0.996365794	0.996365794	FALSE					
300	8.05053552	1	210	0.004994777	0.139853743	FALSE	3574.5	-0.212196465	0.00037454	0.00524356	TRUE	3574.5	-0.212196465	0.00037454	0.00224724	TRUE
400	0.146243949	1	210	0.70253768	0.961107197	FALSE	2713	-0.354680685	1.48147E-07	4.14811E-06	TRUE	2713	-0.354680685	1.48147E-07	1.77776E-06	TRUE
500	0.171565279	1	210	0.679147179	0.961107197	FALSE	3448.5	-0.233035713	0.00062617	0.00584425	TRUE	3448.5	-0.233035713	0.00062617	0.002504679	TRUE
600	1.592773869	1	210	0.20833013	0.729155453	FALSE	4021	-0.138349449	0.042188689	0.147660412	FALSE	4021	-0.138349449	0.042188689	0.072323467	FALSE
700	0.742076712	1	210	0.389978983	0.880618111	FALSE	3861.5	-0.16472929	0.015562111	0.087147822	FALSE	3861.5	-0.16472929	0.015562111	0.037349066	TRUE
800	0.310912228	1	210	0.577714827	0.951530303	FALSE	4841	0.002728949	0.968922578	0.996365794	FALSE	4841	0.002728949	0.968922578	0.968922578	FALSE
900	0.7278997	1	210	0.394536889	0.880618111	FALSE	4439	0.069216072	0.308357041	0.626862409	FALSE	4439	0.069216072	0.308357041	0.411142721	FALSE
1000	0.684857725	1	210	0.408858409	0.880618111	FALSE	4552.5	0.05044421	0.456622851	0.741419141	FALSE	4552.5	0.05044421	0.456622851	0.547947421	FALSE
1100	6.478227093	1	210	0.011638138	0.162933933	FALSE	4436	0.069712245	0.298569389	0.626862409	FALSE	4436	0.069712245	0.298569389	0.411142721	FALSE
1200	3.69443342	1	210	0.055947631	0.391633419	FALSE	3707	0.190282177	0.003824339	0.026770374	TRUE	3707	0.190282177	0.003824339	0.011473017	TRUE
1300	0.321086438	1	210	0.571560581	0.951530303	FALSE	4732.5	0.020673857	0.752577581	0.996365794	FALSE	4732.5	0.020673857	0.752577581	0.820993725	FALSE
1400	0.011174349	1	210	0.915914309	0.961107197	FALSE	3984	0.144468911	0.023473363	0.093893452	FALSE	3984	0.144468911	0.023473363	0.046946726	TRUE
1500	0.241876622	1	210	0.623367675	0.961107197	FALSE	4530.5	0.054082809	0.358207091	0.626862409	FALSE					
1600	6.61207E-05	1	210	0.993519823	0.993519823	FALSE	4780.5	-0.012735096	0.820278888	0.996365794	FALSE					
1700	0.107856086	1	210	0.742925516	0.961107197	FALSE	4852	0.00090965	0.987880394	0.996365794	FALSE					
1800	1.66949102	1	210	0.197746792	0.729155453	FALSE	4529	0.054330895	0.249807126	0.626862409	FALSE					
1900	0.981731842	1	210	0.322911885	0.880618111	FALSE	4626.5	-0.038205287	0.34420146	0.626862409	FALSE					
2000	0.095627397	1	210	0.757448003	0.961107197	FALSE	4831	0.004382858	0.916278431	0.996365794	FALSE					
2100	0.561931824	1	210	0.454321862	0.908643725	FALSE	4710.5	-0.024312455	0.495563538	0.741419141	FALSE					
2200	0.036086921	1	210	0.8495196	0.961107197	FALSE	4849	0.001405822	0.966419652	0.996365794	FALSE					
2300	1.882778001	1	210	0.171482662	0.729155453	FALSE	4723.5	0.022162374	0.17251315	0.483036819	FALSE					
2400	0.012885239	1	210	0.90973217	0.961107197	FALSE	4842.5	0.002480863	0.924256179	0.996365794	FALSE					
2500	0.008464882	1	210	0.92678194	0.961107197	FALSE	4846.5	-0.001819299	0.929852964	0.996365794	FALSE					
2600	5.644026015	1	210	0.018414322	0.171867004	FALSE	4601	-0.042422754	0.019005936	0.088694366	FALSE					
2700	2.176243568	1	210	0.14165388	0.729155453	FALSE	4785	-0.011990837	0.144023867	0.448074253	FALSE					
2800	0.928222721	1	210	0.336433275	0.880618111	FALSE	4790.5	0.011081187	0.338862118	0.626862409	FALSE					
2900	0.460888365	1	210	0.497956586	0.929518961	FALSE	4824	0.005540594	0.503105846	0.741419141	FALSE					

LMM (setting session id as random effect) Source	F	df1	df2	Sig.
Corrected Model	133.9074722	55	5880	0
genotype	7.655858881	1	5880	0.005676512
binned_Axon_Diameter	246.5223833	27	5880	0
genotype * binned_Axon_Diameter	6.336275377	27	5880	0

LMM (setting both mouse id and session id as random effects) Source	F	df1	df2	Sig.
Corrected Model	133.7784885	55	5880	0
genotype	0.561757486	1	5880	0.453582974
binned_Axon_Diameter	246.5223833	27	5880	0
genotype * binned_Axon_Diameter	6.336275377	27	5880	0

Measurement	Genotype	# of Samples	Average	s.e.m	Shapiro-Wilk test Statistic	p_value	Levene test Statistic	df1	df2	p_value	Mann-Whitney U test Statistic	p_value	Alternative (H1)
Axon Diameter (nm)	oTbx1+/+	4423	913.5795028	5.511747597	0.929378211	1.60238E-41	2.629408218	1	6738	0.104947594	5790975.5	7.48066E-19	greater
	oTbx1+/-	2317	845.8037375	7.58800961	0.897995234	9.79998E-37							(oTbx1+/+ > oTbx1+/-)

Figure	Chi squared test				Kolmogorov-Smirnov test	
	# of samples	Statistic	dof	p_value	Distance	p_value
Figure 9D	6740	109.7085231	27	6.22E-12	0.108947998	3.59E-16

							Shapiro-Wilk test				Levene test						Mann-Witney U test						
Figure	Measurement	PND	Genotype	# of Samples	Average	s.e.m	Statistic	p_value	p_adj(FDR BH)	Rejects(FDR 5%)	Statistic	df1	df2	p_value	p_adj(FDR BH)	Rejects(FDR 5%)	Sample1	Sample2	Statistic	R (Z score)	p_value	p_adj(FDR BH)	Rejects(FDR 5%)
Figure S1	Weight	P8	oTbx1+/+	27	4.6	0.125064086	0.922948078	0.046515575	0.186062298	FALSE	0.639894198	1	36	0.428996273	0.428996273	FALSE	oTbx1+/+	oTbx1+/-	140	0.044382036	0.799797921	0.91732983	FALSE
			oTbx1+/-	11	4.454545455	0.237931861	0.980904629	0.970981671	0.970981671	FALSE													
		P12	oTbx1+/+	30	6.54	0.155482194	0.986169448	0.955380174	0.970981671	FALSE	1.973777187	1	40	0.167766267	0.428996273	FALSE	oTbx1+/+	oTbx1+/-	153	0.115996334	0.465855659	0.91732983	FALSE
			oTbx1+/-	12	6.225	0.351861931	0.915390828	0.249993706	0.499987413	FALSE													
		1M	oTbx1+/+	27	16.15925926	0.252241194	0.863150358	0.002121269	0.016970151	TRUE	0.856989714	1	36	0.360747027	0.428996273	FALSE	oTbx1+/+	oTbx1+/-	135.5	-0.067878408	0.703523286	0.91732983	FALSE
			oTbx1+/-	11	16.37272727	0.447232075	0.942333972	0.548313562	0.80806296	FALSE													
		2M	oTbx1+/+	21	23.78571429	0.385837723	0.943028092	0.24996769	0.499987413	FALSE	1.523319488	1	29	0.227026429	0.428996273	FALSE	oTbx1+/+	oTbx1+/-	102	0.022769128	0.91732983	0.91732983	FALSE
			oTbx1+/-	10	23.53	0.736515822	0.94467026	0.60604722	0.80806296	FALSE													

						Shapiro-Wilk test				Levene test						Mann-Witney U test						
Measurement	PND	Genotype	# of Samples	Average	s.e.m	Statistic	p_value	p_adj(FDR BH)	Rejects(FDR 5%)	Statistic	df1	df2	p_value	p_adj(FDR BH)	Rejects(FDR 5%)	Sample1	Sample2	Statistic	R (Z score)	p_value	p_adj(FDR BH)	Rejects(FDR 5%)
Weight	P8	PdgfraCre;WT	8	4.35	0.205287255	0.866045239	0.137839188	0.413517564	FALSE	0.127476314	2	24	0.880906332	0.880906332	FALSE	PdgfraCre;WT	WT;Tbx1+/flox	18	-0.367573522	0.160528361	0.872562731	FALSE
		WT;Tbx1+/flox	8	4.7375	0.203485257	0.753919531	0.009024632	0.054147793	FALSE							PdgfraCre;WT	WT;WT	29.5	-0.274679021	0.272287052	0.872562731	FALSE
		WT;WT	11	4.681818182	0.225153758	0.91199385	0.257472583	0.617934199	FALSE							WT;Tbx1+/flox	WT;WT	40.5	-0.066301833	0.840385277	1	FALSE
	P12	PdgfraCre;WT	9	6.477777778	0.227777778	0.921953577	0.408659184	0.700558602	FALSE	1.743649374	2	27	0.194001478	0.776005912	FALSE	PdgfraCre;WT	WT;Tbx1+/flox	30.5	-0.20813018	0.436281366	0.872562731	FALSE
		WT;Tbx1+/flox	9	6.788888889	0.217590623	0.947010301	0.657291765	0.876389021	FALSE							PdgfraCre;WT	WT;WT	52.5	0.023262105	0.97227231	1	FALSE
		WT;WT	12	6.4	0.31454633	0.994973684	0.999999306	0.999999306	FALSE							WT;Tbx1+/flox	WT;WT	42	0.186096842	0.422107304	0.872562731	FALSE
	1M	PdgfraCre;WT	8	16.3625	0.287189273	0.934767327	0.560437988	0.840656982	FALSE	0.176982173	2	24	0.838878203	0.880906332	FALSE	PdgfraCre;WT	WT;Tbx1+/flox	31	0.116690007	0.672974085	1	FALSE
		WT;Tbx1+/flox	9	15.78888889	0.590537525	0.763341553	0.007737799	0.054147793	FALSE							PdgfraCre;WT	WT;WT	40	0	1	1	FALSE
		WT;WT	10	16.33	0.381240665	0.925384317	0.404055022	0.700558602	FALSE							WT;Tbx1+/flox	WT;WT	42	-0.056195149	0.842105263	1	FALSE
	2M	PdgfraCre;WT	8	24.3875	0.578309575	0.794950867	0.025269633	0.101078534	FALSE	0.889390233	2	18	0.428210148	0.856420296	FALSE	PdgfraCre;WT	WT;Tbx1+/flox	26	0.05976143	0.866511267	1	FALSE
		WT;Tbx1+/flox	7	23.91428571	0.525279323	0.958737507	0.807832827	0.969399392	FALSE							PdgfraCre;WT	WT;WT	14.5	0.327781141	0.282384282	0.872562731	FALSE
		WT;WT	6	22.83333333	0.889818958	0.974279891	0.919850787	0.999999306	FALSE							WT;Tbx1+/flox	WT;WT	14	0.277350098	0.365967366	0.872562731	FALSE

*including P11 and P13

**At the beginning of the behavioral battery (RSI) at each age

						Shapiro-Wilk test		Levene test				Linear Mixed Model						Fisher's r-to-Z transformation					
Figure	Measurement	Genotype	# of Samples	Average	s.e.m	Statistic	p_value	Statistic	df1	df2	p_value	factor	F	df1	df2	p_value	Measurement	N	Pearson's r	p_value (correlation)	Z transformed correlation	Z statistic	p_value (Z statistic)
Figure S2	myelin thickness (nm)	oTbx1+/+	4423	298.4267488	1.598178651	0.883548319	0.00E+00	18.9486008	1	6738	1.36273E-05	Genotype	0.007407354	1	6736	0.931416522	myelin thickness vs Axon Diameter (oTbx1+/+)	4423	0.309679	6.35E-99	0.320189803	-2.190862775	0.028461724
		oTbx1+/-	2317	281.3171899	1.943145919	0.89264971	1.85E-37					Axon Diameter	755.8280666	1	6736	0	myelin thickness vs Axon Diameter (oTbx1+/-)	2317	0.359582	1.15E-71	0.376405671		
												Genotype:Axon Diameter	0.05543768	1	6736	0.813865028							
